# Interpreting whole genome and exome sequencing data of individual gastric cancer samples

**DOI:** 10.1186/s12864-017-3895-z

**Published:** 2017-07-06

**Authors:** Daniela Esser, Niklas Holze, Jochen Haag, Stefan Schreiber, Sandra Krüger, Viktoria Warneke, Philip Rosenstiel, Christoph Röcken

**Affiliations:** 1 Institute for Clinical Molecular Biology, Christian-Albrechts-University, 24105 Kiel, Germany; 2 Institute of Pathology, Christian-Albrechts-University, Arnold-Heller-Str. 3, Haus 14, D-24105 Kiel, Germany; 3 Department of General Internal Medicine, University Hospital Schleswig-Holstein, 24105 Kiel, Germany; 4 Institute for Experimental Medicine, Christian-Albrechts-University, 24105 Kiel, Germany

**Keywords:** Gastric cancer, Deep sequencing, GNAS

## Abstract

**Background:**

Gastric cancer is the fourth most common cancer and the second leading cause of cancer death worldwide. In order to understand the genetic background, we sequenced the whole exome and the whole genome of one microsatellite stable as well as one microsatellite unstable tumor and the matched healthy tissue on two different NGS platforms. We here aimed to provide a comparative approach for individual clinical tumor sequencing and annotation using different sequencing technologies and mutation calling algorithms.

**Results:**

We applied a population-based whole genome resource as a novel pathway-based filter for interpretation of genomic alterations from single nucleotide variations (SNV), indels, and large structural variations. In addition to a comparison with tumor genome database resources and a filtering approach using data from the 1000 Genomes Project, we performed pyrosequencing analysis and immunohistochemistry in a large cohort of 428 independent gastric cancer cases.

**Conclusion:**

We here provide an example comparing the usefulness and potential pitfalls of different technologies for a clinical interpretation of genomic sequence data of individual gastric cancer samples. Using different filtering approaches, we identified a multitude of novel potentially damaging mutations and could show a validated association between a mutation in *GNAS* and gastric cancer.

**Electronic supplementary material:**

The online version of this article (doi:10.1186/s12864-017-3895-z) contains supplementary material, which is available to authorized users.

## Background

In recent decades we witnessed major advancements in the understanding of the epidemiology, pathology and pathogenesis of gastric cancer (GC), which were accompanied by the introduction of chemotherapy for the treatment of GC [1–3]. However, there is overwhelming evidence from a variety of cancers that patient prognosis and treatment responses do not only depend on tumor stage but also on phenotypic and genotypic tumor characteristics. The emergence of ultrahigh-throughput sequencing [next generation sequencing (NGS)] has enabled researchers to acquire genome-wide insights into the mutational landscape of individual tumors at base-pair resolution. A number of large scale genomic screens for single nucleotide mutations or structural variants in selected tumor genomes have been published demonstrating the feasibility of this approach [4–9]. All data sets have demonstrated an unforeseen complexity of the mutational landscape and have demonstrated the extensive dynamics behind tumor initiation and progression. However, many of these studies have analyzed predominantly coding variants, as they are more accessible to prediction and interpretation. In this study we describe the genetic architecture of two independent GC samples. We employed paired tumor/normal tissue analysis and combined both exome and whole genome sequence information. The study combines single nucleotide variation (SNV), indels as well as structural variant analysis, integrates data from two different sequencing platforms and can be used as a blueprint for further individual cancer genome analyses. We employed several scoring systems in parallel including a new exonic conservation score (ECS) and revealed pathways with an unexpectedly high number of deleterious polymorphisms and somatic mutations. Using this comprehensive strategy we were able to identify a multitude of novel potentially damaging mutations, which were partially validated in an independent cohort of 482 GC patients.

**Table 1:** Clinico-pathological patient characteristics of the gastric cancer validation cohort

Patient characteristics				
Patients		[n]	482	
Age [years]		[mean ± SD]	67.9 ± 11.1	
		median	68	
Gender	Men	[n (%)]	297	(61.6)
	Women	[n (%)]	185	(38.4)
Follow-up data	Alive	[n (%)]	131	(28.1)
	Dead	[n (%)]	335	(71.9)
Localization	Proximal	[n (%)]	149	(30.9)
	Distal	[n (%)]	333	(69.1)
pT-category	pT1a	[n (%)]	13	(2.7)
	pT1b	[n (%)]	49	(10.2)
	pT2	[n (%)]	56	(11.6)
	pT3	[n (%)]	190	(39.4)
	pT4a	[n (%)]	134	(27.8)
	pT4b	[n (%)]	40	(8.3)
pN-category	pN0	[n (%)]	138	(28.8)
	pN1	[n (%)]	67	(14.0)
	pN2	[n (%)]	85	(17.7)
	pN3/a/b	[n (%)]	189	(39.5)
UICC Stage (7th ed.)	IA	[n (%)]	49	(10.4)
	IB	[n (%)]	32	(6.8)
	IIA	[n (%)]	58	(12.3)
	IIB	[n (%)]	47	(9.9)
	IIIA	[n (%)]	55	(11.6)
	IIIB	[n (%)]	83	(17.5)
	IIIC	[n (%)]	66	(14.0)
	IV	[n (%)]	83	(17.5)
Stage according to	I	[n (%)]	49	(10.2)
Kiel proposal	II	[n (%)]	84	(17.5)
	IIIA	[n (%)]	49	(10.2)
	IIIB	[n (%)]	153	(31.9)
	IV	[n (%)]	145	(30.2)
Resected lymph nodes		[mean ± SD]	19.2 ± 8.2	
		median [n]	18	
Positive lymph nodes		[mean ± SD]	6.4 ± 7.4	
		median [n]	3	
Lymph node ratio (LNR)		median [n]	0.2	
Tumor grade	G1 / G2	[n (%)]	111	(23.7)
	G3 / G4	[n (%)]	357	(76.3)
Resection margin (R-status)	R0	[n (%)]	403	(88.2)
	R1/R2	[n (%)]	54	(11.8)

## Results

**Fig. 1:**
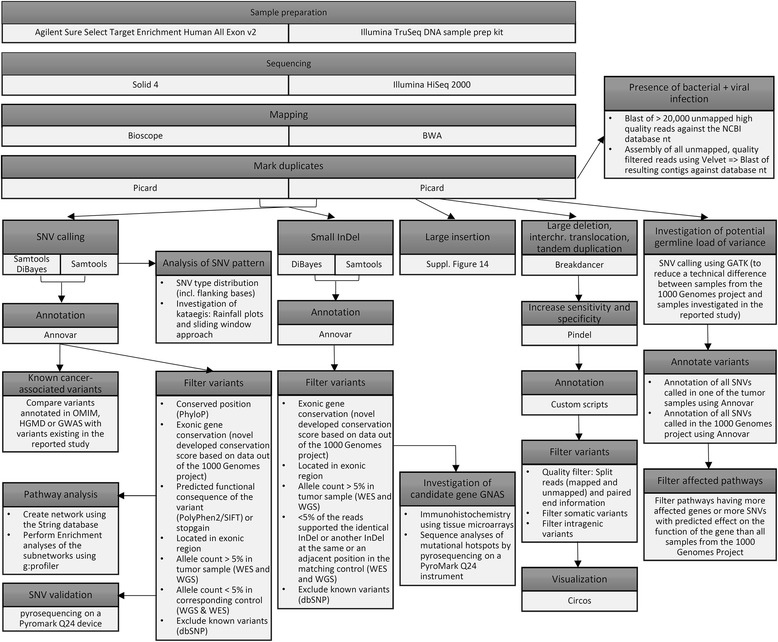
Study workflow

The study workflow is summarized in Fig. 1.

### Study patients

Two patient samples were retrieved for deep sequencing analysis.

Patient No. 1 (74-years old Caucasian woman) had died from a moderately differentiated, highly microsatellite unstable, intestinal type GC of the antral mucosa (tumor stage: pT3 pN0 (0/20) L0 V0 R0 G2).

Patient No. 2 (74-years old Caucasian woman) had died from a moderately differentiated, microsatellite stable, intestinal type GC of the antral mucosa (tumor stage: pT4 pN1 (2/21) L0 V0 R0 G2).

### Sequencing and mapping results

**Fig. 2:**
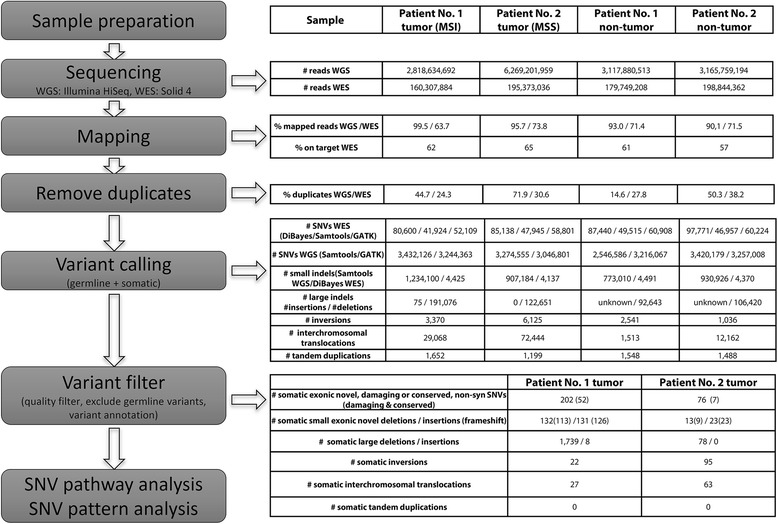
Workflow of whole genome and whole exome sequencing and subsequent data analysis

The whole exome sequencing (WES, Solid) produced between 160 and 199 million paired reads with a length of 50/35 bp per sample, which resulted in a mean coverage between 41- and 66-fold. The mean library insert size for all pairs with a distance smaller 2000 (96–98% of all pairs) was between 184 and 286 bp. The output of the whole genome sequencing (WGS, Illumina) contained between 2.8 and 6.2 billion paired reads with a read length of 100 bp, which resulted in a mean coverage of 14- to 78-fold (Fig. 2, Additional file 1: Figure S1). The mean library insert size for all pairs with a distance smaller 2000 (85–99% of all pairs) was between 180 and 215. Although the number of covered bases was lower in the non-tumor sample of the second patient (WGS data), all reported filtered variants were covered by at least three reads in both data sets of the second patient. It has to be noted that the coverage in the WES data of the MSI tumor was lower than in the other data sets. This could have led to a detection lack of some variants, but had no influence on the reported variants and the described mutational patterns.

The sequencing results confirmed that no viral or bacterial infection was present in the tumor samples.

### SNV and small indel calling

Several variant caller were used and the detected SNVs and small indels filtered by mutant allele fractions afterwards. This methodology was applied to identify the most robust call sets. Depending on the sequencing data set and SNV-Caller between 41,924 and 97,771 germline or somatic SNVs were detected in the WES and around three million germline or somatic SNVs in the WGS data (Fig. 2). The number of raw somatic SNVs is shown in Additional file 2: Table S9. The union of all variants called by at least one caller in the WES or WGS data, which have a supporting mutant allele fraction larger 5% in the WES and WGS data, were defined as cross platform variants. All variants called by one of the applied variant callers for one sequencing technology without verification in the second sequencing technology were named single technology variants. Out of the cross platform variants, 202 and 76 filtered SNVs were somatic (i.e. present only in the respective tumor sample) in the MSI tumor of the first patient and the MSS tumor of the second patient, respectively (Additional file 2: Table S2). Based on the mutant allele fractions of somatic SNVs called in the WGS data, the tumor cellularity was estimated to be around 30% for the MSI and 48% for the MSS tumor sample, respectively (Additional file 1: Figure S2).

In the WGS sequencing data, 1,234,100 germline or somatic single technology small indels were detected in the MSI tumor sample and 773,010 germline or somatic single technology indels in the matching control sample of patient 1. In the WES data of patient 1, 4425 and 4491 germline or somatic single technology small indels were identified in the MSI tumor and control sample, respectively. This resulted in 239 cross platform indels, which were somatic, frameshift, and novel. In the WGS data of the second patient, 907,184 germline or somatic single technology small indels were called in the MSS tumor sample and 930,926 in the matching control. In the WES data of the second patient, 4137 germline or somatic single technology small indels were found in the MSS tumor sample, while 4370 germline or somatic single technology small indels were detected in the corresponding control (Fig. 2). Out of this, 32 cross platform small indels were somatic, frameshift, and novel.

### Differences between sequencing technologies and SNV calling methods

To find a valid approach to reduce the number of false-positive and false-negative reported positions in our results, the robustness of different SNV-caller as well as different sequencing strategies were investigated. Less than 50% of the SNVs were called by all callers (DiBayes, Samtools, and GATK) in the WES (Additional file 1: Figure S15, S16 and S17). In the WGS approach, the overlap between Samtools and GATK was between 65% and 88% (Additional file 1: Figure S15), while Samtools exhibited the highest amount of detected variants of the cross platform variants. All SNVs called with Samtools or DiBayes and supported by more than 5% of the mutant allele fractions in the WES and the WGS data were used for further analyses and defined as cross platform variants. Quality filter steps as well as the extraction of somatic variants were based on mutant allele fractions. A large number of SNVs were called in only one sequencing dataset (Additional file 1: Figure S3). Overall, of course more SNVs were called in the WGS than in the WES data. The percentage of somatic SNVs were called in the WES and validated in the WGS data was markedly higher than the number of SNVs called in the WGS and validated in the WES data (Additional file 1: Figure S4). External validation with pyrosequencing of a subset of eight SNVs called in the WES data and not supported by the WGS data failed entirely. Six somatic SNVs called in both WES tumor samples (Additional file 2: Table S6 + S7), which may point clearly to specific problems in either the Solid sequencing platform and/or library preparation protocol. Most of the variants in genes listed in the COSMIC cancer gene census list were called only in WGS or WES (Additional file 2: Table S2 + S3), and only confirmed by mutant allele fractions in the other. This include non-synonymous variants in genes like *PIK3CA*, *JAK2*, *GATA3*, *ROS1*, *ARID1A*, and *BRAF*. Only *DROSHA* was detected by all methods. All reported observations strengthen the benefit of applying a cross platform approach. Further details about the platform comparisons can be found in the Additional file 3: supplementary results.

### Mutational landscape of somatic SNVs in gastric cancer samples

**Fig. 3:**
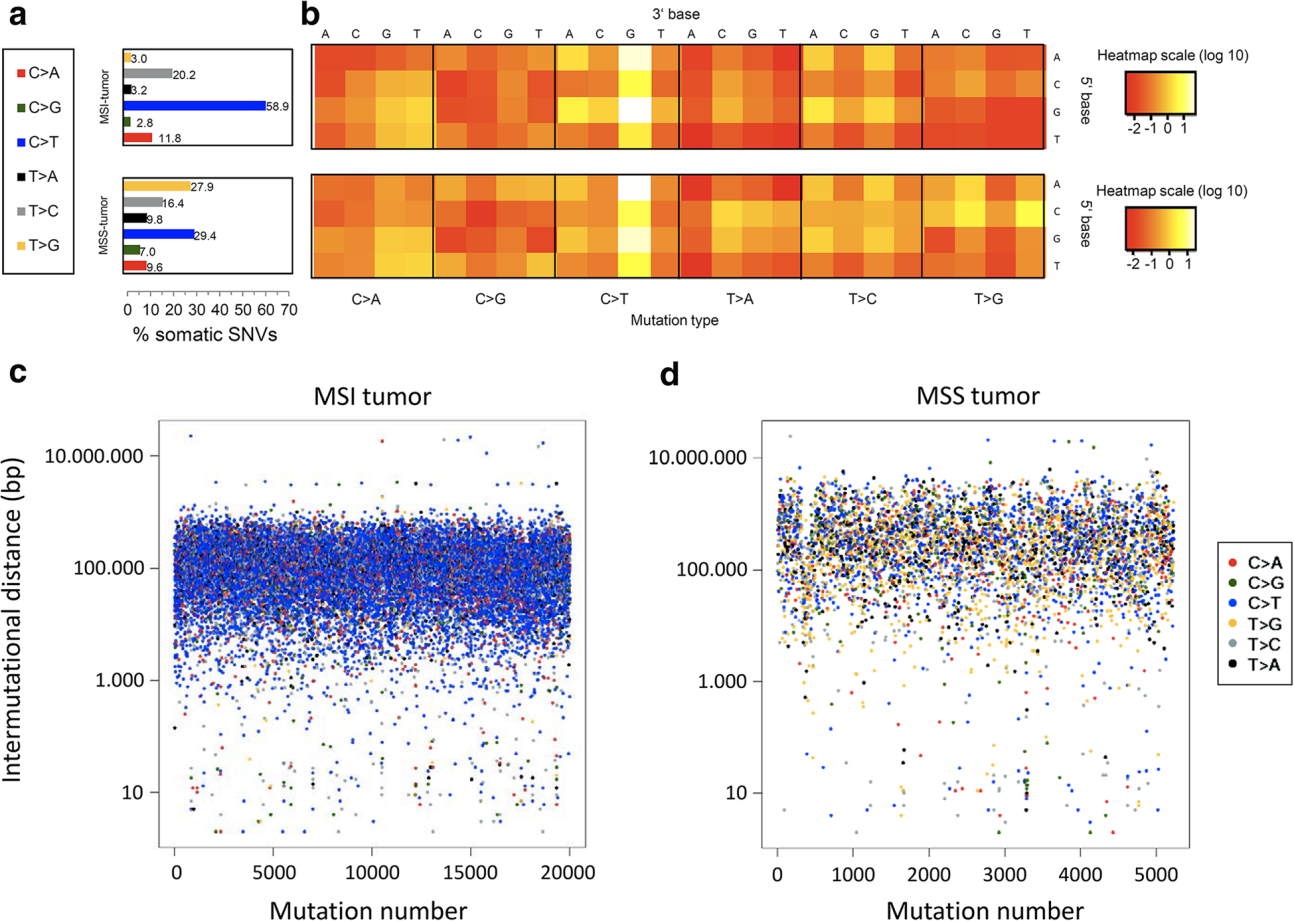
SNV patterns. **a** Relative contribution of the six SNV classes. **b** Heatmap is based on the log-transformed percentage of each SNV type with each SNV context corrected for the frequency of the trinucleotide in the reference genome. **c** + **d** SNV density plots for MSI tumor (**c**) and MSS tumor (**d**). Rainfall plots are based on all somatic SNVs. The x-axis shows the mutations ordered by position. The distance between each mutation and the prior variant is plotted on the y-axis. The colors of the dots indicate the SNV-types

The relative contribution of the six SNV-classes (C > A, C > G, C > T, G > A, G > C, G > T) including the flanking bases were investigated for the somatic variants of the tumor samples called in the WGS data (single technology SNVs) (Fig. 3a + b). We could show an overrepresentation of somatic C > T SNVs, especially in the context of GpCpG and ApCpG, in the MSI tumor. In the MSS tumor a disposition to T > G in context of a five prime cytosine was observed. Furthermore, a decrease of T > C, especially in context of a five prime thymine, was detected.

Regions with a high somatic single technology SNV density, potentially representing kataegis events [10], were visualized by rainfall-plots, in which the distances between each somatic SNV and the previous somatic SNV were plotted (Fig. 3c + d, Additional file 1: Figure S5). Furthermore, a sliding window approach was applied. Based on the results reported in previous studies [11, 12], the following start parameters were chosen: Eight somatic SNVs in a 160 bp window and 50 somatic SNVs in a 5000 bp window. However, in both samples no such intragenic somatic SNV cluster was detected. The total density of somatic SNVs was markedly higher in the MSI GC of patient 1 compared with the MSS GC of patient 2.

In addition, somatic signatures were estimated with the statistical method non-negative matrix factorization (NMF) to identify common underlying patterns. Again, single technology somatic SNVs called in the WGS data were used as input. The first signature was dominated by C > T base substitutions especially in the context of A-G or G-G, while the second signature harbored mainly C > T variants in an A-G context and T > G SNVs in a C-T or C-C context (Additional file 1: Figure S6). The MSS tumor was mainly influenced by the first signature, while the second signature contributed to a large proportion of the SNV pattern observed in the MSI tumor (Additional file 1: Figure S7). It was clearly visible that MSI and MSS harbor distinct somatic SNV patterns. Finally, we compared the somatic SNVs of the investigated tumor samples with the somatic variant calls from WES studies from The Cancer Genome Atlas, which demonstrated special characteristics of GC (Additional file 1: Figure S8A).

Using known mutational signatures reported in the COSMIC database (http://cancer.sanger.ac.uk/cosmic/signatures) we have compared the observed mutational landscapes with known biological processes, which are known to drive specific mutational mechanisms using the package deconstructsigs [13]. While signature 1 (49%), 6 (13%), and 15 (38%) contributed to the mutational pattern of the MSI tumor, the mutational pattern of the MSS tumor was influenced by signature 1 (46%) and 17 (35%) as well as by unknown factors. These processes include ageing/spontaneous deamidation (signature 1, mainly causing C > T), DNA mismatch repair (signature 15, C > T and C > A), signature 17 (unknown, T > G), defective DNA mismatch repair in MSI tumors (signature 6, C > T and C > A). The higher levels of signature 15 (C > T and C > A) in the MSI is consistent with this signature being observed in cases with many indels. In the MSS patient we observed elevated levels of signature 17 (T > G), the underlying mechanism of which is still unclear (Additional file 1: Figure S8B).

### Comparison with known cancer-associated SNVs

The cross platform approach was exploited to minimize the systematic technological bias and thereby to increase the reliability of the variant positions. 277 (271 novel) cross platform non-synonymous exonic somatic SNVs passed all quality filter steps in the MSI tumor and 117 (113 novel) in the MSS tumor (Additional file 1: Figure S9). Out of these SNVs 52 cross platform novel somatic base substitutions in the MSI tumor and seven cross platform novel somatic variants in the MSS tumor were classified as (i) stopgain SNV or (ii) predicted as damaging and were either at a conserved position or in a conserved gene (Additional file 2: Table S2 C + D). Out of these, four genes affected by a somatic SNV in the MSI tumor [*AFF3*, *DROSHA*, *JAK2*, *PIK3CA* (associated with GC)] and one gene affected by a somatic SNV in the MSS tumor (*GATA3*) were also listed in the cancer gene census list of the COSMIC database and not mentioned in ExAc or ESP (exome sequencing database, see methods).

In the sample of the MSI tumor 77 cross platform novel somatic small indels were supported by the mutant allele fractions of WES and WGS data. Out of genes affected by a somatic small indel in the MSS tumor, six were also listed in the cancer gene census table of the COSMIC database including somatic frameshift indels in *BRAF*, *ZFHX3* (both associated with GC), *MAPK1*, and *ARID1A. BRAF* and *ARID1A* were affected by cross platform indels, while indels in ZFHX3 and MAPK1 were exclusively called and covered in the WGS data. Due to the function described in the literature and because of being part of COSMIC’s cancer gene census list, *BRAF* was an especially interesting candidate. Interestingly, in just one sample out of the 1000 Genomes project a germline SNV predicted as damaging was located in *BRAF* and 38 samples harbored a germline frameshift indel in *BRAF*. In all 38 samples the indel was at the same position in the last exon. In contrast, in the MSI tumor sample a cross platform somatic frameshift indel was found in exon 10 (18 exons total). None of the quality filtered somatic indels of the MSS tumor sample existed in the COSMIC’s cancer gene census list. Additionally, 186 single technology somatic indels (165 frameshift) were called with at least two reads in the WGS at positions with coverage smaller than 3× in the WES in the MSI tumor and 34 (31 frameshift) in the MSS tumor (Additional file 2: Table S3). Five of the genes affected by a single technology indel (WGS) in the MSI tumor (*GNAS*, *HOXC13*, *MAPK1*, and *ZFHX3*) and two of the genes affected by a single technology indel (WGS) in the MSS tumor (*GNAS* and *HOXD13*) were also listed in the cancer gene census list of the COSMIC database.

To detect predisposing somatic or germline alleles, cancer-associated variants annotated in the databases GWAS, OMIM or HGMD were compared with all somatic and germline variants called in the tumor samples. In total 301 | 296 known cancer-associated SNVs (germline or somatic) were detected in the tumor samples of patient 1 (MSI) | patient 2 (MSS). However, nearly all variants were germline in the investigated tumor samples and thus considered as potentially predisposing, but not necessarily causative. Further details are described in the supplement. One exceptional somatic mutation in *TP53* was noted (rs28934574, frequency in ExAc_Aggregated_Populations = 0.00001647), which was exclusively detected in the MSS tumor sample and probably represents a somatic mutation at a recurrent position (R282W). This SNV is also associated with the Li-Fraumeni syndrome [14, 15].

In addition, three cross platform SNVs were detected in cancer hotspot regions reported by Chang et al. [16]. Two of them were located in *GRIN2* (T100 and A50, germline SNVs in MSS and MSI tumor) and one in *TP53* (R282, somatic SNV in MSS tumor).

### Novel somatic potentially damaging SNVs and small indels

Beside the detection of novel somatic SNVs and somatic small indels in genes recurrently mutated in tumor samples (described in previous section), cross platform somatic SNVs predicted as damaging at a conserved position were also detected in further promising candidates. These include alterations in e.g. *MSH4*, *PRDM2*, and *TP53I3* in the MSI tumor as well as *GATA*, *DOCK5*, and *IGSF11* in the MSS tumor (Additional file 2: Table S2 + S3).

The following cross platform non-synonymous somatic mutations were validated independently by pyrosequencing on a Pyromark Q24 device (QIAGEN) as third validation step: *DROSHA*, *MSH4*, *RERE*, *ROS1*, *TACC2*, and *TYRO3* (data not shown).

### Structural variations and large indels

To find somatic large insertions with a potential association to GC, all unmapped reads in good quality were de novo assembled for both tumor samples. In the MSI tumor 14,639 contigs were formed, of which 367 had at least one putative insert position. The quality filter passed 75 contigs with on average 1.8 possible insert positions and a maximum length of 81 nucleotides. Eight of these variants were exclusively present in the tumor sample and therefore declared as somatic. The insert length varied between 5 and 31 bp. One somatic insert position was located in the intronic region of the gene *MGAM*, which encodes the maltase-glucoamylase protein. The protein plays a role in the digestion of starch. All other putative somatic insert positions were located in intergenic regions. All putative insert positions with distance smaller 100 KB to the closest gene are reported in the Additional file 2: Table S8). For the MSS tumor no somatic large insertions were detected.

In both tumor samples more inversions including potentially germline and somatic inversions were observed than in the matching controls. In the MSI tumor sample 3370 inversions including potentially germline and somatic inversions were detected, while in the MSS tumor 6125 germline or somatic variants were called. Out of these variants 71 (48 non-overlapping) and 201 (173 non-overlapping) somatic inversions passed the quality filter in the MSI and MSS tumor, respectively. In contrast, 2541/1036 germline or somatic variants were detected in the control sample of patient 1/patient 2, of which 33 (21 non-overlapping)/47 (35 non-overlapping) met the quality criteria. In the MSI tumor 22 (17 non-overlapping) somatic inversions affecting eight genes were detected: *DCK* (intronic), *SPINK14*, *AHCYCL2*, *FAM40B*, *LOC642236*, *CCDC88C*, *LOC440434*, and *CCDC102B*. In the MSS tumor 95 (89 non-overlapping) somatic inversions in 30 genes were identified, of which eight were at least partially located in exonic regions: *WARS2*, *NASP*, *AKR1A1*, *RIMS1*, *EPHA5*, *GRID1*, *KCNMA1*, and *SPINK14*. In both patients a somatic inversion affected the gene *SPINK14*.

In addition, an increased number of interchromosomal translocations including potentially germline and somatic interchromosomal translocations was found in the tumor samples. The program Breakdancer detected 29,068 (60 filtered in 37 regions) germline or somatic interchromosomal translocations in the MSI tumor and 1513 (26/24) germline interchromosomal translocations in the matching control of patient 1. Out of these, 27 breakpoint pairs in 18 regions with 14 affected genes were somatic. Affected genes include *SF3A3*, *DCAF6*, *ANKRD30BL*, *SENP5*, *PDE4D*, *CENPH*, *ACOT13*, *PHACTR2*, *CACNA2D1*, *ABCB1*, *CTNNA3*, *C10orf54*, *CDH23*, and *BAZ2A*. The MSS tumor sample of patient 2 harbored 72,444 (137/74) interchromosomal translocations including potentially germline and somatic interchromosomal translocations, while in the corresponding non-tumor sample 12,162 (61/45) variants were called. Sixty three breakpoint-pairs in 42 regions with 14 affected genes were somatic (Additional file 2: Table S4): *SRRM1*, *GUSBP1*, *MCC*, *CMYA5*, *MBOAT1*, *IMMP2L*, *CNTNAP2*, *MLL3*, *PCMTD1*, *LOC642236*, *TNC*, *CDC27*, *NDUFA13*, and *SLC6A16*. Several breakpoints indicating somatic interchromosomal translocations were located in the gene *MLL3*.

Next, deletions larger than five basepairs were investigated. The following numbers of variants including potentially germline and somatic variants were detected by the program Pindel (all (filtered)): 191,076 (12,173) in the MSI tumor and 92,643 (8892) in the matching control of the first patient, 122,651 (14,224) in the MSS tumor and 106,420 (8096) in the corresponding non-tumor sample of the second patient. Out of 1739 somatic deletions in the MSI tumor of the first patient 343 were located in intragenic regions. Interestingly, out of the somatic deletions over 97% were located on chromosome five, six or seven. The average length of the somatic deletions was 123 bp. In the MSS tumor of the second patient 78 deletions with an average length of 62 bp in 30 gene loci were somatic. All above stated intragenic somatic deletions were located in intronic regions.

The program Pindel detected 1652 (72 filtered/4 non-overlapping filtered) tandem duplications including potentially germline and somatic tandem duplications in the MSI tumor and 1548 (53/2) germline tandem duplications in the matching control sample of the first patient. In the second patient 1199 (69/1) tandem duplications including potentially germline and somatic tandem duplications were called in the MSS tumor and 1488 (74/3) germline tandem duplications in the corresponding non-tumor sample. However, all filtered somatic tandem duplications were intergenic.

**Fig. 4:**
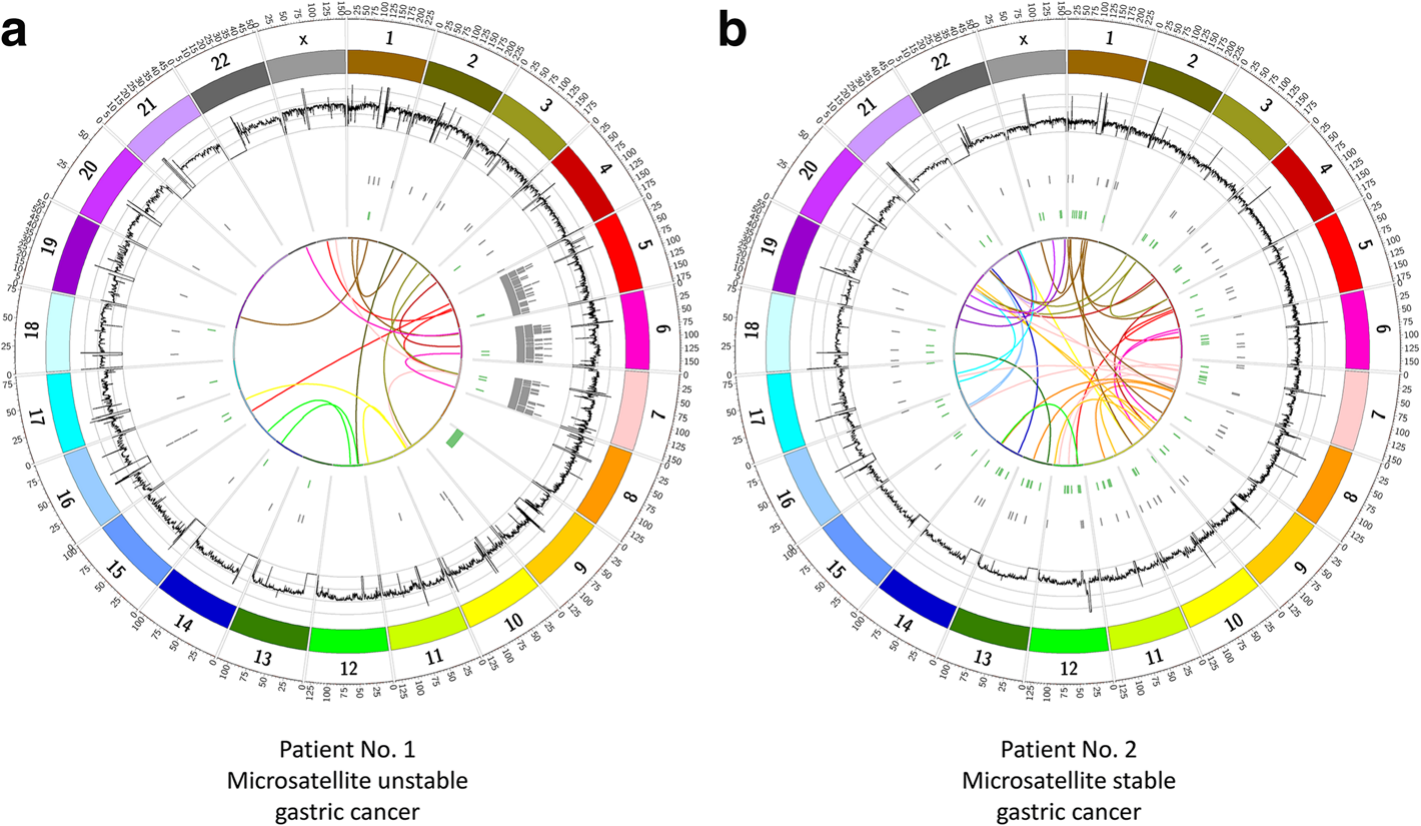
Circos plots of all somatic, quality filtered large structural variants of the MSI (**a**) and MSS (**b**) tumor samples, of patient 1 and 2, respectively. In order from outer to inner rings: genomic position, coverage (*black*), deletions (*grey*), inversions (*green*), and translocations (*center*). The coverage histogram was based on a sliding window, with window size 150,000 and step size 75,000. The maximum axis limit of the coverage histogram was set to 100

All quality filtered, somatic inversions, interchromosomal translocations, tandem duplications and large deletions structural variations are shown in Fig. 4. All filtered somatic intragenic structural variants are shown in Additional file 2: Table S4.

Due to their function described in the literature the most interesting candidates affected by an exonic located somatic structural variant were *MCC* (translocation, patient 2), *MLL3* (translocation, patient 2), *DCK* (inversion, patient 1), *PDE4D* (translocation and deletion, patient 1), and *PARK2* (deletion, patient 1).

### Pathway analyses

For each patient a network was generated using all genes affected by cross platform somatic exonic SNVs, which were a stopgain mutation, or non-synonymous, predicted as damaging or were at a conserved position or in a conserved gene (for definition see Methods). The connections were based on medium stringency according to the STRING database [17]. For the MSI tumor of the first patient, somatic variants formed two networks (Additional file 1: Figure S10 A): (i) The larger one was enriched for genes involved in the response to an external stimulus (*p* = 0.0018), cell communication (*p* = 0.024), and positive regulation of metabolic process (*p* = 0.038) (Additional file 1: Figure S11A). (ii) In contrast, the smaller network harboured genes associated with chromatin organization (*p* = 0.00026) and modification (*p* = 0.00009), positive regulation of biosynthetic processes (*p* = 0.016), and gene expression (*p* = 0.012) as well as cell motility (*p* = 0.0033), and cell migration (*p* = 0.0022) (Additional file 1: Figure S11B). No larger network was detected for the MSS tumor of the second patient (Additional file 1: Figure S10 B).

Additionally, we investigated processes affected by a high load of germline and somatic variants which followed the described criteria as we hypothesized that (rare) germline and somatic mutations together would contribute to malignant transformation. In this approach we corrected for the overall germline spectrum of such variants in all 1092 individuals of the 1000 Genomes project. Nine enriched GO terms were shared between patient 1 (MSI tumor) and 2 (MSS tumor) including the functional interesting terms ‘nuclear migration along microfilament’, ‘negative regulation of transposition’, ‘negative regulation of viral reproduction’, and ‘DNA cytosine deamination’ (see Additional file 3: Supplementary Results and Additional file 1: Figure S12 and S13).

### Identification of genes putatively involved in gastric cancer biology

We next analyzed potentially damaging somatic SNVs, which were localized in a hotspot-region, defined as a recurrently targeted domain in a gene. Three genes were identified, which are known to carry hotspot-mutations in various types of cancer, i.e. *BRAF*, *PIK3CA* and *GNAS*. Our own previous investigations on *BRAF* and *PIK3CA* demonstrated that a mutation in codon 600 of *BRAF* was not found in any of 482 GC patients (Table 1) [18]. Mutations in exon 9 and 20 of *PIK3CA* were found 1.9% and 2.5% of the patients with GC [18]. Interestingly, the *PIK3CA* exon 20 mutations had been more common in MSI GCs [18]. Little data were available for *GNAS* in GC, and therefore, we screened primary tumors of 482 GC patients for the occurrence of somatic mutations in the mutational hotspot region of *GNAS*, i.e. codon 201 in exon 8 and codon 227 in exon 9. Only seven (1.4%) GCs harbored a mutation (Additional file 2: Table S5). GCs with *GNAS* mutation were exclusively of male gender with an intestinal (4 case), diffuse (2) and unclassified type (1) of GC. The presence of a *GNAS*-mutation did not correlate with microsatellite status (Additional file 2: Table S5). No patient with a *GNAS*-mutation harbored a *PIK3CA* (exon 9 and 20)-mutation.

### Immunohistochemical analysis of *GNAS*-expression in gastric cancer

**Fig. 5:**
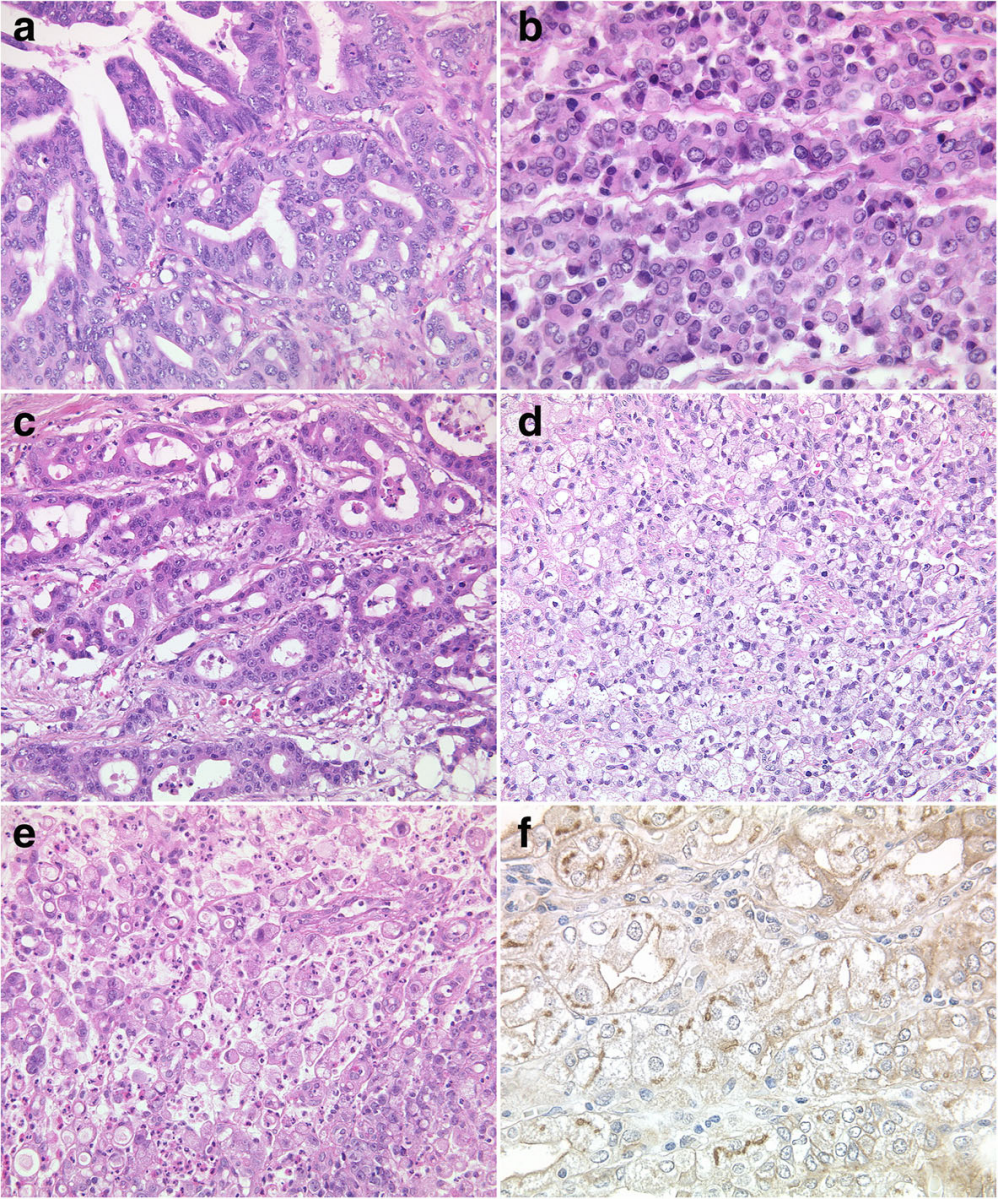
Histology of gastric carcinomas with *GNAS*-mutations (patients No. 3–6 and 9). Seven different gastric cancers with mutations in exon 8 of *GNAS* showed different phenotypes (**a**-**g**). GNAS was detected by immunohistochemistry in 41 gastric cancer specimens and showed membranous and weak cytoplasmic staining. H&E-staining (**a**-**e**); anti-GNAS-immunostaining (**f**). Original magnifications 200×

Next, we studied the expression of *GNAS* by immunohistochemistry using tissue microarrays (Fig. 5). A membranous and/or cytoplasmatic immunostaining of tumor cells was found in 41 patients. The expression of *GNAS* correlated significantly with tumor grade, being more prevalent in well- and moderately differentiated GCs. No correlation was found with the *GNAS*-genotype (wildtype vs. mutated), the microsatellite status or the mucin-phenotype.

## Discussion

Until few years ago, oncologic treatment of cancer largely depended on the anatomic tumor site. However, with the advancements of targeted therapy it became increasingly evident that cancers of the same anatomic origin show great variability in their response rates to chemotherapies necessitating a more in depth phenotypic/genotypic classification. Although this has led to major improvements in lung and colon cancer, it is still in its infancies in GC. Several strategies have been exploited by e.g. the cancer consortia ICGC (International Cancer Consortium) as well as TCGA (The Cancer Genome Atlas) to fill this gap and shed further light on the genetics of GC.

### Selection of applied sequencing and variant detection strategies

This study set out to compare different sequencing tools, both from the technology side (Illumina sequencing by synthesis vs. sequencing by ligation, whole genome vs. whole exome) as well as from the bioinformatics perspective in a small set of two GC samples. The analysis clearly demonstrates the major pitfalls that have to be overcome before such in depth sequencing analysis will become clinical reality. Starting from available clinical material, such analyses will often be associated with problems of DNA quality (e.g. due to tumor necrosis), different sequencing depth and problems of comparability of bioinformatical pipelines. As a relevant example, which has also been extensively described by other groups in other cancer types [19, 20], we here show that the employed SNV-callers led to different results and were characterized by particular strengths and weaknesses in our selected GC cases. We decided to use the caller, which was able to detect the highest amount of variants, for the first round of SNV calls (Samtools or DiBayes) to receive the highest possible number variants, as we had the possibility to validate variants with a second sequencing technology in our study. The union of called variants validated by the other technology were then used for further analyses. Our study also indicates problematic recurrent false positive signals in both tumor samples derived from sequencing-by-ligation whole exome data, a technology that since the inception of the present study has nearly completely vanished from the market. Although we cannot distinguish between the exact amount of problems introduced by the technology (SOLiD) or by the performance of the enrichment, other studies have also reported a higher false-positive rate in WES than in WGS [21]. Also in our hands, several validation attempts for variants exclusively detected in the WES data failed. We show that - given all the limitations of the study - the combination of two sequencing data sets delivers reliable results even in a scenario where only relatively low coverage data is available. In a clinical setting, however, besides precision, also cost effectiveness and duration of analyses play a major role. Thus, ideally WGS as a single technology encompassing the entire variant spectrum has to be applied, which covers actionable recurrent SNVs (e.g. within p53), but also potential structural variants such as gene fusions or amplifications. In reality, even the most sophisticated high-resolution WGS still has a way to go to reach that aim. Until then targeted analyses will clearly have their place in a molecular pathology setting.

How do our results compare to other larger studies? In a comprehensive molecular characterization of 295 primary gastric adenocarcinomas as part of the TCGA project a high mutation frequency in *PIK3CA* (42%) in MSI tumors was reported [22]. Also in the MSI tumor investigated in our study, *PIK3CA* was mutated by a somatic SNV. In addition, TCGA described that *TP53* is often affected in chromosomal unstable tumors, which is also in line with our findings (somatic SNP rs28934574). Furthermore, frequent amplifications of cell cycle mediators (*CCNE1, CCND1* and *CDK6*) were found in the TCGA project [22]. Interestingly, in the MSI tumor sample we detected a somatic large deletion in cyclin *CCND3*, which is also involved in G_1_-S phase progression. Additionally, variants in this gene are significantly associated with survival after diagnosis of breast cancer and with ovarian cancer [23–25]. Recurrent alterations and mutations were also reported in *JAK2* and *ARID1A* in the study of the TCGA [22]. However, these were found in non-hypermutated, MSS and EBV-positive tumor types, respectively, while in our study somatic variants in these genes were detected in the MSS tumor.

Zang et al. [26] carried out exome sequencing on tumor samples of 15 GC patients (11 intestinal, three diffuse and one mixed type according to Laurén). They sequenced ≈8.8 gigabase for each sample and found on average 50 somatic nonsynonymous mutations. Similar to our own cohort, the GC mutation spectrum was dominated by C > T transitions. Zang et al. [26] validated two novel putative tumor suppressor genes (*FAT4* and *ARID1A*). A *FAT4*-mutation occurred in approx. 5% of the GCs. An *ARID1A* gene mutation was present in 8% of the GC patients. Inactivating mutations are more prevalent in MSI GCs. A somatic frameshift indel in *ARID1A* was also found in the MSI tumor sample. ARID1A is involved in chromatin-remodeling and has an antiproliferative effect. Dulak et al. [27] used a different approach. They sought copy number aberrations by high-density genomic profiling in 486 gastrointestinal adenocarcinomas of different anatomical origin. They identified 64 regions of significant recurrent amplification and deletion [27]. Somatic copy-number alterations (SCNA) found in GC included amplification of *EPHB3*, and *GATA4*. Significantly deleted focal SCNAs in GC were, amongst others, in *PDE4D* and *PARK2*. The mentioned genes were also affected by somatic alterations in the investigated MSI tumor sample: *EPHB3* (somatic nonframeshift deletion), *GATA4* (somatic nonframeshift deletion), *PDE4D* (somatic interchromosomal translocation, large deletion), and *PARK2* (somatic large deletion). The genomic variations found by Dulak et al. were common and distinct to various adenocarcinomas of different anatomical origin in the gut. These findings support the cancer association of the somatic large deletions in the genes *PDE4D* and *PARK2* found in the MSI tumor sample. Recently, whole-genome sequencing and comprehensive molecular profiling of 100 tumor-normal pairs of GC identified subtype-specific genetic and epigenetic alterations with unique mutational signatures [28]. Mutated genes reported in this study include, amongst others, *TP53*, *ARID1A*, *IRS2*, and *SUPT3H*, which were all also altered by somatic variants in our study. In addition, Wang et al. suggested a previously unexpected heterogeneity of GC [28], which was also observed in our results.

Thus, even starting from fewer patients compared to the study by Wang et al. [28], few gene mutations merit further attention. Patient No. 2 (MSS tumor) harbored somatic SNVs in *GATA3*, while Dulak et al. [27] found gene amplification of *GATA4* and *GATA6*. Transcription factors of the GATA family are essential regulators of the specification and differentiation of numerous tissues. They all share two highly conserved zinc fingers of the C2H2 type that mediate not only DNA binding but also the great majority of protein interactions [29]. GATA factors coordinate cellular maturation with proliferation arrest and cell survival. *GATA4* and *GATA6* are expressed differentially in normal and neoplastic gastrointestinal mucosa [30]. GATA3 is involved in breast cancer progression and metastasis [29]. So far, GATA3 has not been linked with GC biology.

Patient No. 2 (MSS tumor) also showed a somatic mutation in *ROS1*. ROS1 is one of the 58 different tyrosine kinase receptors encoded in the human genome and evolutionary related to ALK1. ROS1 rearrangement was discovered in glioblastoma, non-small-cell lung cancer, cholangiocarcinoma and also recently in GC [31, 32]. However, ROS1-rearrangement is rare in GC (<5%) [32]. This observation is in line with the findings made by Deng et al. [33]. While, as a group, alterations in genes coding for receptor tyrosine kinases (i.e. *FGFR2*, *EGFR*, *ERBB2, MET* and *ROS1*) are common in GC, alterations of individual members rarely seem to exceed 10%.

Patient No. 1 (MSI tumor) harbored a somatic mutation in *MSH4* lending support to the hypothesis that this gene may be involved in the pathogenesis of MSI of GC. *MSH4* encodes a member of the DNA mismatch repair mutS family, which plays a role in meiotic and mitotic DNA double strand break (DSB) repair and DNA damage responses in human cells [34]. Until now, a link between MSH4-mutation and GC biology is unknown.

Viral replication is usually inhibited before any damage is caused. However, tumor cells have a higher susceptibility to viruses, which might be for example due to defects in the antiviral innate immune response or defects in the p53 pathway. The pathways involved in oncolysis may reflect mechanisms of tumorigenesis and may uncover causes of malignant disease [35]. This is in line with the results of our study, in which an enrichment of variants was observed for genes involved in the negative regulation of the viral reproduction. Furthermore, the GO-term ‘negative regulation of transposition’ was more often affected than expected. Also previous studies have shown a link between transposition and cancer development [36]. Often mobile elements get activated by the cell malignization process, which promotes increased mutation and recombination rates in the genome [37]. Both traits were also observed in the tumor samples of our study. Additionally, an enrichment of variants were observed for the term “DNA cytosine deamination”, which probably caused the increased number of somatic C > T base substitutions in the MSI tumor. The latter was also reported by Greenman et al. [6] and Nagarajan et al. [38]. Furthermore, in both tumor types an increased number of somatic C > A SNVs was observed, which were likely caused by reactive oxygen and nitrogen species (ROS and RNS) [39]. In contrast to the MSI tumor, somatic T > G substitutions occurred with a higher frequency in the MSS tumor of patient 2 than in the controls. This characteristics was also reported by Wang et al. [21] as well as the TCGA consortium [22] and could indicate that this tumor type is caused by another carcinogen or a lower activity of the error-prone polymerase η [40].

Finally, we also found a somatic indel in *GNAS*, which is located on chromosome 20q13.3 and encodes the G-protein alpha subunit. G-proteins are a family of guanine-nucleotide-binding proteins that are important for signal transduction of activated G-protein coupled receptors (GPCR). GPCR-signaling is under tight temporal and spatial control and these receptors exhibit different conformational states, which activate variable intracellular signaling pathways [41]. G-proteins themselves can be activated independently of GPCRs, e.g. by receptor tyrosine kinases or even mutations. 4.2% of all tumor sequences deposited today show activating mutations in *GNAS* [41]. In fact, GNAS is considered to be one of the most frequently mutated G-proteins in human tumors [41]. Particularly hotspot mutations in GNAS (R201 and Q227) disrupt GTPase activity and lead to constitutive activity and persistent signaling. Given the high prevalence of *GNAS*-mutations in human tumors, we further explored *GNAS* by pyrosequencing in a large GC patient cohort and found hotspot-mutations only in 1.4% of the patients (see Additional file 4: Supplementary Materials and Methods). Thus, hotspot-mutations of *GNAS* are infrequent in GC. However, a male preponderance was interesting to note. Recently, it was suggested that *GNAS*-mutations are common in pyloric gland adenomas of the stomach and duodenal mucosa [42] and may thus also specify a particular phenotype of GC. However, the *GNAS*-mutant GCs of our cohort showed no specific phenotype. More interestingly, immunodetection also did not correlate with phenotype or even the presence of the gene-mutation and thus cannot serve as screening tool.

## Conclusions

We show here in a multiple-tool comparative approach in clinical samples that different NGS approaches will identify a large variety and number of genetic alterations in GC. Validation studies usually provide prevalences ranging from 1 to 15%. Our results are in line with these recent findings and present a benchmark strategy of individual tumor genome analysis combining both, WES and WGS information with two different NGS platforms, used population-based whole genome resources as a novel pathway-based filter, and integrated SNV as well as structural variant analyses. Using this comprehensive strategy we identified a multitude of novel somatic potentially damaging mutations and show that MSS and MSI GCs have markedly different numbers of somatic and germline mutations, which is in line with observations made by Wang et al. [28]. This further underlines a specific hallmark of GC, i.e. the large variety of different genetic alterations leading to significant tumor heterogeneity. Importantly, our data also point to distinct mutational processes, which are responsible for the different mutational landscapes of the individual tumors. It is thus tempting to speculate that the identification of such signatures by cancer genome sequencing could reflect a potential future biomarker for therapy stratification. Compared with colorectal cancer, no characteristic “carcinogenesis-pathway” has been discovered for GC, as yet. Our study clearly underlines the value of individual genome sequencing to depict the multidimensional aberrations in GC, but also demonstrates challenges (e.g. functional annotation in the light of high genomic diversity of the cancer type, sample heterogeneity and clinically reliable, quick turn round times) must be solved before genome-driven therapy stratification will become clinical reality.

## Methods

Full details about the applied methods are provided in the Additional file 4: Supplementary Materials and Methods. Furthermore, an overview of the applied workflow is provided in Figs. 1 and 2.

### Tissue samples

From the archive of the Institute of Pathology, University Hospital Kiel, we retrieved tissue samples from two female patients who had died from an intestinal type of GC, which differed by their microsatellite status (see Additional files 3 and 4). Samples from the primary tumor and corresponding non-neoplastic gastric mucosa had been collected immediately after surgery, were fresh frozen in liquid nitrogen and stored at −80 °C until use. Genomic DNA was extracted after tissue homogenization with the QIAamp DNA mini kit (Qiagen, Hilden, Germany) following the manufacturer’s instructions. The patients had given written informed consent to prospective tissue sampling of excess tissue material, which was no longer needed for diagnostic or therapeutic purposes. The project was approved by the local ethics committee of the University Hospital in Kiel, Germany (reference numbers AZ 140/99 and D 453/10). All patient data were pseudonymized before study inclusion.

### Library preparation and sequencing

Enrichment of the whole exome was performed with the Agilent Sure Select Target Enrichment Human All Exon v2 kit. After the ePCR with the Solid PCR Kit sequencing was done with a Solid paired 50/35 v4 run. Libraries for the whole genome sequencing were created with the Illumina TruSeq DNA sample prep kit. After cluster generation on the cBot with the TruSeq PE Cluster Kit (v2.5 for samples of patient 1 (microsatellite unstable (MSI) tumor (TU) + non-tumor (NT)) and patient 2 (microsatellite stable (MSS) TU) and v3 for the NT-sample the sequencing was performed with the TruSeq SBS Kit (200 cycles, paired end modus) on the Illumina HiSeq 2000. SNVs in the genes *DROSHA*, *MSH4*, *RERE*, *ROS1*, *TACC2*, and *TYRO3* were additionally verified by pyrosequencing on a Pyromark Q24 device (QIAGEN). Primers are shown in Additional file 2: Table S1. Furthermore, the hotspot mutation in *GNAS* was investigated using the same technology in a large cohort of 482 GC patients (Table 1).

### Bioinformatics analysis

#### Mapping

Illumina WGS (whole genome sequencing) sequences were mapped against the human genome reference hg19 with BWA v0.5.9 [43], while the Solid WES (whole exome sequencing) reads were aligned with Bioscope v1.2.1 (Applied Biosystems™). Reads with the same starting point defined as duplicates were marked with the Picard tool MarkDuplicates.jar v1.41 (http://broadinstitute.github.io/picard/) and removed from further analyses.

Please note that there are differences in the coverage distribution, which we handled with adapted filter steps. This might be also necessary in clinical samples.

#### SNVs and small indels

SNV-calling was performed with GATK v1.3 [17], DiBayes (bioscope v1.2.1), and Samtools v0.1.16 [44] in the WES and with Samtools as well as GATK in the WGS data. Small indels were called with DiBayes in the WES and Samtools in the WGS mappings. A detailed description of the filter pipeline, the applied parameters as well as a comparison between the different technologies and SNV caller are described in the Additional file 4: Supplementary Materials and Methods. Annovar [45] (version Jun 2011) was used for the annotation of SNVs and small indels. SNVs, which were not annotated in dbSNP build 132, were classified as novel. The databases OMIM (Online Mendelian Inheritance in Man, McKusick-Nathans Institute of Genetic Medicine, Johns Hopkins University (Baltimore, MD), http://omim.org/), HGMD (Human Gene Mutation Database) [46] and GWAS (Genome-wide association studies) were checked for known cancer associated SNVs. Signatures of the SNV patterns were investigated with the R package SomaticSignatures [47]. The influence of signatures annotated in COSMIC to the observed mutational patterns were calculated with the R package deconstructSigs [13].

#### Definition of the exonic gene conservation score

We defined an additional indicator for potentially clinically relevant exonic small variants: The exonic gene conservation score (ECS) was calculated for the exonic regions of each gene using all coding non-synonymous variants (germline and somatic) of 1092 samples from the 1000 Genomes project. Therefore, all variants were annotated with the program Annovar [45]. The ECS was defined as the mutation rate within the exonic regions of the gene divided by the average mutation rate over all exonic regions:

$$ \frac{<\#\mathrm{variants}\ \mathrm{in}\ \mathrm{gene}>{}^{\ast }<\mathrm{total}\ \mathrm{length}\ \mathrm{of}\ \mathrm{exonic}\ \mathrm{regions}>}{<\mathrm{length}\ \mathrm{of}\ \mathrm{exonic}\ \mathrm{regions}\ \mathrm{of}\ \mathrm{the}\ \mathrm{gene}>{}^{\ast }<\mathrm{avg}.\ \mathrm{total}\#\mathrm{exonic}\ \mathrm{variants}>{}^{\ast }<\#\mathrm{samples}>} $$

Thus, the smaller the ECS, the higher is the conservation of the exonic region of the gene. Genes with ECS < 0.01 were defined as conserved.

#### Large structural variations

Large deletions, inversions, tandem duplications, and interchromosomal translocations were called with Breakdancer (v1.2) [48]. Sensitivity and specificity of the first three structural variant types listed above were increased by using Pindel (v0.2.3) [49]. In order to find large insertions all unmapped reads with a quality score larger than 20 at 80 or more positions were assembled with velvet v1.2.06 [50] in the tumor samples. The resulting contigs were aligned with BLAT v.34 to the human reference to find putative insert sites. All unmapped reads of the corresponding control samples were mapped against the contigs of the tumor sample. Contigs, which were covered at 90% or more positions, were defined as germline variant and therefore excluded from the analysis. All structural variants were filtered with help of split reads and the paired end information. To identify split reads with parts mapping to different chromosomes or with a large gap between them, all unmapped reads with a quality score greater or equal than 35 at 95 or more positions were aligned against the human reference using the program BLAT. Exclusively reads with maximum two alignment parts and a sum of at least 92 mapped bases were considered for further analyses. The applied filter parameter are described more detailed in the Additional file 4: supplementary methods. The algorithm for the detection of large insertion is displayed in Additional file 1: Figure S14. The visualization of the large structural variations was performed with Circos v0.62 [51].

#### Pathway analysis

For pathway analysis, germline SNVs of 1092 samples out of the 1000 Genomes project were annotated with Annovar v7. For each pathway a statistic was created with the number of affected genes as well as the number of SNVs. Only SNVs, which were predicted to be damaging to protein function, were used. Next, the number of affected genes/SNVs per pathway was compared between the tumor samples and the maximum count in the samples of the 1000 Genomes project. To ensure comparability between the samples investigated in this study and the 1000 Genomes project, exclusively SNVs called with GATK were used for that study. The analysis was performed for all terms annotated in GO [52].

#### Test for bacterial or viral infection

To test for bacterial or viral infection, from each tumor sample over 20,000 unmapped reads with more than 95 bases having a quality score larger 35 were blasted against the NCBI nucleotide collection database nt. Furthermore, all reads with a quality score larger than 20 at 80 or more positions were assembled with velvet v1.2.06 and the resulting contigs blasted against nt.

## Additional files

Additional file 1: Figure S1- Figure S20. (PDF 2.74 mb)

Additional file 2: Table S1- Table S9. (PDF 364 kb)

Additional file 3: Supplemental results [52] (Additional file 1: Figure S1, Fig. 2, Additional file 1: Figure S15, Fig. 2, Additional file 1: Figure S15, Additional file 1: Figure S16, Additional file 1: Figure S17, Additional file 1: Figure S18, Additional file 1: Figure S19, Additional file 4: Methods, Additional file 1: Figure S20, Additional file 1: Figure S20 A + B, Additional file 1: Figure S20 C + D, Additional file 1: Figure S12 and Additional file 1: Figure S13). (DOCX 30 kb)

Additional file 4: Supplemental Materials and methods [18, 53–59] (Table 1, Additional file 2: Table S2C-F and S3, Additional file 2: Tables S3A and S3B). (DOCX 35 kb)

## Abbreviations

cPI: Cancer proliferation index
EBV: Epstein-Barr-virus
ECS: Exonic conservation score
GC: Gastric cancer
GPCR: G-protein coupled receptors
indel: Insertion/deletion
MSI: Microsatellite instable
MSS: Microsatellite stable
NGS: Next generation sequencing
RNS: Reactive nitrogen species
ROS: Reactive oxygen species
SCNA: Somatic copy number alteration
SNV: Single nucleotide variation
TCGA: The Cancer Genome Atlas
WES: Whole exome sequencing
WGS: Whole genome sequencing
