# Supplementary material for: Interpreting whole genome and exome sequencing data of individual gastric cancer samples
Source: BMC Genomics. 2017 Jul 6;18:517. doi: 10.1186/s12864-017-3895-z (PMC5501078; doi:10.1186/s12864-017-3895-z)
Supplement: Supplementary file 1 — Figure S1- Figure S20. (PDF 2.74 mb) [file 12864_2017_3895_MOESM1_ESM.pdf]

Additional File 1

Interpreting whole genome and exome sequencing data of individual gastric cancer samples

Daniela Esser[1,4], Niklas Holze[2], Jochen Haag[2], Stefan Schreiber[1,3], Sandra Krüger[2], Viktoria Warneke[2], Philip Rosenstiel[1†] and Christoph Röcken[2*†]

[1] Institute for Clinical Molecular Biology, Christian-Albrechts-University, 24105 Kiel, Germany
[2] Institute for Pathology, Christian-Albrechts-University, 24105 Kiel, Germany
[3] Department of General Internal Medicine, University Hospital Schleswig-Holstein, 24105 Kiel, Germany
[4] Institute for Experimental Medicine, Christian-Albrechts-University, 24105 Kiel, Germany

Figure S1

Coverage distribution from (A) WGS and (B) WES (target region). Number of called SNVs in each sample using different variant caller (C).

Figure S2

Estimation of sample purity. Peak shows the mutant allele fractions for heterozygous SNVs. This results in a tumor ratio, which is twice as high as the peak.

Figure S3

Comparison between SNVs called in WES data and SNVs called in WGS data. Note that in this case, “somatic” refers to SNVs, which were not detected in the corresponding non-tumor samples sequenced with the same technology.

Figure S4

Ratio of SNVs, which were called in one sequencing data set and could be validated in the second. (A) Total number of SNVs. (B) Exclusively SNVs, which were covered in both data sets. Note that in this case, “somatic” refers to SNVs, which were not detected in the corresponding non-tumor samples sequenced with the same technology.

Figure S5

Rainfall plots based on all somatic SNVs. The x-axis shows the chromosomal position. The distance between each mutation and the prior variant is plotted on the y-axis. The colors of the dots indicate the SNV-type.

Figure S6

Somatic SNV signatures estimated with NMF (non-negative matrix factorization).

Figure S7

(A) Description of SNV signatures identified in the investigated tumor samples. (B) Contribution of the identified signatures to the investigated samples.

Figure S8

Somatic SNV spectrum of TCGA WES samples and investigated GC tumor samples. Somatic signatures were estimated with NMS.

Figure S9

Number of somatic SNVs and somatic indels after different steps.

Figure S10

Protein network for MSI (A) and MSS (B) tumors based on all somatic SNVs, which were a stopgain mutation, predicted as damaging, at a conserved position or in a conserved gene. The connections were based on medium stringency according to the STRING database. Proteins without any connection are not displayed.

Figure S11

Enriched processes of the two protein subnetworks identified in the first patient. (A) Larger network. (B) Smaller network.

Figure S12

Enriched GO terms in MSS tumor. GO terms, which were more often affected in the tumor sample than in all individuals of the 1000 Genomes project, were included in the figure. To enable comparability between the samples, all called variants (germline + somatic) were used. (A) Analysis based on number of genes affected by at least one SNV with damaging prediction. (B) Analysis based on number of SNVs predicted as damaging.

Figure S13

Enriched GO terms in MSI tumor. GO terms, which were more often affected in the tumor sample than in all individuals of the 1000 Genomes project, were included in the figure. To enable comparability between the samples, all called variants (germline + somatic) were used. (A) Analysis based on number of genes affected by at least one SNV with damaging prediction. (B) Analysis based on number of SNVs predicted as damaging.

Figure S14

Algorithm to detect and filter large insertions

Figure S15

Overlap of SNVs called by different programs

Figure S16

The figure shows the number of verified SNVs for each SNV caller. Exclusively positions covered in both data sets were included in the analysis. In the exome data are from outside to the inside the results of the SNV callers GATK, Samtools, and DiBayes. In the genome data the results of GATK are displayed outside and the one of Samtools inside. The percentage of SNVs, which have less than 20% read support in the other data set are marked in light, the remaining ones in dark grey.

Figure S17

The figures show for each SNV-Caller the percentage of called SNVs out of all cross platform variants. (A) WGS and (B) WES. The SNVs were called confirmed, if in the data of the second technology (i) 10%, (ii) 20% or (iii) 50% of the reads support the variant. (C) Table summarizing the confirmed SNV counts.

Figure S18

Comparison between strict filtered (detected with all callers) SNVs in the WES data (called with Samtools and GATK and DiBayes) and those detected in the WGS data (called with Samtools and GATK).

Figure S19

False positive and false negative rate in WES and WGS sequencing data. All exonic SNVs called with Samtools were investigated in all sample pairs and compared between WES and WGS. (A) Percentage of SNVs, which were called in WGS and either uncovered or not supported in the WES data. (B) SNV type distribution for three classed of SNVs called in WGS: all SNVs called in WGS data, SNVs uncovered in WES, SNVs covered but not supported in WES. (C) Percentage of SNVs, which were called in WES and either uncovered or not supported in the WGS data. (D) SNV type distribution for three classed of SNVs called in WES: all SNVs called in WES data, SNVs uncovered in WGS, SNVs covered but not supported in WGS

Figure S20

Relation between the novel developed exonic gene conservation score and known cancer associations: (A) Scatter plot comparing the exonic gene conservation score with the cancer proliferation index, whereby tumor suppressors exhibit significant negative cPI values and oncogenes positive cPI values. (B) Enlargement of the small value region of figure part A. (C) Scatter plot comparing the exonic gene conservation score with the number of samples having a mutation with a FATHMM cancer prediction in the COSMIC database. (D) Enlargement of the small value region of figure part C.

Figure S1

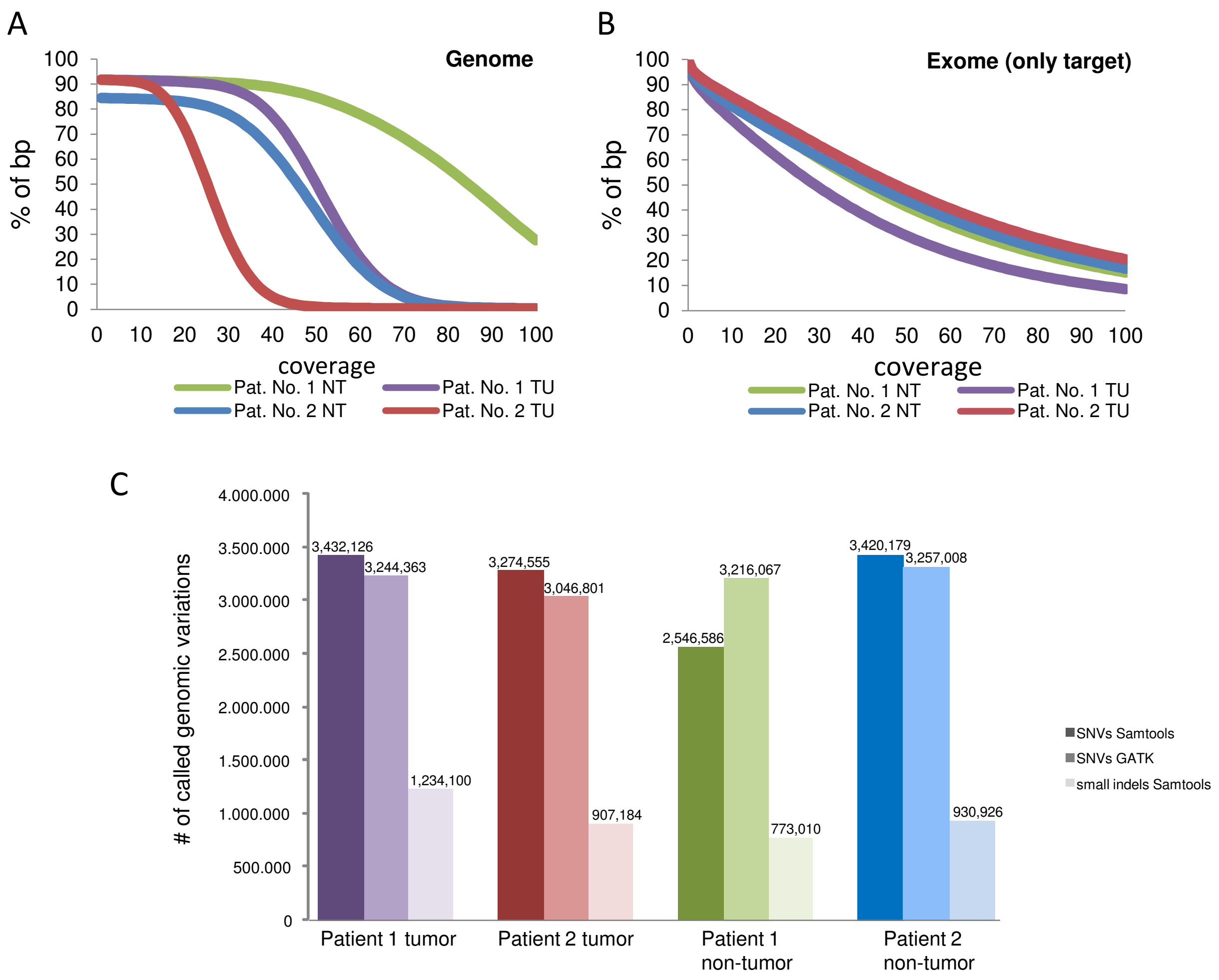
A
Genome
% of bp
coverage
Pat. No. 1 NT
Pat. No. 1 TU
Pat. No. 2 NT
Pat. No. 2 TU
B
Exome (only target)
% of bp
coverage
Pat. No. 1 NT
Pat. No. 1 TU
Pat. No. 2 NT
Pat. No. 2 TU
C
# of called genomic variations
3,432,126
3,244,363
1,234,100
3,274,555
3,046,801
907,184
2,546,586
3,216,067
773,010
3,420,179
3,257,008
930,926
Patient 1 tumor
Patient 2 tumor
Patient 1 non-tumor
Patient 2 non-tumor
SNVs Samtools
SNVs GATK
small indels Samtools

Figure S2

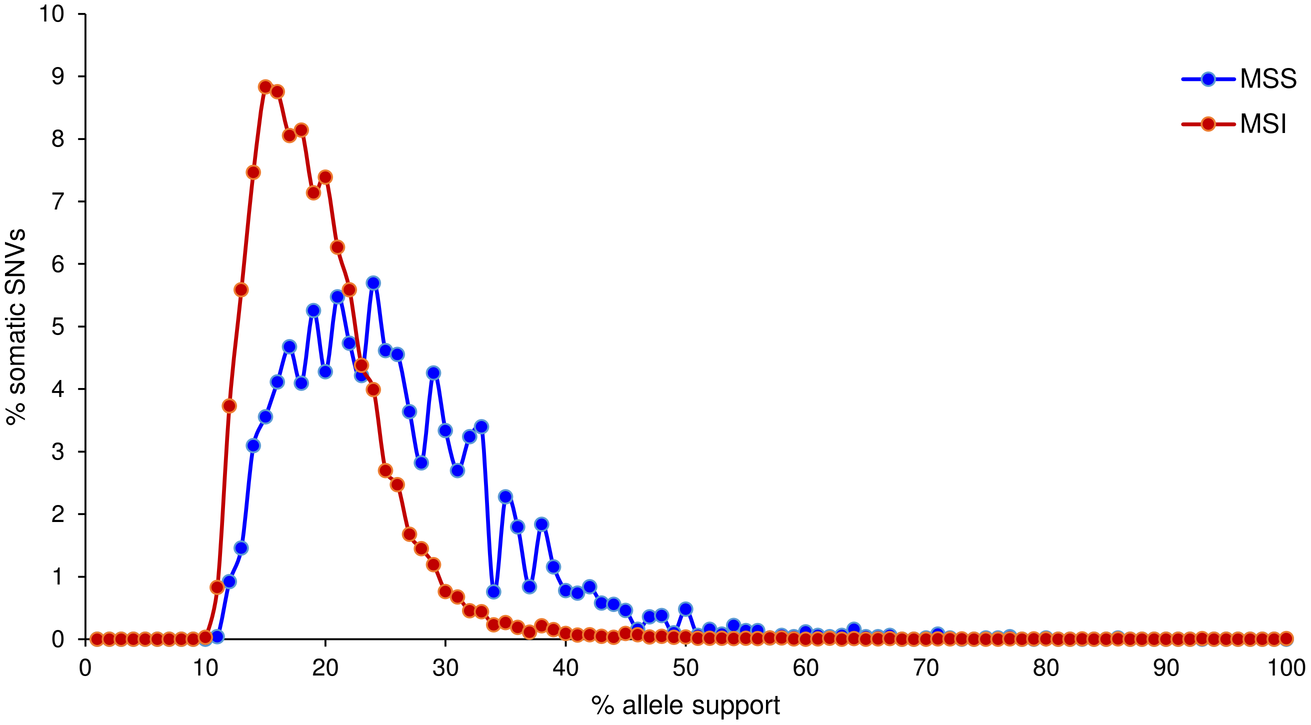

% somatic SNVs
0
1
2
3
4
5
6
7
8
9
10
0
10
20
30
40
50
60
70
80
90
100
% allele support
MSS
MSI

Figure S3

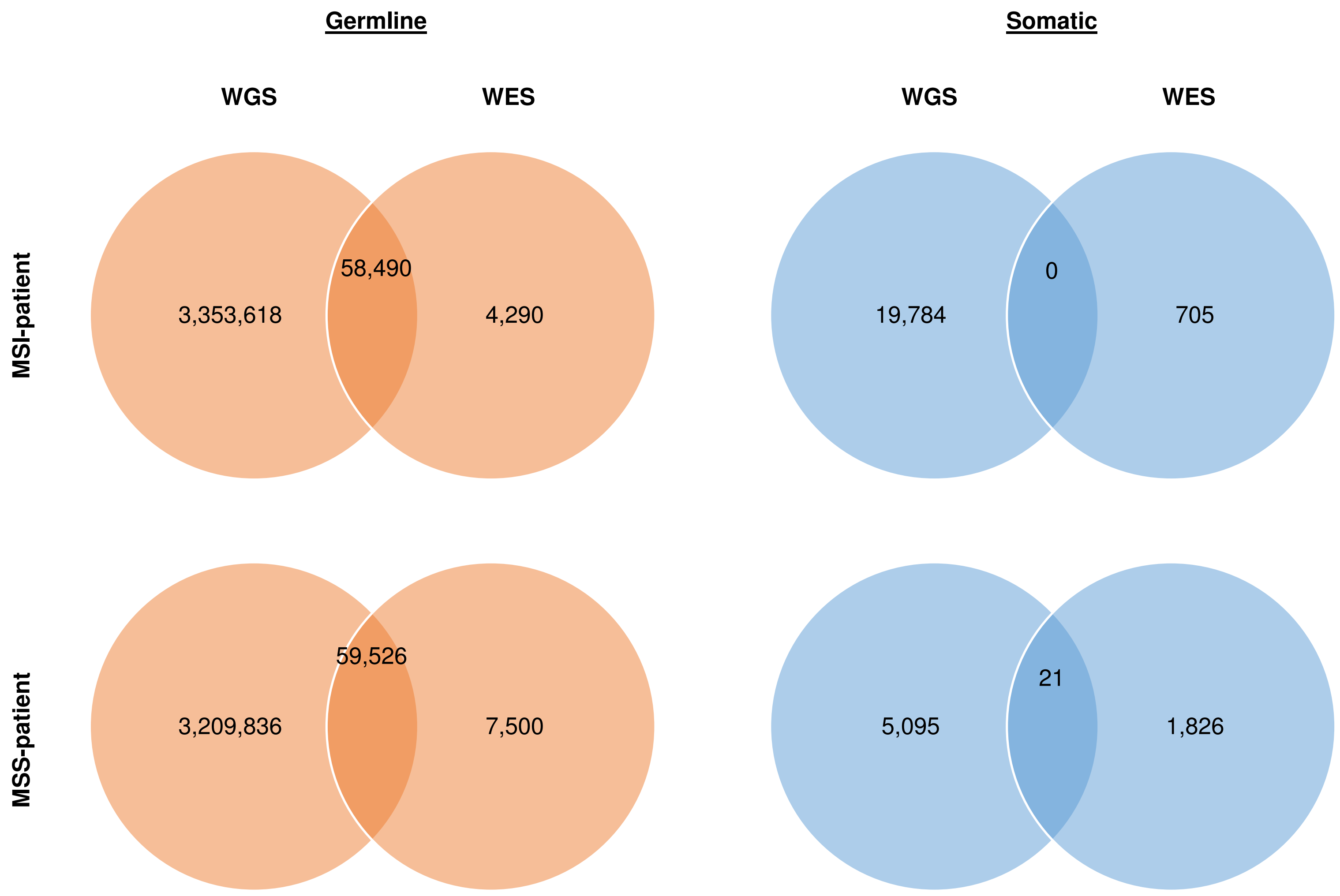
Germline
Somatic
WGS
WES
WGS
WES
MSI-patient
3,353,618
58,490
4,290
19,784
0
705
MSS-patient
3,209,836
59,526
7,500
5,095
21
1,826

Figure S4

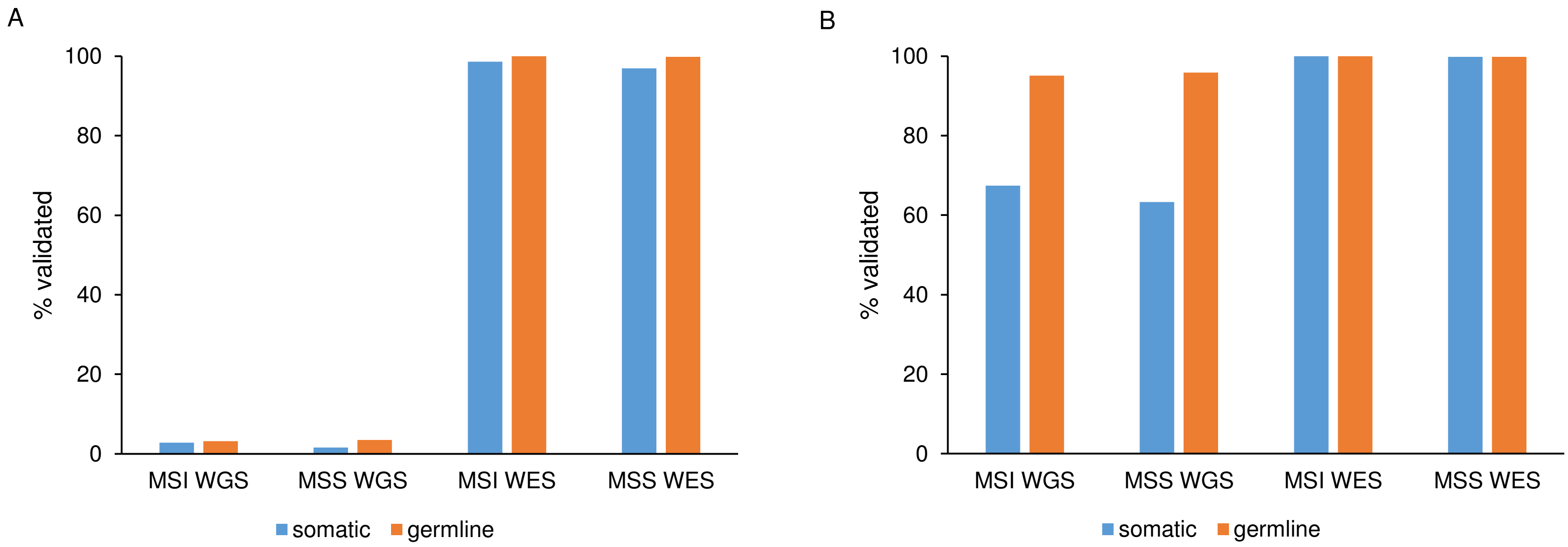
A
B
100
80
60
40
20
0
% validated
MSI WGS
MSS WGS
MSI WES
MSS WES
somatic
germline

Figure S5A

MSS tumor

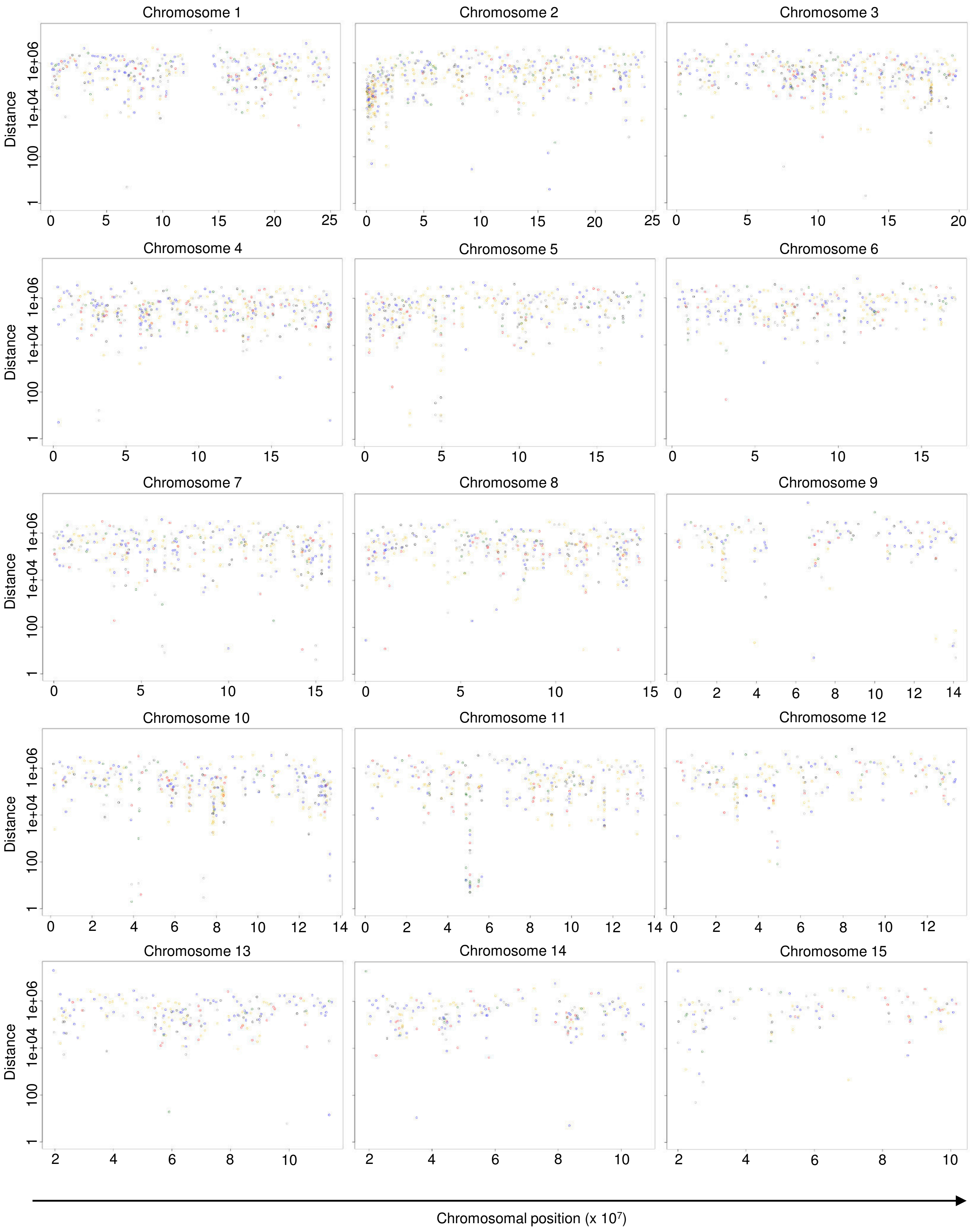

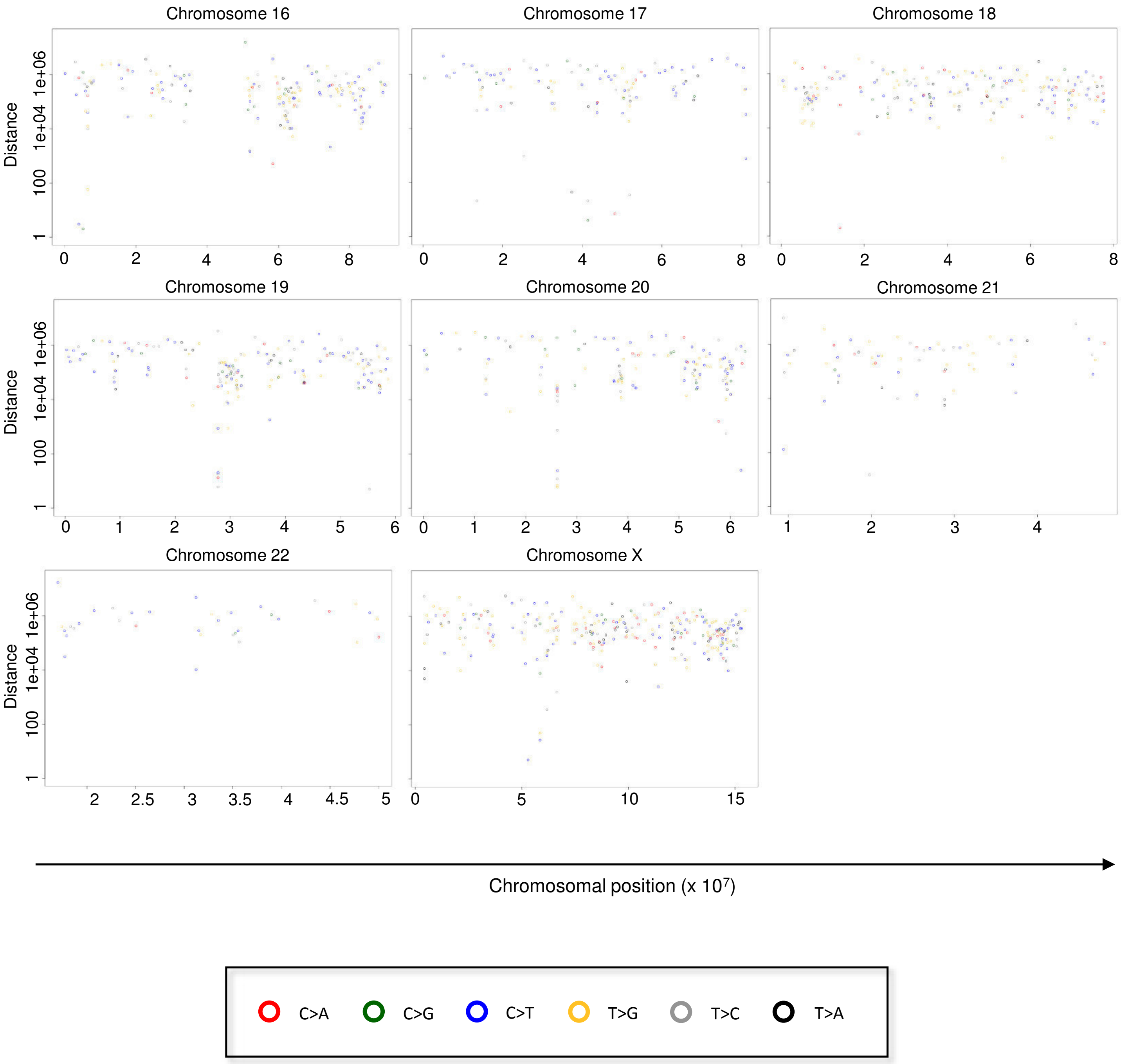
Chromosome 16
Chromosome 17
Chromosome 18
Chromosome 19
Chromosome 20
Chromosome 21
Chromosome 22
Chromosome X
Distance
1e+06
1e+04
100
1
Chromosomal position (x 10^7)
C>A
C>G
C>T
T>G
T>C
T>A

Figure S5B

MSI tumor

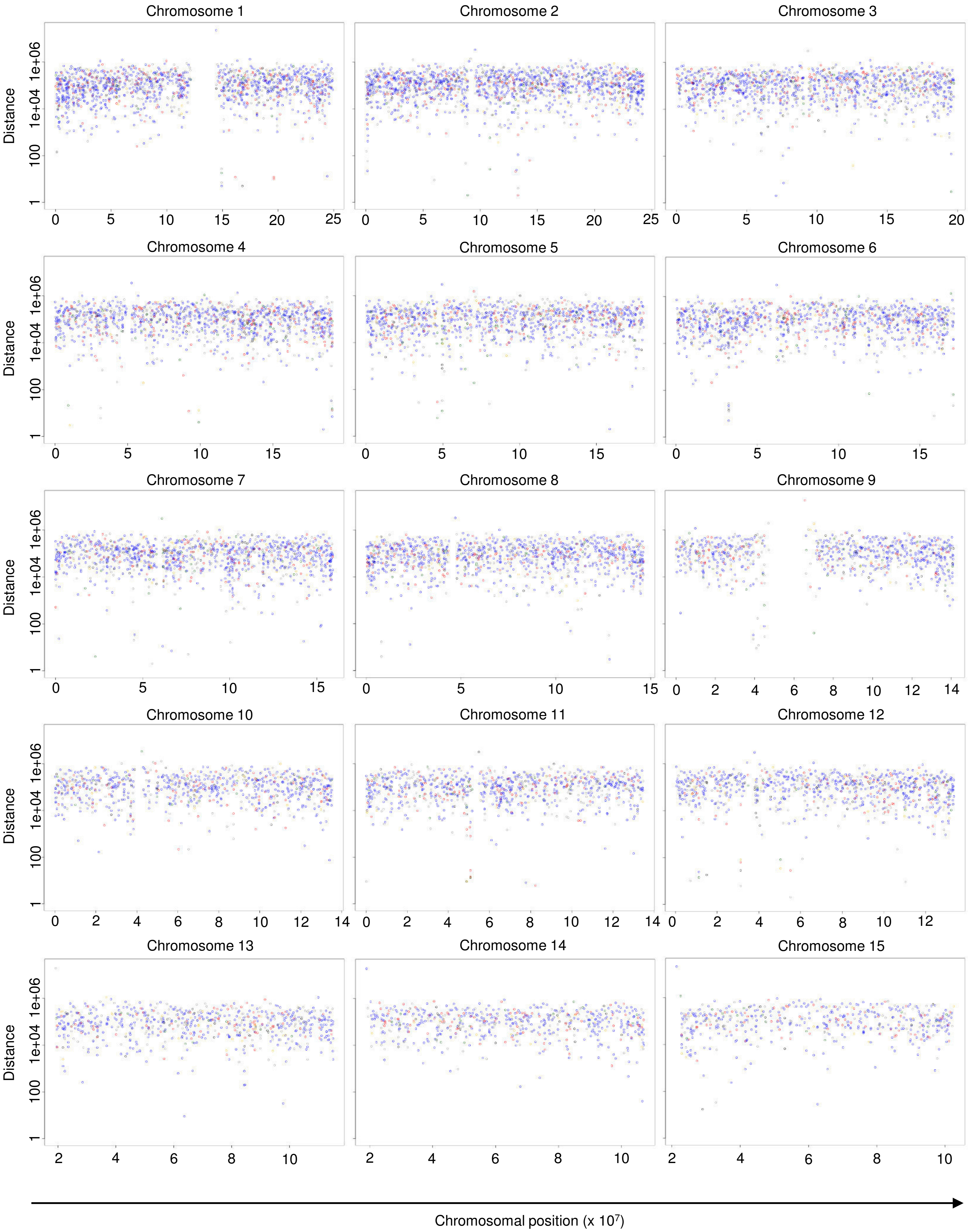

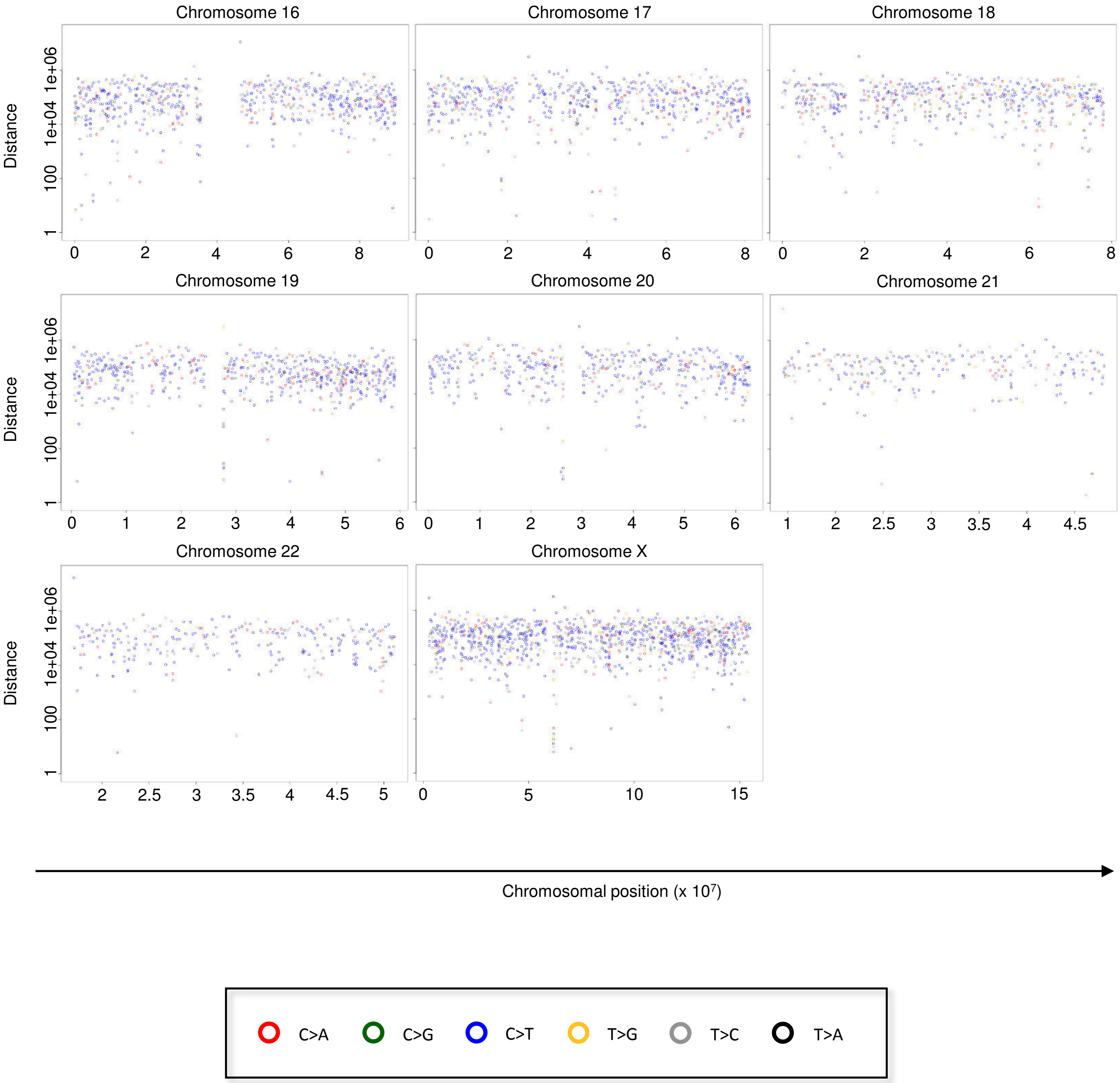
Chromosome 16
Chromosome 17
Chromosome 18
Chromosome 19
Chromosome 20
Chromosome 21
Chromosome 22
Chromosome X
Distance
1
100
1e+04
1e+06
Chromosomal position (x 10^7)
C>A
C>G
C>T
T>G
T>C
T>A

Figure S6

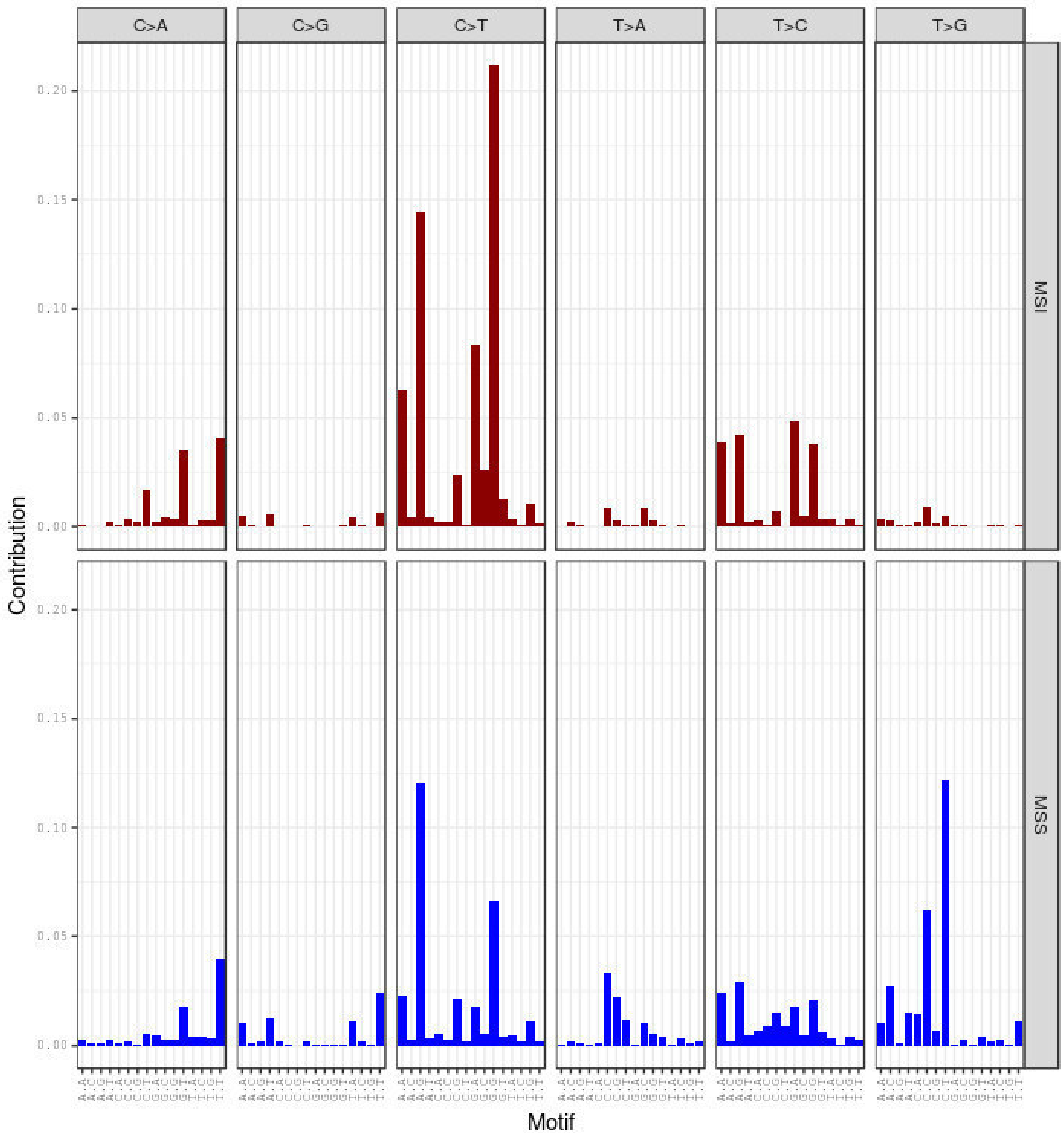

C>A
C>G
C>T
T>A
T>C
T>G
MSI
MSS
Contribution
0.20
0.15
0.10
0.05
0.00
Motif

Figure S7

A

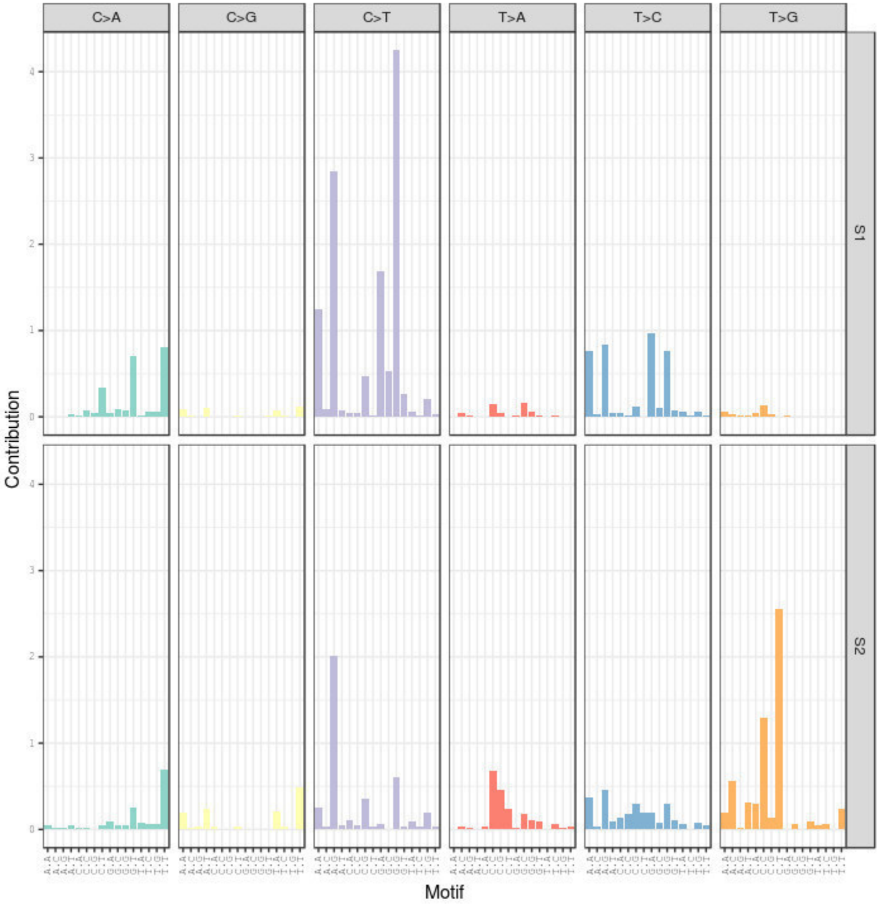
C>A
C>G
C>T
T>A
T>C
T>G
S1
S2
Contribution
Motif

B

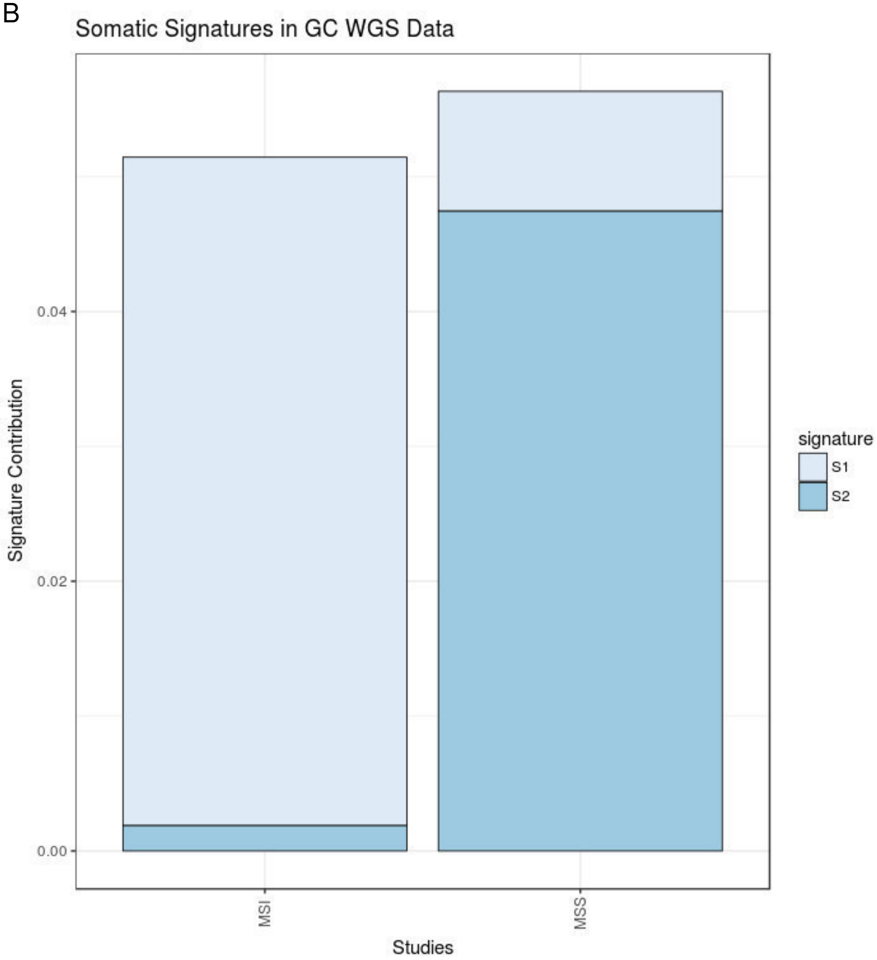
Somatic Signatures in GC WGS Data
Signature Contribution
0.04
0.02
0.00
MSI
MSS
Studies
signature
S1
S2

Figure S8A

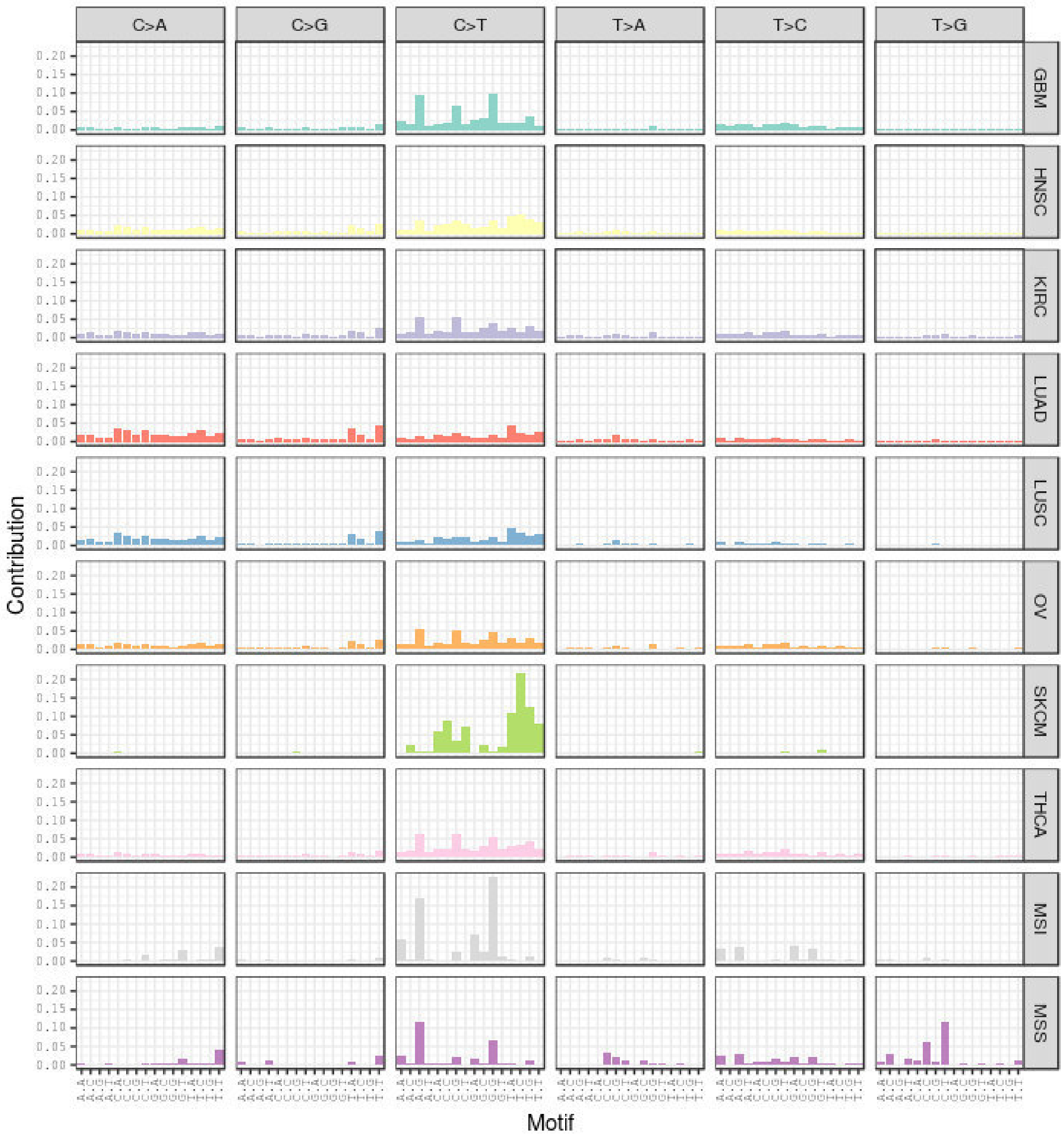
C>A
C>G
C>T
T>A
T>C
T>G
GBM
HNSC
KIRC
LUAD
LUSC
OV
SKCM
THCA
MSI
MSS
0.20
0.15
0.10
0.05
0.00
Contribution
Motif

Figure S8B

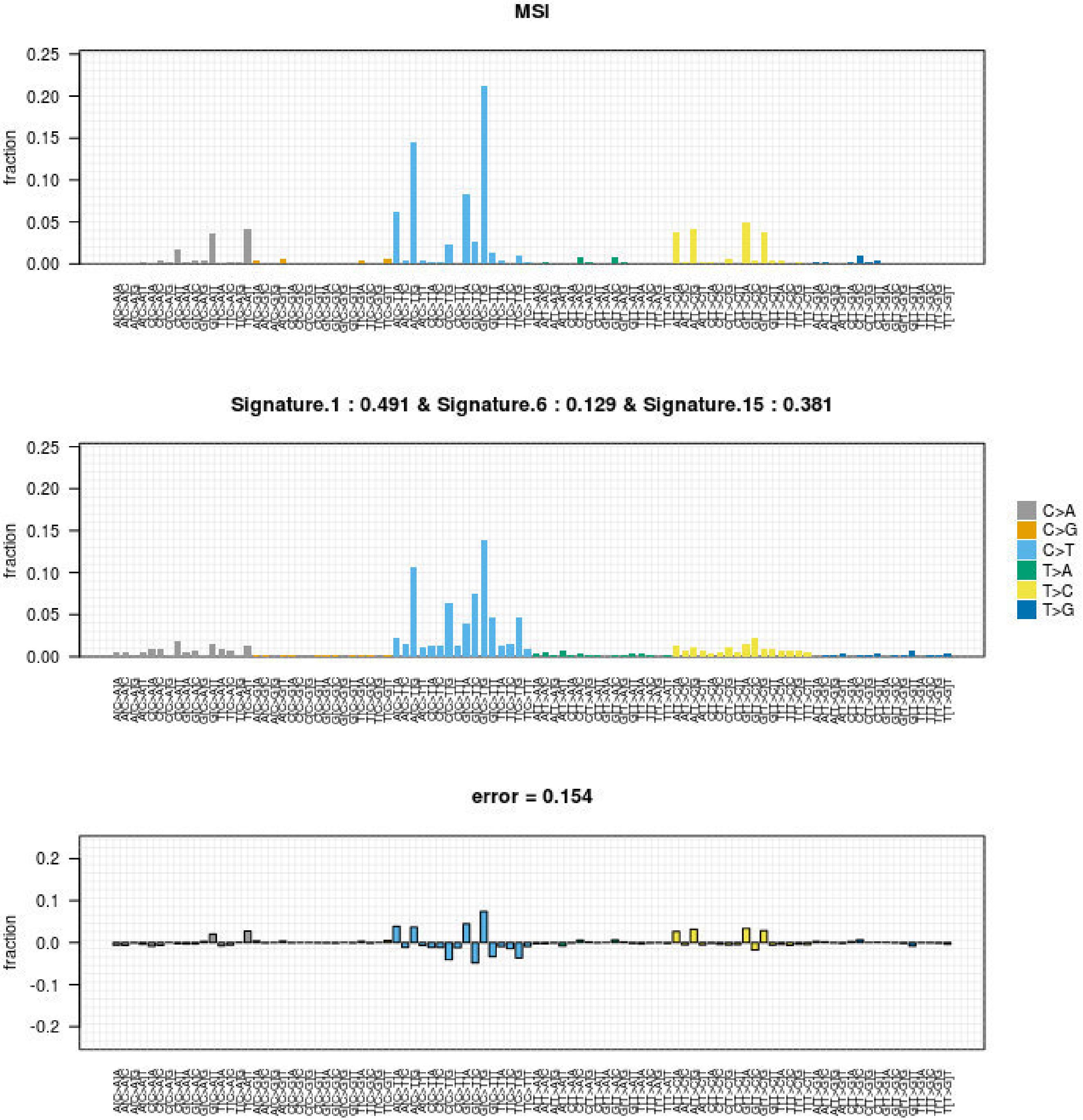
MSI
fraction
Signature.1 : 0.491 & Signature.6 : 0.129 & Signature.15 : 0.381
fraction
error = 0.154
fraction
C>A
C>G
C>T
T>A
T>C
T>G

Figure S8B

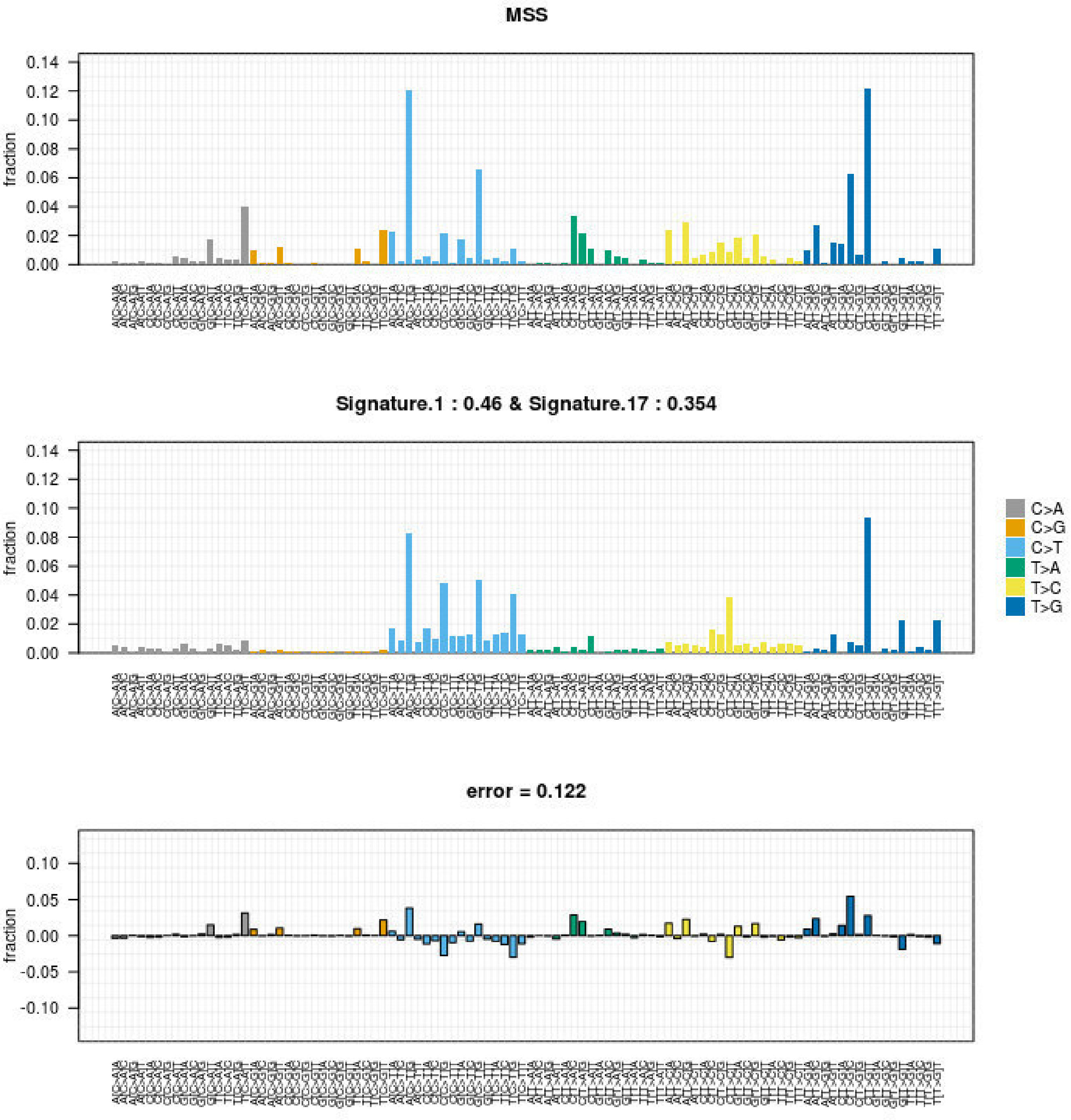
MSS
Signature.1 : 0.46 & Signature.17 : 0.354
error = 0.122
fraction
C>A
C>G
C>T
T>A
T>C
T>G

Figure S9

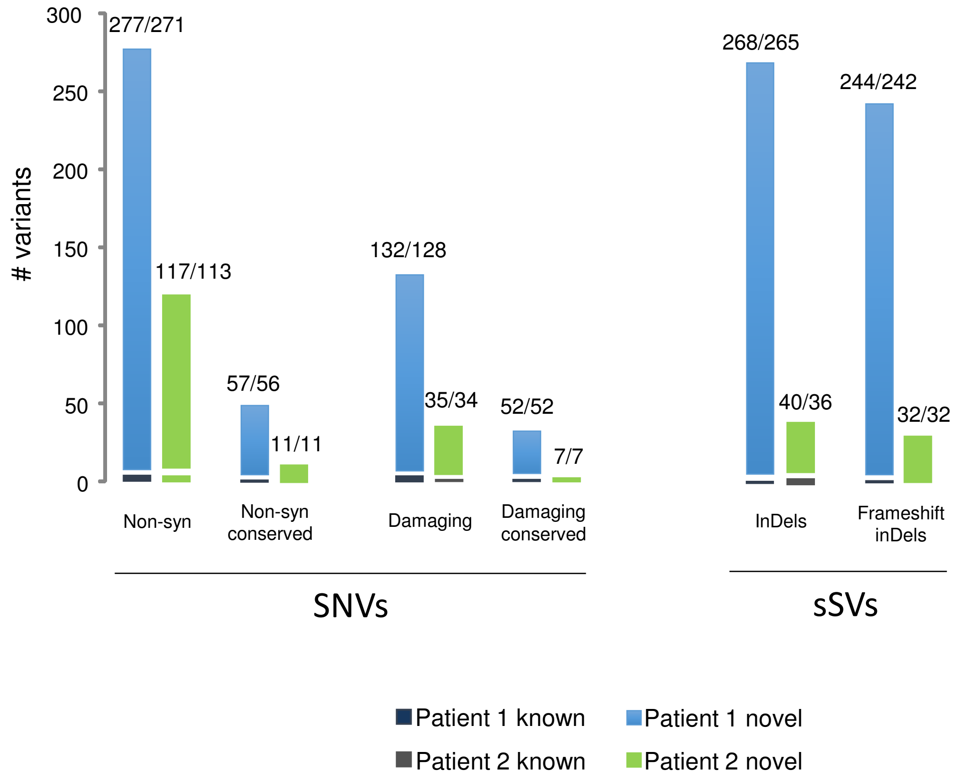

variants
300
250
200
150
100
50
0
277/271
117/113
57/56
11/11
132/128
35/34
52/52
7/7
268/265
40/36
244/242
32/32
Non-syn
Non-syn conserved
Damaging
Damaging conserved
InDels
Frameshift inDels
SNVs
sSVs
Patient 1 known
Patient 1 novel
Patient 2 known
Patient 2 novel

Figure S10 A

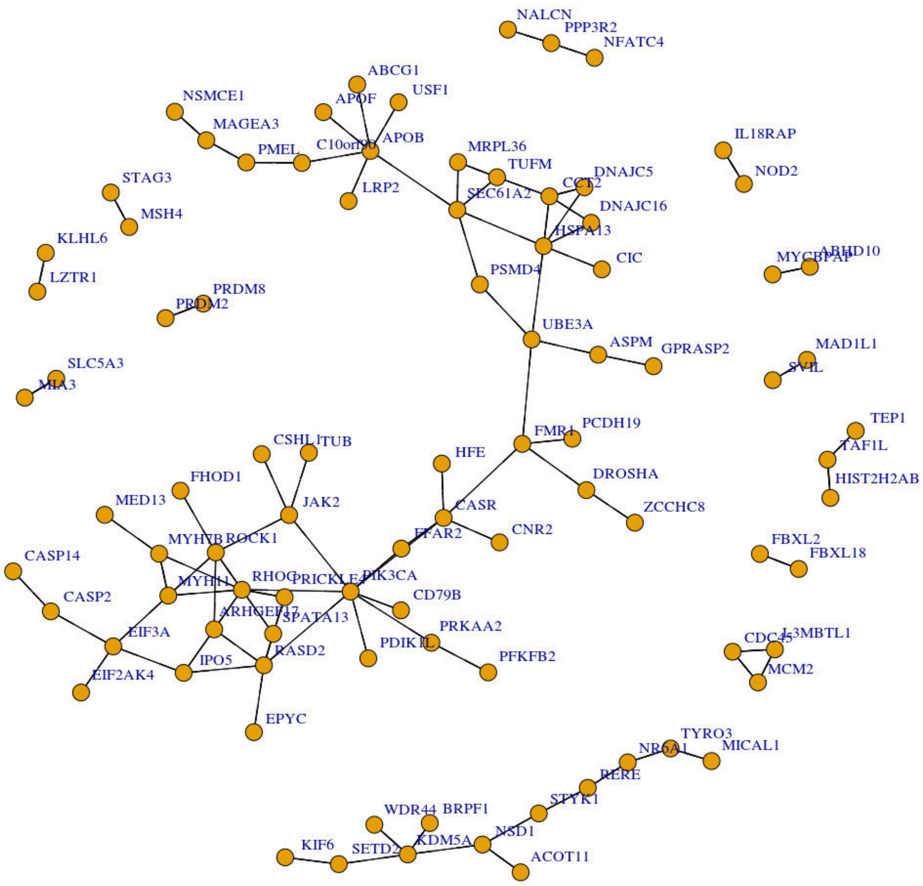
NALCN
PPP3R2
NFATC4
ABCG1
APOF
USF1
NSMCE1
MAGEA3
PMEL
APOB
MRPL36
TUFM
DNAJC5
CCT2
SEC61A2
DNAJC16
HSPA13
CIC
PSMD4
IL18RAP
NOD2
STAG3
MSH4
KLHL6
LZTR1
LRP2
PRDM8
PRDM2
MYCBPAP
ABHD10
UBE3A
ASPM
GPRASP2
MAD1L1
SVIL
SLC5A3
MIA3
TEP1
TAF1L
HIST2H2AB
FMR1
PCDH19
HFE
DROSHA
ZCCHC8
CSHL1
TUB
FHOD1
MED13
JAK2
CASR
CNR2
FFAR2
MYH7B
ROCK1
CASP14
CASP2
RHOC
MYH11
PIK3CA
CD79B
ARHGEF17
SPATA13
EIF3A
PRKAA2
PDIK1L
PFKFB2
IPO5
RASD2
EIF2AK4
EPYC
FBXL2
FBXL18
CDC45
L3MBTL1
MCM2
TYRO3
MICAL1
NR6A1
RERE
STYK1
WDR44
BRPF1
NSD1
KIF6
SETD2
KDM5A
ACOT11

Figure S10 B

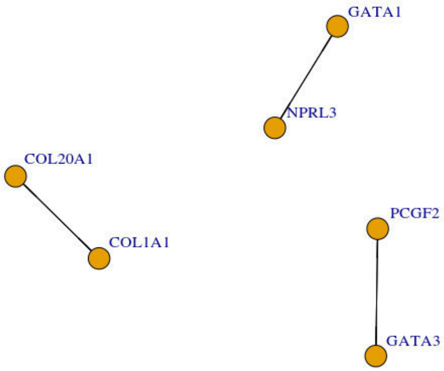

Figure S11

A

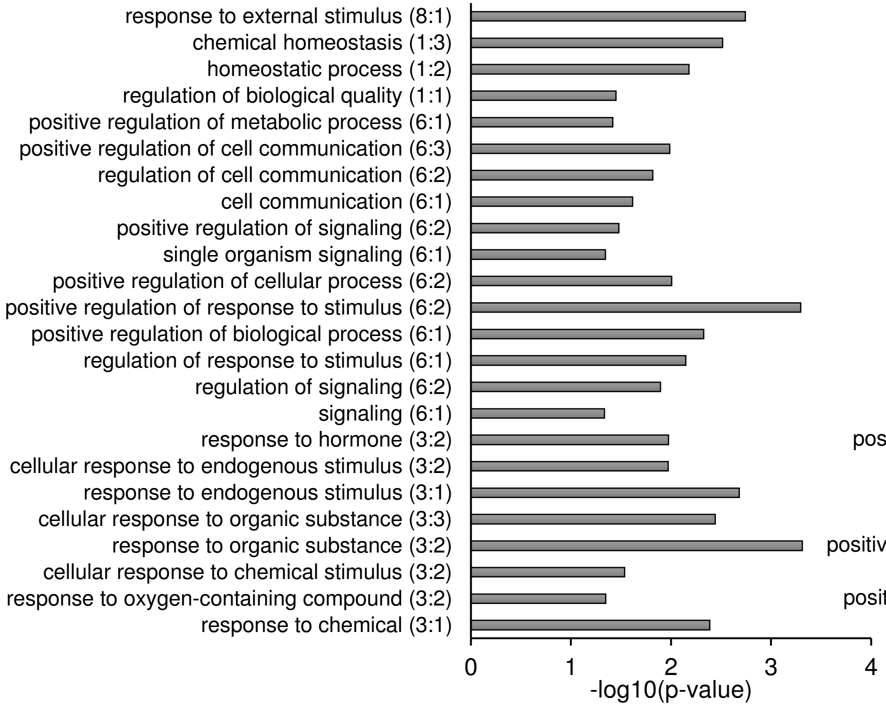

B

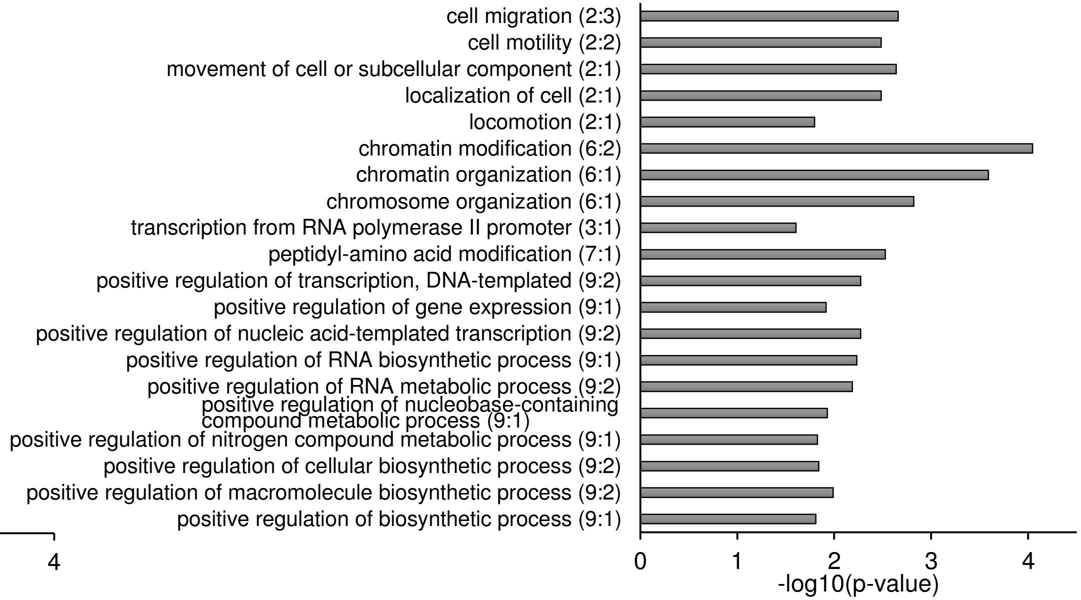

Figure S12

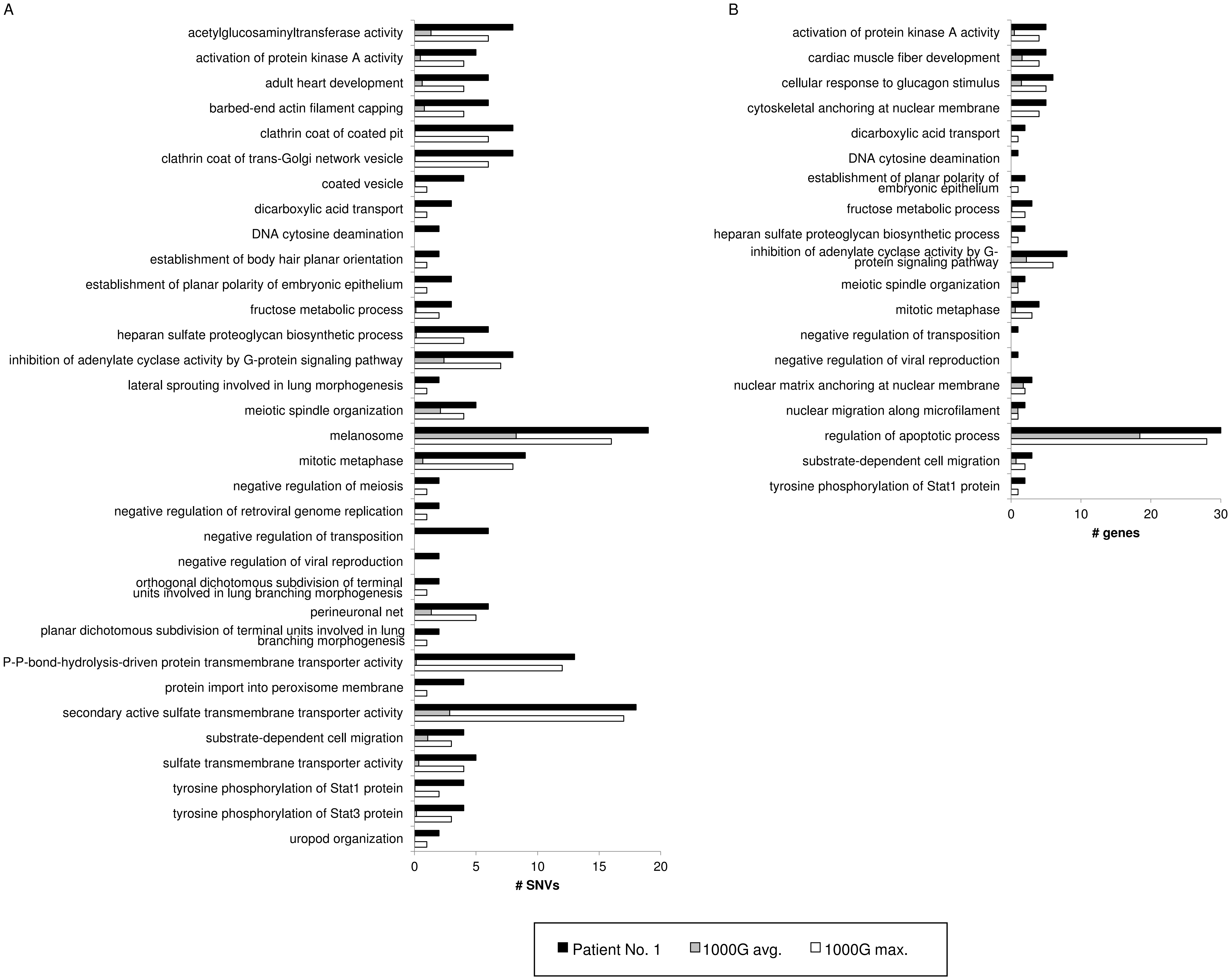
A
acetylglucosaminyltransferase activity
activation of protein kinase A activity
adult heart development
barbed-end actin filament capping
clathrin coat of coated pit
clathrin coat of trans-Golgi network vesicle
coated vesicle
dicarboxylic acid transport
DNA cytosine deamination
establishment of body hair planar orientation
establishment of planar polarity of embryonic epithelium
fructose metabolic process
heparan sulfate proteoglycan biosynthetic process
inhibition of adenylate cyclase activity by G-protein signaling pathway
lateral sprouting involved in lung morphogenesis
meiotic spindle organization
melanosome
mitotic metaphase
negative regulation of meiosis
negative regulation of retroviral genome replication
negative regulation of transposition
negative regulation of viral reproduction
orthogonal dichotomous subdivision of terminal units involved in lung branching morphogenesis
perineuronal net
planar dichotomous subdivision of terminal units involved in lung branching morphogenesis
P-P-bond-hydrolysis-driven protein transmembrane transporter activity
protein import into peroxisome membrane
secondary active sulfate transmembrane transporter activity
substrate-dependent cell migration
sulfate transmembrane transporter activity
tyrosine phosphorylation of Stat1 protein
tyrosine phosphorylation of Stat3 protein
uropod organization
0
5
10
15
20
SNVs
B
activation of protein kinase A activity
cardiac muscle fiber development
cellular response to glucagon stimulus
cytoskeletal anchoring at nuclear membrane
dicarboxylic acid transport
DNA cytosine deamination
establishment of planar polarity of embryonic epithelium
fructose metabolic process
heparan sulfate proteoglycan biosynthetic process
inhibition of adenylate cyclase activity by G-protein signaling pathway
meiotic spindle organization
mitotic metaphase
negative regulation of transposition
negative regulation of viral reproduction
nuclear matrix anchoring at nuclear membrane
nuclear migration along microfilament
regulation of apoptotic process
substrate-dependent cell migration
tyrosine phosphorylation of Stat1 protein
0
10
20
30
genes
Patient No. 1
1000G avg.
1000G max.

Figure S13

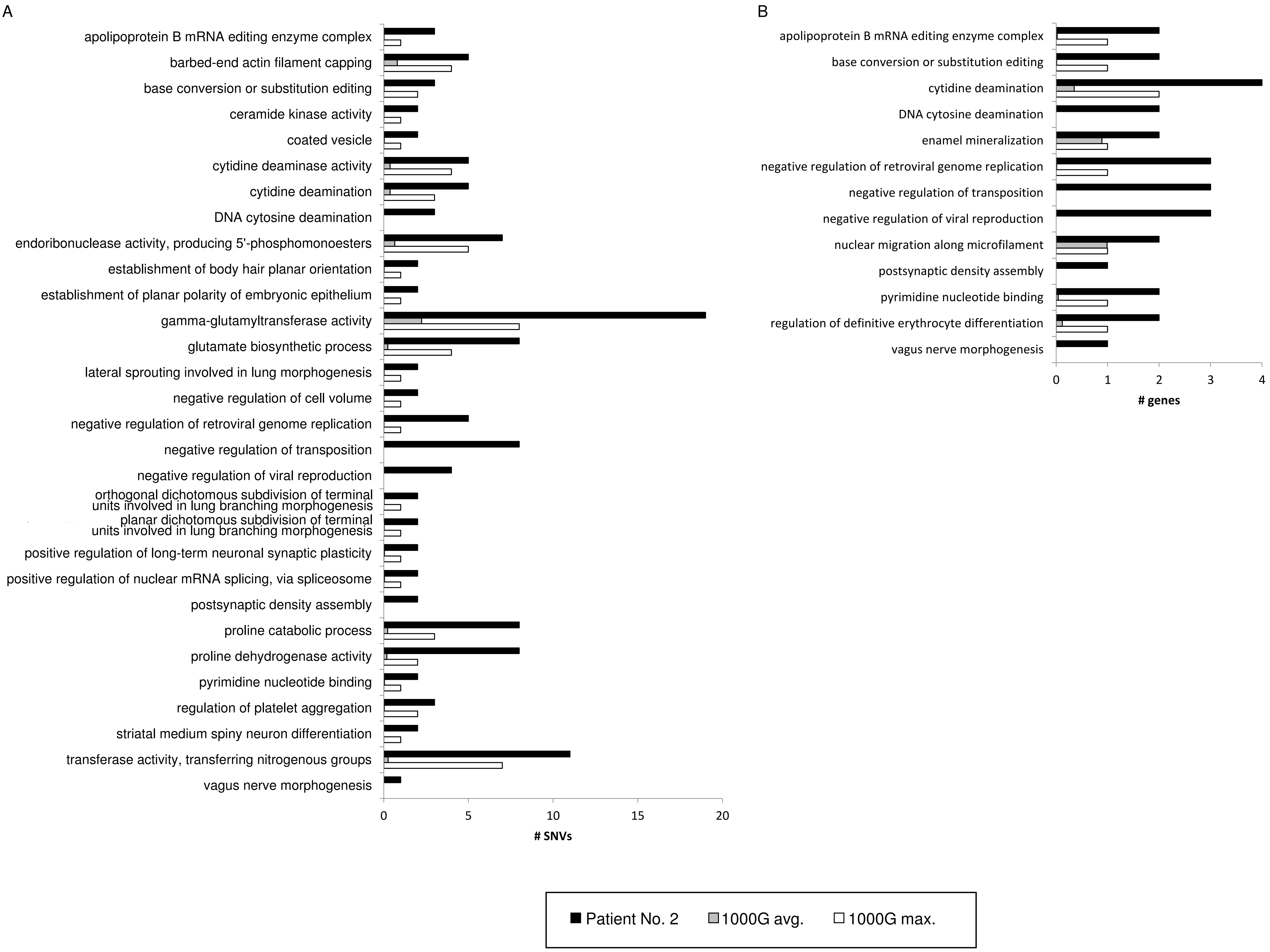
A
apolipoprotein B mRNA editing enzyme complex
barbed-end actin filament capping
base conversion or substitution editing
ceramide kinase activity
coated vesicle
cytidine deaminase activity
cytidine deamination
DNA cytosine deamination
endoribonuclease activity, producing 5'-phosphomonoesters
establishment of body hair planar orientation
establishment of planar polarity of embryonic epithelium
gamma-glutamyltransferase activity
glutamate biosynthetic process
lateral sprouting involved in lung morphogenesis
negative regulation of cell volume
negative regulation of retroviral genome replication
negative regulation of transposition
negative regulation of viral reproduction
orthogonal dichotomous subdivision of terminal units involved in lung branching morphogenesis
planar dichotomous subdivision of terminal units involved in lung branching morphogenesis
positive regulation of long-term neuronal synaptic plasticity
positive regulation of nuclear mRNA splicing, via spliceosome
postsynaptic density assembly
proline catabolic process
proline dehydrogenase activity
pyrimidine nucleotide binding
regulation of platelet aggregation
striatal medium spiny neuron differentiation
transferase activity, transferring nitrogenous groups
vagus nerve morphogenesis
0
5
10
15
20
SNVs
B
apolipoprotein B mRNA editing enzyme complex
base conversion or substitution editing
cytidine deamination
DNA cytosine deamination
enamel mineralization
negative regulation of retroviral genome replication
negative regulation of transposition
negative regulation of viral reproduction
nuclear migration along microfilament
postsynaptic density assembly
pyrimidine nucleotide binding
regulation of definitive erythrocyte differentiation
vagus nerve morphogenesis
0
1
2
3
4
genes
Patient No. 2
1000G avg.
1000G max.

Figure S14

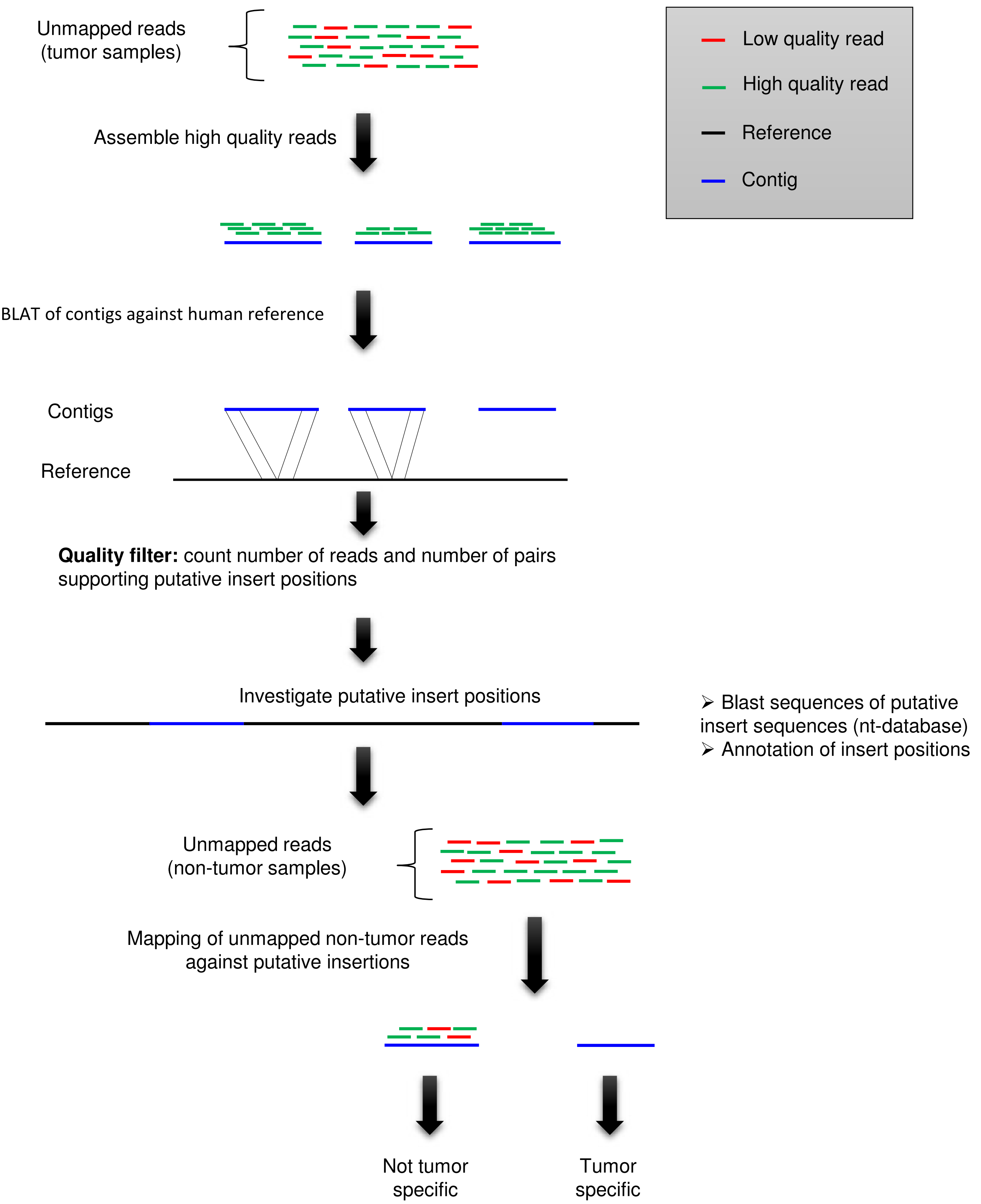
Unmapped reads
(tumor samples)
Low quality read
High quality read
Reference
Contig
Assemble high quality reads
BLAT of contigs against human reference
Contigs
Reference
Quality filter: count number of reads and number of pairs supporting putative insert positions
Investigate putative insert positions
➢ Blast sequences of putative insert sequences (nt-database)
➢ Annotation of insert positions
Unmapped reads
(non-tumor samples)
Mapping of unmapped non-tumor reads against putative insertions
Not tumor specific
Tumor specific

Figure S15

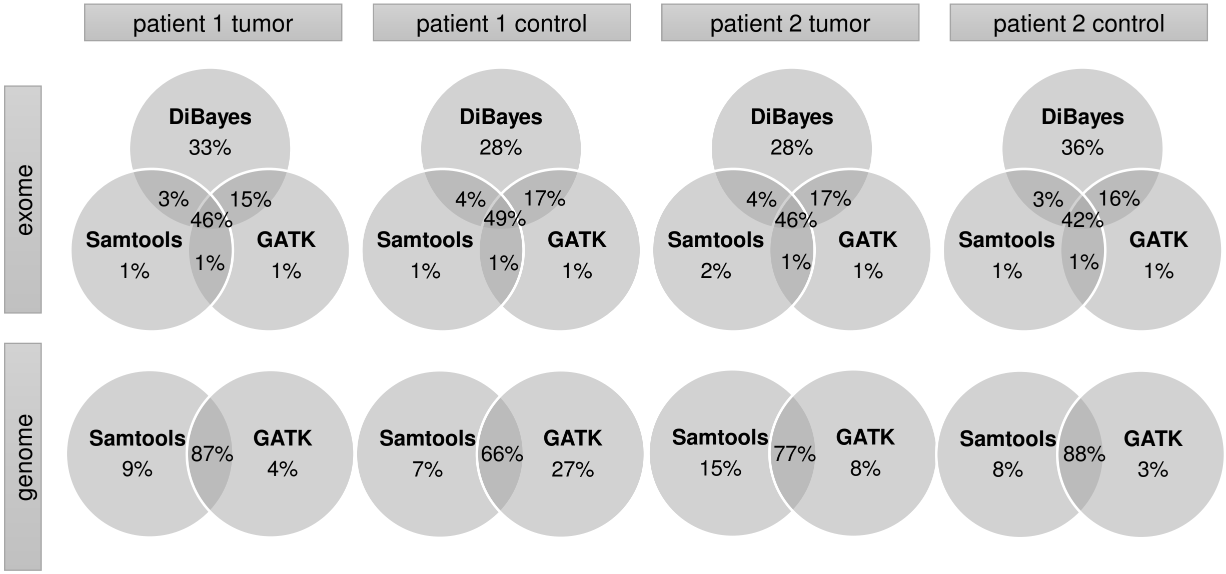

patient 1 tumor
patient 1 control
patient 2 tumor
patient 2 control
exome
genome
DiBayes
33%
3%
15%
46%
Samtools
1%
1%
GATK
1%
DiBayes
28%
4%
17%
49%
Samtools
1%
1%
GATK
1%
DiBayes
28%
4%
17%
46%
Samtools
2%
1%
GATK
1%
DiBayes
36%
3%
16%
42%
Samtools
1%
1%
GATK
1%
Samtools
9%
87%
GATK
4%
Samtools
7%
66%
GATK
27%
Samtools
15%
77%
GATK
8%
Samtools
8%
88%
GATK
3%

Figure S16

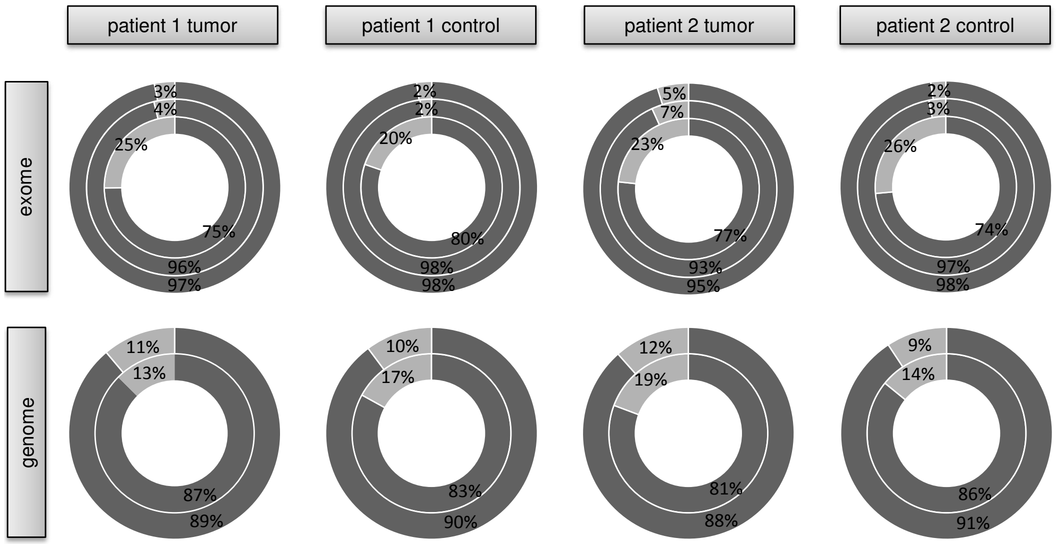

patient 1 tumor
patient 1 control
patient 2 tumor
patient 2 control
exome
genome
3%
4%
25%
75%
96%
97%
2%
2%
20%
80%
98%
98%
5%
7%
23%
77%
93%
95%
2%
3%
26%
74%
97%
98%
11%
13%
87%
89%
10%
17%
83%
90%
12%
19%
81%
88%
9%
14%
86%
91%

Figure S17

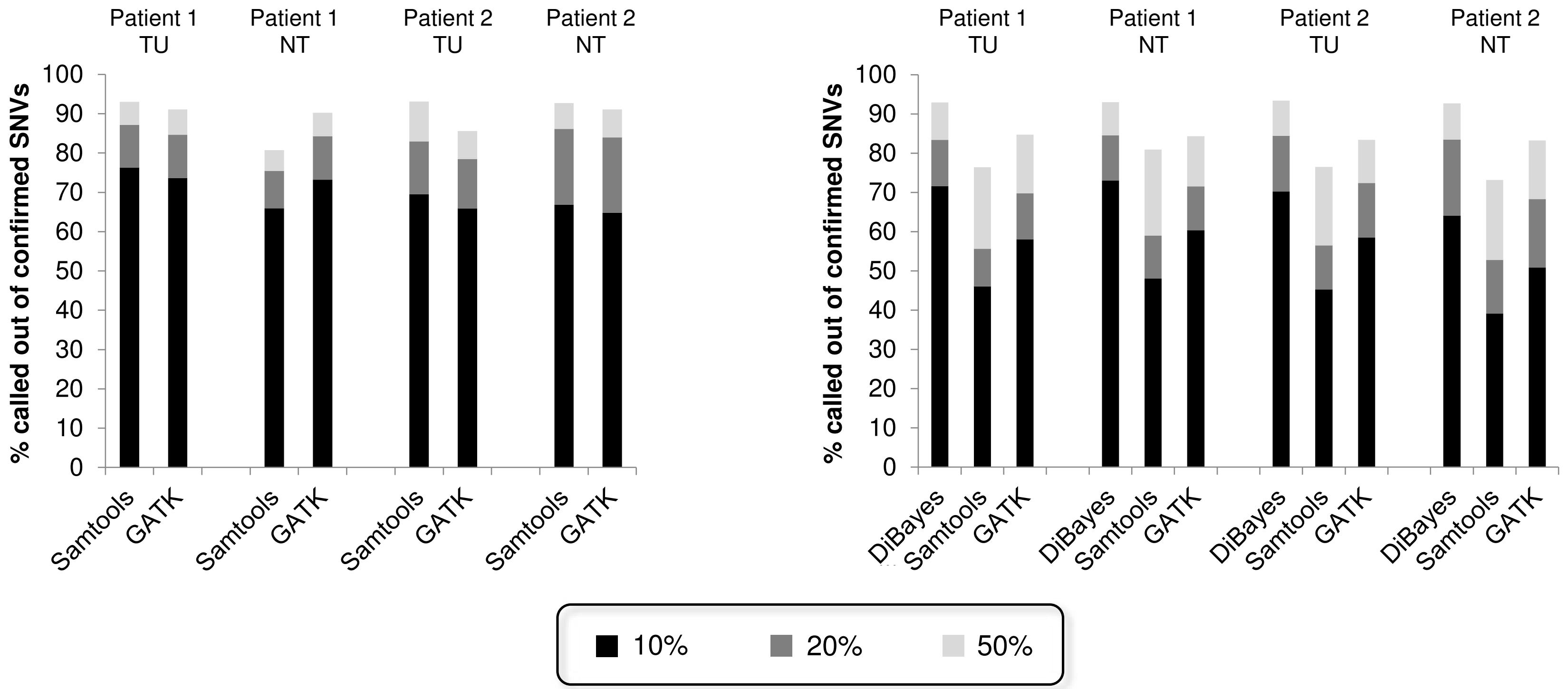

Number of confirmed SNVs (10%, 20%, 50%)

	Patient 1 TU	Patient 1 NT	Patient 2 TU	Patient 2 NT
**WGS -Samtools**	67426 / 63229 / 30967	65751 / 62894 / 29815	67969 / 64310 / 34095	78203 / 74402 / 34643
**WGS – GATK**	65010 / 61420 / 30324	72990 / 70246 / 33336	64420 / 60855 / 31352	75691 / 72530 / 34048
**WES – DiBayes**	63249 / 60504 / 30944	72779 / 70446 / 34944	68645 / 65459 / 34216	74895 / 72090 / 34653
**WES – Samtools**	40667 / 40363 / 25447	47894 / 49151 / 36259	44308 / 43826 / 28385	45759 / 45572 / 27353
**WES - GATK**	51276 / 50577 / 28209	60156 / 59624 / 31131	57220 / 56097 / 31468	59430 / 58950 / 31120

Figure S18

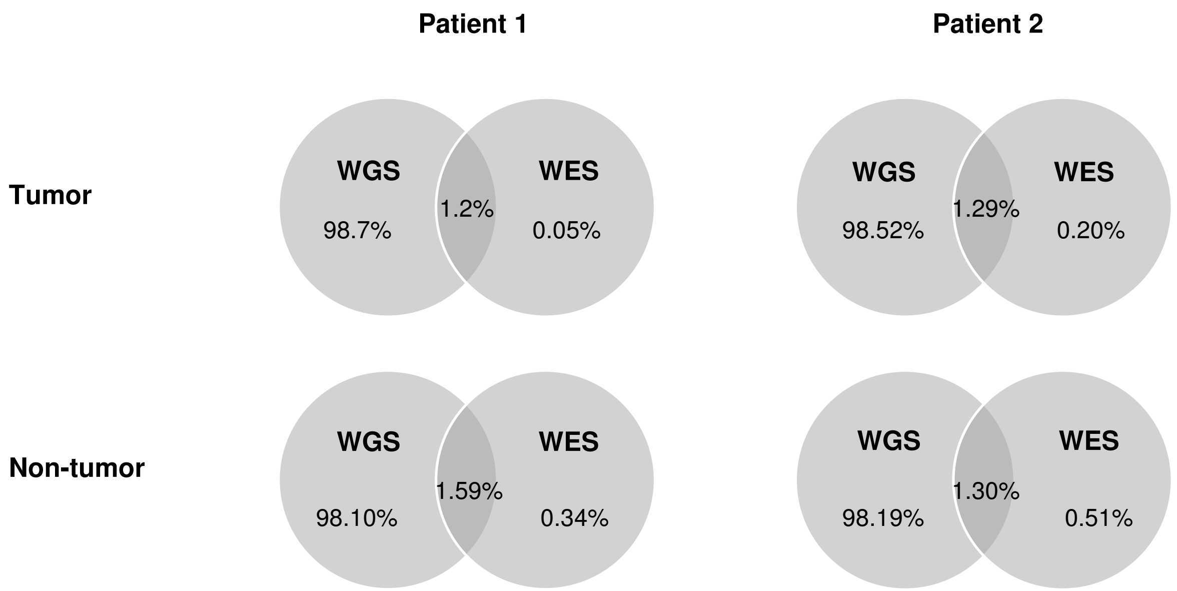

Patient 1
Patient 2
Tumor
WGS
WES
98.7%
1.2%
0.05%
WGS
WES
98.52%
1.29%
0.20%
Non-tumor
WGS
WES
98.10%
1.59%
0.34%
WGS
WES
98.19%
1.30%
0.51%

Figure S19

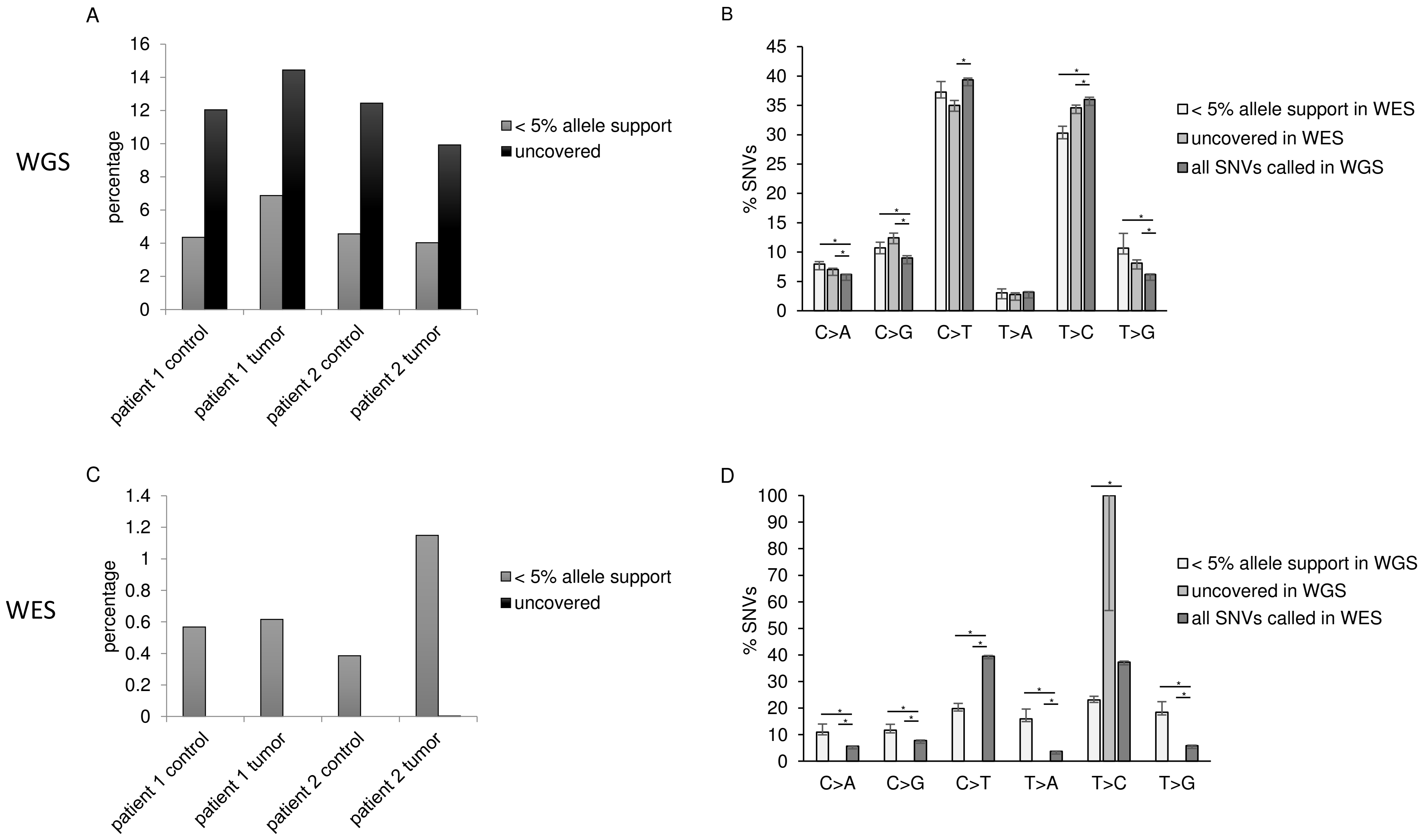

A
B
C
D
WGS
WES
percentage
% SNVs
< 5% allele support
uncovered
< 5% allele support in WES
uncovered in WES
all SNVs called in WGS
< 5% allele support in WGS
uncovered in WGS
all SNVs called in WES
patient 1 control
patient 1 tumor
patient 2 control
patient 2 tumor
C>A
C>G
C>T
T>A
T>C
T>G

Figure S20

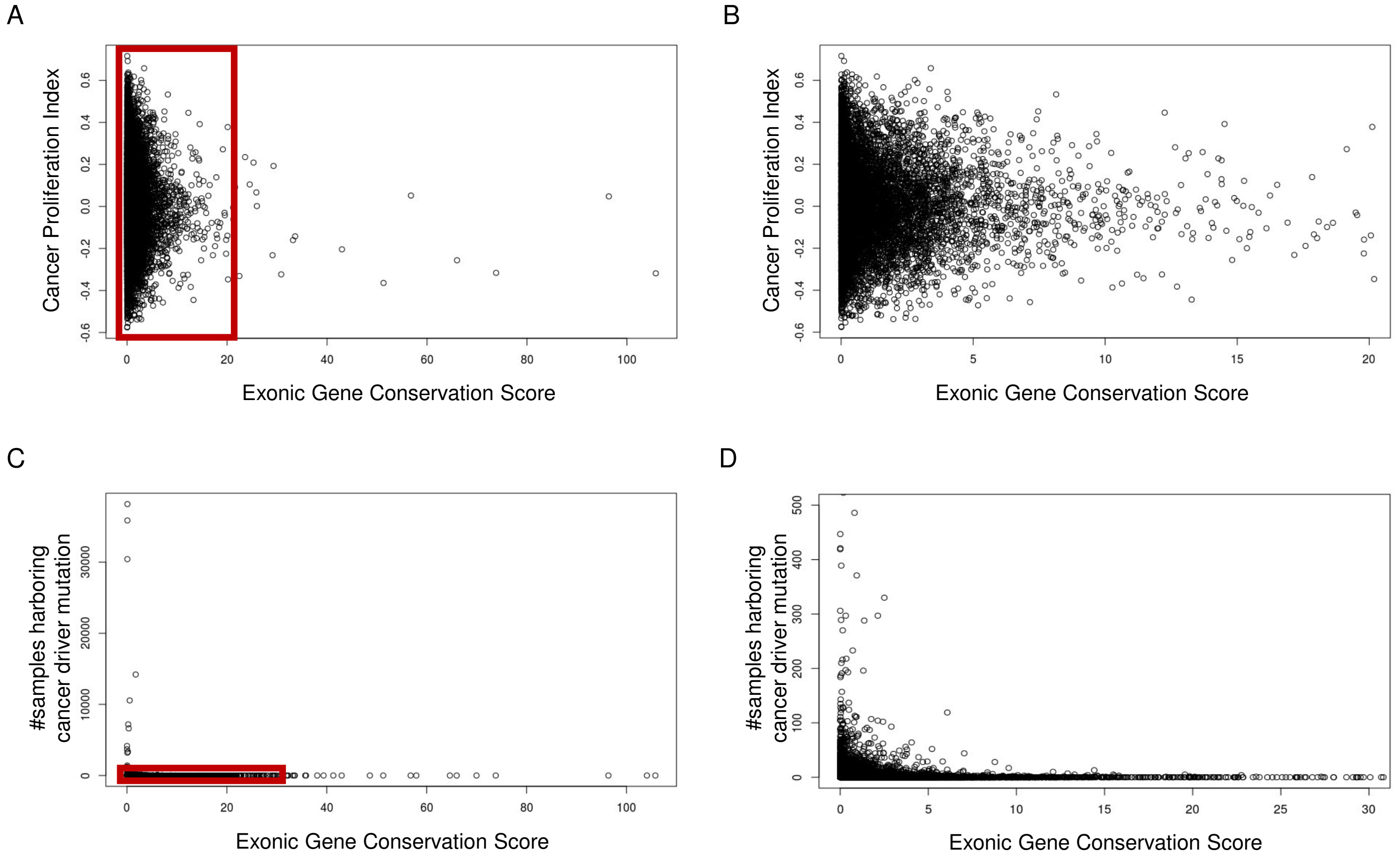
A
B
C
D
Cancer Proliferation Index
Exonic Gene Conservation Score
#samples harboring cancer driver mutation
