# Supplementary material for: Interpreting whole genome and exome sequencing data of individual gastric cancer samples
Source: BMC Genomics. 2017 Jul 6;18:517. doi: 10.1186/s12864-017-3895-z (PMC5501078; doi:10.1186/s12864-017-3895-z)
Supplement: Supplementary file 2 — Table S1- Table S9. (PDF 364 kb) [file 12864_2017_3895_MOESM2_ESM.pdf]

Additional File 2

Interpreting whole genome and exome sequencing data of individual gastric cancer samples

Daniela Esser[1,4], Niklas Holze[2], Jochen Haag[2], Stefan Schreiber[1,3], Sandra Krüger[2], Viktoria Warneke[2], Philip Rosenstiel[1†] and Christoph Röcken[2*†]

[1] Institute for Clinical Molecular Biology, Christian-Albrechts-University, 24105 Kiel, Germany
[2] Institute for Pathology, Christian-Albrechts-University, 24105 Kiel, Germany
[3] Department of General Internal Medicine, University Hospital Schleswig-Holstein, 24105 Kiel, Germany
[4] Institute for Experimental Medicine, Christian-Albrechts-University, 24105 Kiel, Germany

Table S1

Polymerase chain reaction and sequencing primers used for validation of single nucleotide variations.

Table S2

Somatic single nucleotide variations (SNVs). The following abbreviations were used: chr = chromosome, pos = chromosomal position, gene = affected gene, effect = effect of SNV on amino acid, ref nt = annotated nucleotide in reference sequence, observ. nt = called allele, ref aa = amino acid in reference, observ. aa = amino acid based on sequence with SNV, pos aa = position of amino acid within the gene, Sift = Sift damaging prediction score, PolyPhen = PolyPhen2 damaging prediction score, PhyloP = PhyloP conservation score, ECS = conservation score based on exonic regions of the gene, 1000G freq = frequency of SNV within the 1000 Genomes project, g-st = information, if SNV was called with Samtools in the genome sequencing data, e-st = information, if SNV was called with Samtools in the exome

sequencing data, e-bs = information, if SNV was called with DiBayes / Bioscope in the exome sequencing data, cosmic=information whether the gene is listed in the cancer gene consensus list of the COSMIC database.(http://cancer.sanger.ac.uk/census) (A-B) Novel SNVs filtered by quality: All SNVs were called in the exome with Samtools and DiBayes / Bioscope and in the genome data with Samtools. Furthermore, all SNVs were supported by more than 20% of the high quality bases and were predicted as damaging by either PolyPhen2 or Sift. (C-D) Novel SNVs, which were called with at least one caller, supported by more than 5% of the reads in the genome as well as in the exome tumor data and existing in maximum 5% of the reads in the genome as well as in the exome data of the matching control samples. Furthermore, all SNVs result (i) in a nonsense amino acid change or (ii) were predicted as damaging by Sift or PolyPhen2 and were either at a conserved position (PhyloP) or in a conserved gene (ECS). (E-F) Novel SNVs, which were called with at least one caller and resulting either in a nonsense amino acid change or were predicted as damaging by Sift or PolyPhen2 or were at a conserved position or in a conserved gene.

Table S3

Filtered somatic small indels. The following abbreviations were used: chr = chromosome, start = start position, end = end position, gene = affected gene, type = type of indel: insertion/deletion and effect on reading frame, ref nt = nucleotides in reference sequence, observed nt = called bases, ref aa = amino acid based on reference sequence, aa pos = position of amino acid within the gene, called in exome? = information whether indel was called in the exome data, called in the genome? = information whether indel was called in the genome data, cosmic =

information whether the gene is listed in the cancer gene consensus list of the COSMIC database.

Table S4

Somatic intragenic large structural variants. (A-B) Inversions. (C-D) Interchromosomal translocations: Intersecting translocations having breakpoints closer than 25,000 bp to each other were merged to one entry. (E-F) Large deletions: All genes having deletions (>5bp) with a total length larger 40bp.

Table S5

Clinico-pathological characteristics of patients with a *GNAS*-mutation.

Table S6

SNVs with failed validations using Pyromark sequencing. All variants were called in the MSI WES data, but not in the WGS data. The table shows the mutant allele fractions based on all sequencing data sets.

Table S7

SNVs with failed validations using Pyromark sequencing. All variants were called in the MSS WES data, but not in the WGS data. The table shows the mutant allele fractions based on all sequencing data sets.

Table S8

Putative positions for large insertions. Exclusively positions with adjacent gene closer than 100kb are reported

Table S9

Number of called raw somatic SNVs (not quality filtered, inter- and intragenic, synonymous + non-synonymous, etc.). In this case, somatic means that the SNV was not detected in the corresponding non-tumor samples sequenced with the same technology.

Table S1 PCR and sequencing primers used for validation of SNVs

Gene	Mutation	Primer	Sequence
DROSHA	p.Q1089X	DROSHA-PCR-for	5´-GGGAGAAGGAATTTTACAAAACAC-3´
		DROSHA-PCR-rev	5´-TCCAATTGCTTCTTCAAACTCA-3´
		DROSHA-Sequencing	5´-TATTTCTATTTTCCTGTAGC-3´
MSH4	p.A174T	MSH4-PCR-for	5´-TTGTAGAAGGGAGAGGACTTGC-3´
		MSH4-PCR-rev	5´-CCTTTGCATATGTTGTGTTGTCTG-3´
		MSH4-Sequencing	5´-GTTTTTTAAATCAATACTTG-3´
RERE	p.Q500X	RERE-PCR-for	5´-CCCACCCCCACTATGTGC-3´
		RERE-PCR-rev	5´-GGCAGGCGTACCCCTTCA-3´
		RERE-Sequencing	5´-TACCCCTTCAGCTCC-3´
ROS1	p.Q925R	ROS1-PCR-for	5´-CTCTGTTTTGGAACCAGCCAGATT-3´
		ROS1-PCR-rev	5´-AGGGGCTTAAGGGATGTCTGAATA-3´
		ROS1-Sequencing	5´-GAACCAGCCAGATTTAAT-3´
TACC2	p.T502M	TACC2-PCR-for	5´-TTGAGATCCCAGCCAGTGCTAT-3´
		TACC2-PCR-rev	5´-CTTACCACCTCCACCCCTGAA-3´
		TACC2-Sequencing	5´-TGAACTTACGTCTTTAGGG-3´
TYRO3	p.R333H	TYRO-PCR-for	5´-AGGGCAGGGGTCTTAGCAATCT-3´
		TYRO-PCR-rev	5´-ACTTCTTCCCACTCCAAGATGAGG-3´
		TYRO-Sequencing	5´-TGAGGCCTGAATCTGT-3´

Table S2 A: High quality SNVs patient 1

chr	pos	gene	effect	ref nt	observed nt	ref aa	observed aa	pos aa	Sift	PolyPhen	PhyloP	ECS	1000G freq	g-st	e-st	e-bs
1	8424848	*RERE*	nonsense	G	A	Q	X	500	0.17	0.74	0.9996	0.1114	0.1114	yes	yes	yes
5	31423048	*DROSHA*	nonsense	G	A	Q	X	1052	0.01			0.6416	0.6416	yes	yes	yes
7	150647363	*KCNH2*	nonsynony	G	A	P	L	424	0.00	0.95	0.9987	0.3248	0.3248	yes	yes	yes
19	49713590	*TRPM4*	nonsyn	C	T	R	C	941	0.02	1.00	0.9989	0.1508	0.1508	yes	yes	yes
21	43708011	*ABCG1*	nonsyn	C	T	A	V	329	0.03	0.14	0.9968	0.0142	0.0142	yes	yes	yes

Table S2 B: High quality SNVs patient 2

chr	pos	gene	effect	ref nt	observed nt	ref aa	observed aa	pos aa	Sift	PolyPhen	PhyloP	ECS	1000G freq	g-st	e-st	e-bs
14	100363521	*EML1*	nonsense	C	G	Y	X	239	0.00	0.74	0.9984	1.7618		yes	yes	yes

Table S2 C SNV table patient 1 (damaging & conserved)

chr	pos	gene	effect	ref nt	observ. nt	ref aa	observ. aa	pos aa	Sift	PolyPhen	PhyloP	ECS	g-st	e-st	e-bs	cosmic
1	8424848	RERE	nonsense	G	A	Q	X	500	0.17	0.74	0.9996	0.1114	yes	yes	yes	no
1	14108317	PRDM2	nonsyn	G	A	D	N	1142	0	0.7	0.9998	2.0446	no	no	yes	no
1	19449524	UBR4	nonsyn	G	A	R	C	3207	0	1	0.9996	0.4922	yes	no	no	no
1	26448895	PDIK1L	nonsyn	G	A	E	K	285	0.01	1	0.9997	0.0012	no	no	yes	no
1	76272758	MSH4	nonsyn	G	A	A	T	174	0	1	0.9997	1.0239	no	no	yes	no
1	149859238	HIST2H2AB	nonsyn	T	C	T	A	77		0.57	0.9976	0	yes	no	no	no
1	156268899	VHLL	nonsense	C	A	E	X	28		0.45	0.2208	0.0443	no	no	yes	no
1	161011635	USF1	nonsyn	G	A	A	V	93	0.01	0.06	0.9996	0.0094	no	yes	yes	no
1	183095328	LAMC1	nonsyn	G	A	G	S	959	0	0.99	0.9997	1.3632	no	no	yes	no

2	100623269	AFF3	nonsyn	G	A	A	V	258	0	0.96	0.9996	1.0244	no	no	yes	yes
3	33427020	FBXL2	nonsyn	G	A	A	T	330	0	0.93	0.9998	0.0168	yes	no	no	no
3	52548457	STAB1	nonsyn	G	A	R	H	1208	0.01	0.99	0.9996	2.9546	yes	no	no	no
3	107517481	BBX	nonsyn	G	T	S	I	792	0	1	0.9998	0.048	yes	no	no	no
3	122003133	CASR	nonsyn	G	A	G	S	778	0.04	0.98	0.9997	1.8264	no	no	yes	no
3	178936082	PIK3CA	nonsyn	G	A	E	K	542	0.04	0.89	0.9997	0.2407	no	no	yes	yes
4	111397920	ENPEP	nonsyn	G	A	G	D	117	0	1	0.9997	2.3104	no	no	yes	no
5	31423048	DROSHA	nonsense	G	A	Q	X	1052	0.01			0.6416	yes	yes	yes	yes
5	177642326	AGXT2L2	nonsense	G	A	Q	X	345	0.35	0.69	0.9767	0.3738	yes	no	no	no
7	99797878	STAG3	nonsyn	G	T	R	L	566	0	0.18	0.9997	0.6811	yes	no	no	no
9	5126730	JAK2	nonsyn	G	A	R	H	1113	0	1	0.9995	0.0818	yes	no	no	yes
9	104356830	PPP3R2	nonsyn	G	A	T	M	128	0.01	0	0.1469	0.0099	yes	no	no	no
9	115805897	ZFP37	nonsense	G	T	S	X	334	0.04	0.74	0.9994	1.8508	no	no	yes	no
9	127298179	NR6A1	nonsyn	G	A	P	S	348	0.02	1	0.9997	0.0768	yes	no	no	no
9	129594868	ZBTB43	nonsyn	G	A	R	H	27	0	0.98	0.9998	0.0047	yes	no	no	no
10	12191899	SEC61A2	nonsyn	C	T	T	M	112	0.04	0.25	0.998	0.0064	yes	no	no	no
11	12183953	MICAL2	nonsyn	G	A	C	Y	84	0	1	0.9998	0.3538	yes	no	no	no
11	73022234	ARHGEF17	nonsense	C	T	R	X	851	1	0.72	0.8847	0.6167	no	no	yes	no
12	56755353	APOF	nonsense	G	A	R	X	213	1			1.0174	yes	no	no	no
12	69981309	CCT2	nonsense	C	T	R	X	10	1	0.73	0.9863	0.1673	yes	no	no	no
12	109278801	DAO	nonsyn	G	A	G	R	7	0	1	0.9995	0.0704	yes	no	no	no
14	24839069	NFATC4	nonsense	C	A	Y	X	155	0	0.6	0.177	1.3149	no	no	yes	no
15	64506145	CSNK1G1	nonsyn	T	G	H	P	208	0	1	0.9985	0.0076	no	no	yes	no
18	18619508	ROCK1	nonsense	G	A	R	X	326		0.73	0.99	0.136	yes	no	no	no
18	31432849	NOL4	nonsyn	C	T	R	Q	340	0	0.79	0.9991	0.0073	no	no	yes	no
19	9271885	ZNF317	nonsense	C	T	R	X	490	1	0.7	0.7709	0.4575	yes	no	no	no
20	16486763	KIF16B	nonsyn	G	A	R	C	258	0	1	0.9996	1.5824	yes	no	no	no
20	31671526	BPIFB4	nonsense	G	T	G	X	175	0.03	0.73	0.9977	11.229	no	no	yes	no
20	43243244	PKIG	nonsyn	G	A	R	Q	16	0.04	1	0.9996	0.0157	yes	no	no	no

20	62560874	DNAJC5	nonsyn	C	T	A	V	106	0.15	0.9	0.9989	0	no	no	yes	no
X	24552101	PDK3	nonsyn	G	A	R	H	378	0	0.98	0.9997	0.0114	no	yes	yes	no
X	106459930	CXorf41	nonsyn	G	T	M	I	61	0.34	1	0.9692	0.0029	no	no	yes	no

Table S2 D: SNV table patient 2 (damaging & conserved)

chr	pos	gene	effect	ref nt	observ. nt	ref aa	observ. aa	pos aa	Sift	PolyPhen	PhyloP	ECS	g-st	e-st	e-bs	cosmic
10	8106046	*GATA3*	nonsyn	T	G	L	R	290	0	0.98	0.9979	0.0018	no	no	yes	yes
14	100363521	*EML1*	nonsense	C	G	Y	X	239	0	0.74	0.9984	1.7618	yes	yes	yes	no
16	81398615	*GAN*	nonsyn	G	A	A	T	425	0.01	0.74	0.9996	0.0272	yes	yes	no	no
17	74276151	*QRICH2*	nonsyn	G	A	P	S	1405	0.01	1	0.9996	5.6252	no	no	yes	no
19	46094563	*GPR4*	nonsyn	C	T	V	M	188	0.02	0.01	0.9979	0.0055	yes	no	no	no
20	377023	*TRIB3*	nonsyn	G	C	A	P	256	0.01	0.84	0.9997	0.8909	no	no	yes	no

Table S2 E: SNV table patient 1 (damaging or conserved, additional SNVs to Suppl. Table 2C)

chr	pos	gene	effect	ref nt	observ. nt	ref aa	observ. aa	pos aa	Sift	PolyPhen	PhyloP	ECS	g-st	e-st	e-bs	cosmic
1	42047914	*HIVEP3*	nonsyn	T	G	N	T	852	0	0.99	0.9976	3.1492	no	no	yes	no
1	75805297	*SLC44A5*	nonsyn	T	C	D	G	24	0	1	0.9983	0.0532	no	no	yes	no
1	113246418	*RHOC*	nonsyn	C	T	A	T	2	0.03	0	0.9984	0.0243	no	no	yes	no
1	153615804	*CHTOP*	nonsyn	C	T	R	C	170	0	0.85	0.9993	0.07	yes	no	no	no
1	156146493	*SEMA4A*	nonsyn	C	A	P	Q	532	0.04	0.64	0.9979	0.2635	no	no	yes	no
1	183087260	*LAMC1*	nonsyn	C	T	R	C	657	0	1	0.9979	1.3632	yes	yes	yes	no
1	222832120	*MIA3*	nonsyn	C	T	A	V	1555	0.13	0.98	0.9991	1.5735	no	yes	yes	no
2	21233882	*APOB*	nonsyn	T	C	H	R	1953	0.88	0.82	0.2371	2.6255	yes	no	yes	no
2	71654216	*ZNF638*	nonsyn	A	G	I	M	1739	0	0.82	0.9145	1.2099	yes	no	no	no
2	103068269	*IL18RAP*	nonsyn	A	C	R	S	476	0	1	0.9392	0.0611	yes	yes	no	no

2	170088278	*LRP2*	nonsyn	A	G	C	R	1725	0	0.88	0.9986	1.3948	no	no	yes	no
2	208994242	*CRYGC*	nonsyn	G	A	R	W	59	0.1	1	0.9392	0.2053	yes	yes	yes	no
2	238275374	*COL6A3*	nonsyn	G	A	A	V	1212		0.57	0.9994	2.0665	yes	no	no	no
3	9780797	*BRPF1*	nonsyn	T	G	S	R	238	0.02	0.02	0.792	0.0143	yes	no	no	no
3	47098793	*SETD2*	nonsyn	G	A	H	Y	2161	0	1	0.9957	0.871	no	yes	yes	yes
3	111697936	*ABHD10*	nonsyn	G	A	A	T	10	0.06	0.46	0.9877	0.3632	yes	no	yes	no
3	119451232	*C3orf15*	nonsyn	G	T	Q	H	370	0	0.99	0.9684	1.6671	no	no	yes	no
3	127335771	*MCM2*	nonsyn	C	T	A	V	528	0	1	0.9989	0.2061	yes	no	no	no
3	183210369	*KLHL6*	nonsyn	T	C	T	A	493	0.04	0.02	0.8691	0.0498	yes	no	yes	no
4	81124574	*PRDM8*	nonsyn	C	T	A	V	653	0.02	0.13	0.9983	0.9291	yes	no	no	no
4	129809878	*SCLT1*	nonsyn	G	A	R	C	654	0	1	0.9995	2.3453	yes	no	no	no
5	1798796	*MRPL36*	nonsyn	C	T	R	Q	85	0.17	0.95	0.9467	0.2744	no	no	yes	no
5	71739960	*ZNF366*	nonsyn	C	T	E	K	620	0.06	0.78	0.9994	0.6001	no	no	yes	no
5	140166393	*PCDHA1*	nonsyn	G	T	S	I	173	0.01	0.55	0.8597	2.0306	no	yes	yes	no
5	140167635	*PCDHA1*	nonsyn	C	T	A	V	587	0.12	0.5	0.882	2.0306	yes	no	no	no
6	26091725	*HFE*	nonsyn	G	A	R	Q	87	0.08	1	0.7991	0.604	no	no	yes	no
6	109774937	*MICAL1*	nonsyn	C	T	V	M	124	0	0.25	0.999	2.9512	yes	no	no	no
7	5545126	*FBXL18*	nonsyn	C	T	V	I	52	0	0.73	0.9975	0.0464	yes	no	no	no
7	103029485	*SLC26A5*	nonsyn	G	T	A	D	463	0	0.99	0.9995	0.0526	yes	no	no	no
7	148288147	*C7orf33*	nonsyn	G	A	A	T	44	0	0.01	0.9597	0.6387	no	no	yes	no
7	150647363	*KCNH2*	nonsyn	G	A	P	L	424	0	0.95	0.9987	0.3248	yes	yes	yes	no
7	151962282	*MLL3*	nonsyn	G	A	A	V	342	0.29	0.96	0.9989	0.2818	no	no	yes	no
8	10583282	*SOX7*	nonsyn	G	A	T	M	378	0	1	0.9994	0.1973	yes	no	no	no
8	68044263	*CSPP1*	nonsyn	C	T	P	L	587	0.02	0.42	0.8906	0.4499	yes	no	no	no
9	32635480	*TAF1L*	nonsyn	C	T	G	D	33		0.84	0.9092	0.2873	yes	no	no	no
9	96846997	*PTPDC1*	nonsyn	C	T	A	V	62	0	0.89	0.9992	0.1736	no	no	yes	no
10	120810812	*EIF3A*	nonsyn	G	A	R	C	740	0	0.73	0.9994	0.2848	yes	no	no	no
10	123971211	*TACC2*	nonsyn	C	T	T	M	502	0	1	0.998	2.9859	yes	yes	yes	no
10	128192969	*C10orf90*	nonsyn	A	G	V	A	267	0.01	0.02	0.9365	2.6803	yes	no	no	no

11	8122493	*TUB*	nonsyn	C	T	R	C	446	0	1	0.999	0.0723	no	no	yes	no
11	9530327	*ZNF143*	nonsyn	C	T	R	W	437	0	1	0.922	2.14	yes	no	yes	no
12	430249	*KDM5A*	nonsyn	C	T	R	Q	818	0.78	0.77	0.9992	0.5499	yes	no	no	yes
12	10783802	*STYK1*	nonsyn	G	T	A	D	98	0.02	0	0.9047	1.7457	yes	no	no	no
12	56221845	*DNAJC14*	nonsyn	G	A	R	C	200	0	1	0.9877	0.0371	no	yes	yes	no
12	91371921	*EPYC*	nonsyn	C	T	G	D	95	0.12	0.84	0.9991	0.478	yes	no	yes	no
13	24864923	*SPATA13*	nonsyn	A	G	D	G	369	0	0.78	0.9983	0.3041	yes	no	no	no
13	52667265	*NEK5*	nonsyn	T	C	H	R	378	0.22	0.98	0.7847	0.6349	yes	no	no	no
13	98668017	*IPO5*	nonsyn	A	C	E	D	843	0.02	0.99	0.9857	0.0206	yes	no	no	no
13	101881755	*NALCN*	nonsyn	T	C	T	A	539	0.01	0.97	0.9975	0.0414	yes	yes	yes	no
13	102568880	*FGF14*	nonsyn	A	C	L	R	39	0.01	0.56	0.9983	0.0501	no	no	yes	no
15	25584307	*UBE3A*	nonsyn	C	T	A	T	846	0.01	0.62	0.9982	0.0128	yes	yes	no	no
15	28391389	*HERC2*	nonsyn	G	A	R	W	3668	0	1	0.9995	0.1469	yes	no	no	no
15	41860451	*TYRO3*	nonsyn	G	A	R	H	333	0.1	0.88	0.0693	0.7283	yes	yes	yes	no
15	42002919	*MGA*	nonsyn	G	A	R	H	819	0			0.873	yes	no	no	no
15	62273608	*VPS13C*	nonsyn	A	G	V	A	657	0	0.02	0.9991	1.101	yes	no	no	no
15	80743328	*ARNT2*	nonsyn	C	T	R	C	47	0	0.99	0.9976	0.2047	yes	yes	yes	no
16	15811127	*MYH11*	nonsyn	G	A	R	W	1792	0	1	0.9956	0.5946	yes	no	no	yes
16	27268842	*NSMCE1*	nonsyn	C	T	R	H	17	0.05	0.72	0.9984	3.5112	no	yes	yes	no
16	50744711	*NOD2*	nonsyn	G	C	V	L	297	0.14	0.17	0.9996	0.75	no	no	yes	no
16	67264651	*FHOD1*	nonsyn	C	T	R	H	904	0.02	0.75	0.8367	0.2477	yes	no	no	no
16	80667028	*CDYL2*	nonsyn	C	T	R	H	241	0	1	0.9983	0.0578	no	no	yes	no
17	47656505	*NXPH3*	nonsyn	G	A	R	Q	201	1	0.02	0.9992	0.004	no	no	yes	no
17	48601125	*MYCBPAP*	nonsyn	G	A	D	N	582	0.06	0.49	0.9929	6.6175	no	no	yes	no
17	61882450	*DDX42*	nonsyn	A	G	E	G	214	0.02	0.83	0.9993	0.1547	no	no	yes	no
18	48452143	*ME2*	nonsyn	C	T	R	C	397	0	0.95	0.9381	0.4046	no	no	yes	no
19	15164741	*CASP14*	nonsyn	G	T	K	N	125	0	1	0.1449	0.0447	no	no	yes	no
19	18375992	*KIAA1683*	nonsyn	C	A	Q	H	786	0	1	0.0734	9.8172	yes	no	no	no
19	40362730	*FCGBP*	nonsyn	C	A	D	Y	5114	0.01	0.97	0.9985	2.9936	yes	no	no	no

19	40408082	*FCGBP*	nonsyn	G	T	L	M	1547		0.91	0.8254	2.9936	no	no	yes	no
19	47207446	*PRKD2*	nonsyn	C	T	R	Q	133	0	1	0.9987	1.8043	yes	no	no	no
19	49713590	*TRPM4*	nonsyn	C	T	R	C	941	0.02	1	0.9989	0.1508	yes	yes	yes	no
19	57176223	*ZNF835*	nonsyn	C	T	C	Y	115	0			3.6035	yes	no	no	no
20	76969	*DEFB125*	nonsyn	A	G	T	A	128	0.04	0.14	0.0011	0.1743	yes	no	no	no
20	33575653	*MYH7B*	nonsyn	A	T	D	V	493	0	0.72	0.9967	1.7309	no	yes	yes	no
20	42159033	*L3MBTL1*	nonsyn	C	T	T	M	299	0	1	0.999	0.1449	yes	no	no	no
21	15746149	*HSPA13*	nonsyn	A	T	V	E	402	0	0.93	0.9989	0.0183	no	no	yes	no
21	35468198	*SLC5A3*	nonsyn	C	A	S	Y	234	0.01	0	0.999	1.0678	no	no	yes	no
21	43708011	*ABCG1*	nonsyn	C	T	A	V	329	0.03	0.14	0.9968	0.0142	yes	yes	yes	no
22	32853263	*BPIFC*	nonsyn	C	A	R	S	37	0.01	0.98	0.2285	4.5579	no	no	yes	no
22	35943079	*RASD2*	nonsyn	T	G	S	A	75	0	1	0.9972	0.017	yes	no	no	no
22	50987896	*KLHDC7B*	nonsyn	T	G	L	R	434	0	1	0.9965	3.3646	no	yes	yes	no
X	47918103	*ZNF630*	nonsyn	A	C	C	W	576	0	1	0.9819	0.2213	no	no	yes	no
X	84329375	*APOOL*	nonsyn	G	T	K	N	232	0.31			0.0087	yes	no	no	no
X	99661761	*PCDH19*	nonsyn	C	T	R	H	612	0.02	0.99	0.9989	0.0276	no	yes	yes	no
X	138880854	*ATP11C*	nonsyn	A	C	N	K	256	0	1	0.9987	1.0978	no	no	yes	no
X	151908787	*CSAG1*	nonsyn	C	A	P	H	9	0	0.79	0.8333	6.6042	yes	no	no	no
X	151935493	*MAGEA3*	nonsyn	A	C	L	R	225	0	1	0.9731	0.6124	yes	no	no	no

Table S2 F: SNV table patient 2 (damaging or conserved, additional SNVs to Suppl. Table 2D)

chr	pos	gene	effect	ref nt	observ. nt	ref aa	observ. aa	pos aa	Sift	PolyPhen	PhyloP	ECS	g-st	e-st	e-bs	cosmic
1	16343573	*HSPB7*	nonsyn	T	C	E	G	110	0.00	0.64	0.9968	0.0272	no	no	yes	no
1	21926027	*RAP1GAP*	nonsyn	A	G	V	A	643	1.00	0.47	0.9957	11.463	no	no	yes	no
1	22047604	*USP48*	nonsyn	G	T	L	I	607	0.05	0.00	0.9996	0.0346	yes	no	no	no
1	22329501	*CELA3A*	nonsyn	G	T	G	C	17	0.00	0.72	0.9975	106.867	no	no	yes	no
1	57185921	*C1orf168*	nonsyn	T	G	S	R	686	0.07	0.56	0.9687	0.4269	no	yes	yes	no

1	91859934	*HFM1*	nonsyn	T	G	L	F	70	0.00	0.16	0.9389	19.611	no	no	yes	no
1	109801069	*CELSR2*	nonsyn	T	C	V	A	1109	0.61	0.45	0.9964	0.7822	no	no	yes	no
1	158151974	*CD1D*	nonsyn	T	G	L	V	161	0.29	0.00	0.0001	0.0579	no	no	yes	no
1	175048706	*TNN*	nonsyn	T	C	V	A	216	0.33	0.55	0.8329	46.155	no	no	yes	no
1	196367787	*KCNT2*	nonsyn	A	C	I	M	400	0.00	0.68	0.9767	0.0143	no	no	yes	no
1	196887442	*CFHR4*	nonsyn	T	A	F	Y	301	0.00	0.39	0.9871	56.184	no	no	yes	no
2	14774302	*FAM84A*	nonsyn	A	C	S	R	67	0.05	0.00	0.9982	0.0000	no	no	yes	no
2	86333449	*PTCD3*	nonsyn	T	A	L	M	27	0.00	0.00	0.0070	0.1793	no	no	yes	no
2	107041568	*RGPD3*	nonsyn	A	C	I	S	952	0.02			13.425	no	no	yes	no
2	116599859	*DPP10*	nonsyn	T	G	F	V	770	0.00	0.97	0.9985	19.456	no	no	yes	no
2	196891498	*DNAH7*	nonsyn	A	G	V	A	218	0.01	0.81	0.9991	19.891	no	yes	no	no
2	215813869	*ABCA12*	nonsyn	A	C	F	C	1968	0.00	1.00	0.9764	11.316	no	no	yes	no
2	225661686	*DOCK10*	nonsyn	A	C	L	V	1608	0.00			0.5502	no	no	yes	no
3	48685833	*CELSR3*	nonsyn	A	C	L	R	2280	0.00	0.79	0.9964	0.4052	no	no	yes	no
4	876583	*GAK*	nonsyn	G	A	R	W	477	0.00	1.00	0.0843	0.5924	no	no	yes	no
4	13571747	*BOD1L*	nonsyn	T	G	K	T	3015	0.00	0.99	0.9968	13.509	no	no	yes	no
5	141694306	*SPRY4*	nonsyn	T	A	Q	L	123	0.64	0.95	0.9982	0.0349	no	no	yes	no
6	33137191	*COL11A2*	nonsyn	G	C	P	R	1149	0.04	0.75	0.9989	0.7828	no	no	yes	no
6	117687277	*ROS1*	nonsyn	T	C	Q	R	925	0.21	0.92	0.8620	15.183	no	no	yes	yes
7	106938626	*COG5*	nonsyn	T	G	D	A	456	0.01	0.83	0.8933	0.9487	yes	no	no	no
7	141536919	*PRSS37*	nonsyn	A	C	I	S	187	0.86	0.99	0.9993	41.145	no	no	yes	no
8	65509363	*CYP7B1*	nonsyn	A	C	F	V	453	0.06	0.47	0.9767	0.3939	no	no	yes	no
9	125512506	*OR1L6*	nonsyn	T	C	I	T	127	0.00	0.98	0.9969	380.865	no	no	yes	no
10	1263020	*ADARB2*	nonsyn	T	C	H	R	518	0.02	1.00	0.9970	0.7311	no	no	yes	no
10	118645926	*KIAA1598*	nonsyn	C	A	G	C	609	0.02			0.0199	no	no	yes	yes
10	135347299	*CYP2E1*	nonsyn	A	G	I	V	289	0.03	0.00	0.0142	0.6131	yes	no	no	no
11	64663944	*ATG2A*	nonsyn	T	C	Q	R	1806	0.10	0.99	0.9942	15.500	no	no	yes	no
11	118518752	*PHLDB1*	nonsyn	T	C	V	A	1158	0.00	0.57	0.9976	0.2071	no	no	yes	no
11	118986890	*C2CD2L*	nonsyn	T	A	F	Y	683	0.03	0.99	0.9980	0.2956	no	no	yes	no

12	40012871	*ABCD2*	nonsyn	C	T	A	T	183	0.01	0.74	0.9980	0.0799	yes	no	no	no
14	25043464	*CTSG*	nonsyn	T	G	K	T	194	0.00	0.98	0.9676	15.316	no	yes	yes	no
14	37132597	*PAX9*	nonsyn	C	T	T	M	167	0.02	0.46	0.9984	11.336	yes	no	no	no
16	150473	*NPRL3*	nonsyn	T	C	S	G	43	0.00			0.1839	no	no	yes	no
16	31090892	*ZNF646*	nonsyn	G	A	V	I	1083	0.35	0.05	0.9997	18.997	no	no	yes	no
16	67709818	*GFOD2*	nonsyn	A	G	M	T	133	0.05	0.82	0.9981	0.0107	no	yes	yes	no
16	68023265	*DPEP2*	nonsyn	T	G	K	T	344	0.03	0.00	0.9742	44.821	no	yes	yes	no
16	84224888	*ADAD2*	nonsyn	C	G	R	G	18	0.00	0.99	0.9674	42.752	no	no	yes	no
16	88713569	*CYBA*	nonsyn	A	T	F	Y	48	0.04	0.87	0.9957	162.433	no	no	yes	no
17	14205307	*HS3ST3B1*	nonsyn	C	T	R	C	158	0.00	0.74	0.9978	0.2017	yes	no	no	no
17	36499576	*GPR179*	nonsyn	G	A	R	C	33	0.00	1.00	0.9909	0.6524	no	no	yes	no
17	36895869	*PCGF2*	nonsyn	A	G	V	A	60	0.00	0.99	0.9652	0.0237	no	no	yes	no
17	37374365	*STAC2*	nonsyn	T	C	N	S	51	0.00	0.99	0.9977	0.0257	no	yes	yes	no
17	38508279	*RARA*	nonsyn	A	C	Q	P	99	0.00	0.37	0.9966	0.0206	no	no	yes	yes
17	48277129	*COL1A1*	nonsyn	A	G	C	R	95	0.00	0.79	0.9983	10.697	no	no	yes	yes
18	3879864	*DLGAP1*	stoploss	T	C	X	G	69		0.98	0.9981	0.6117	no	no	yes	no
18	9887707	*TXNDC2*	nonsyn	A	G	T	A	411		0.79	0.2339	79.892	no	no	yes	no
19	9578567	*ZNF560*	nonsyn	T	A	E	D	352	0.13	0.87	0.7827	0.2806	no	no	yes	no
19	11568960	*ELAVL3*	nonsyn	G	A	T	M	210	0.37	0.00	0.9836	0.0012	no	no	yes	no
19	36342206	*NPHS1*	nonsyn	C	A	G	W	119	0.00	0.94	0.9432	12.396	no	no	yes	no
19	39871319	*SAMD4B*	nonsyn	G	C	R	P	581	0.00	1.00	0.9992	0.0188	no	no	yes	no
19	55241094	*KIR3DL3*	nonsyn	T	C	L	P	264	0.02	0.31	0.0186	16.339	no	no	yes	no
22	40800438	*SGSM3*	nonsyn	C	G	I	M	115	0.00	0.09	0.8535	0.1747	no	no	yes	no
X	12736568	*FRMPD4*	nonsyn	T	C	F	S	1208	0.00	0.07	0.9979	0.0212	no	no	yes	no

Table S3 A: Small InDels patient 1

chr	start	end	gene	type	ref nt	observed nt	ref aa	aa pos	called in exome?	called in genome?	cosmic
1	1560788	1560788	*MIB2*	frameshift insertion	-	G	W	332	uncovered	yes	no
1	10725600	10725600	*CASZ1*	frameshift insertion	-	G	P	15	uncovered	yes	no
1	27088787	27088787	*ARID1A*	frameshift insertion	-	G	Q	799	no	yes	yes
1	39833833	39833833	*MACF1*	frameshift deletion	A	-	E	2702	no	yes	no
1	40366751	40366751	*MYCL1*	frameshift insertion	-	G	R	119	uncovered	yes	no
1	47904569	47904569	*FOXD2*	frameshift insertion	-	G	G	254	uncovered	yes	no
1	50884762	50884762	*DMRTA2*	frameshift insertion	-	C	L	402	uncovered	yes	no
1	59156071	59156071	*MYSM1*	frameshift deletion	T	-	K	79	yes	yes	no
1	92643415	92643415	*KIAA1107*	frameshift deletion	A	-	K	363	uncovered	yes	no
1	156255317	156255317	*TMEM79*	frameshift insertion	-	C	E	100	yes	yes	no
1	214814551	214814554	*CENPF*	frameshift deletion	AAAT	-		957	yes	yes	no
1	225528370	225528370	*DNAH14*	frameshift insertion	-	A	E	3456	uncovered	yes	no
1	235345419	235345419	*ARID4B*	frameshift deletion	T	-	T	853	yes	yes	no
1	249141827	249141827	*ZNF672*	frameshift insertion	-	C	R	118	uncovered	yes	no
2	17698737	17698737	*RAD51AP2*	frameshift deletion	T	-	T	316	no	yes	no
2	24300578	24300580	*TP53I3*	nonframeshift deletion	GGA	-		224	yes	yes	no
2	73520690	73520690	*EGR4*	frameshift deletion	C	-	G	22	uncovered	yes	no
2	97824365	97824365	*ANKRD36*	frameshift insertion	-	T	N	454	uncovered	yes	no
2	109421454	109421454	*CCDC138*	frameshift insertion	-	A	L	282	no	yes	no
2	152320541	152320541	*RIF1*	frameshift deletion	A	-	K	1503	yes	yes	no
2	166011130	166011130	*SCN3A*	frameshift deletion	A	-	F	404	yes	yes	no
2	178257585	178257585	*AGPS*	frameshift insertion	-	G	A	23	uncovered	yes	no
2	186662103	186662103	*FSIP2*	frameshift deletion	A	-	K	3503	uncovered	yes	no
2	217559252	217559252	*IGFBP5*	frameshift deletion	G	-	R	83	uncovered	yes	no
2	219757717	219757717	*WNT10A*	frameshift insertion	-	C	G	326	uncovered	yes	no
2	231738163	231738163	*ITM2C*	frameshift deletion	G	-	R	51	no	yes	no

2	242626190	242626190	*DTYMK*	frameshift insertion	-	C	A	3	uncovered	yes	no
3	45267441	45267441	*TMEM158*	frameshift insertion	-	G	G	27	uncovered	yes	no
3	50647874	50647875	*CISH*	frameshift deletion	TG	-		13324	uncovered	yes	no
3	126194513	126194513	*ZXDC*	frameshift insertion	-	G	A	66	uncovered	yes	no
3	184039592	184039592	*EIF4G1*	frameshift deletion	C	-	A	211	yes	yes	no
3	194991598	194991598	*C3orf21*	frameshift insertion	-	G	A	64	uncovered	yes	no
4	15005749	15005749	*CPEB2*	frameshift insertion	-	G	G	484	uncovered	yes	no
4	25278792	25278792	*PI4K2B*	frameshift deletion	T	-	F	477	yes	yes	no
4	42153875	42153875	*BEND4*	frameshift insertion	-	G	A	96	uncovered	yes	no
4	71508472	71508472	*ENAM*	frameshift deletion	A	-	P	443	no	yes	no
4	109748336	109748336	*COL25A1*	frameshift deletion	T	-	K	573	no	yes	no
4	142643137	142643137	*IL15*	frameshift insertion	-	A	L	57	yes	yes	no
4	164466808	164466808	*03.01.2017*	frameshift deletion	A	-	W	154	yes	yes	no
5	60628709	60628709	*ZSWIM6*	frameshift insertion	-	G	R	204	uncovered	yes	no
5	73981222	73981222	*HEXB*	frameshift insertion	-	G	P	46	uncovered	yes	no
5	145883505	145883506	*TCERG1*	frameshift deletion	GA	-		868	yes	yes	no
5	169309816	169309816	*FAM196B*	frameshift insertion	-	G	T	363	uncovered	yes	no
5	169677853	169677853	*LCP2*	frameshift deletion	T	-	T	454	yes	yes	no
6	22570251	22570251	*HDGFL1*	frameshift deletion	G	-	A	149	uncovered	yes	no
6	25491971	25491971	*LRRC16A*	frameshift deletion	T	-	N	359	uncovered	yes	no
6	30122095	30122096	*TRIM10*	frameshift deletion	GT	-		366	yes	yes	no
6	31868562	31868562	*ZBTB12*	frameshift deletion	G	-	P	174	no	yes	no
6	42073643	42073643	*C6orf132*	frameshift insertion	-	G	P	669	uncovered	yes	no
6	138751899	138751899	*NHSL1*	frameshift insertion	-	G	I	1199	uncovered	yes	no
6	150263278	150263278	*ULBP2*	frameshift insertion	-	G	R	24	uncovered	yes	no
6	166721385	166721385	*PRR18*	frameshift insertion	-	C	S	82	uncovered	yes	no
7	2394615	2394615	*EIF3B*	frameshift insertion	-	C	G	20	uncovered	yes	no
7	6662529	6662529	*ZNF853*	frameshift insertion	-	G	P	636	uncovered	yes	no
7	27170236	27170236	*HOXA4*	frameshift insertion	-	C	G	39	uncovered	yes	no

7	44268438	44268438	*CAMK2B*	frameshift insertion	-	G	P	475	uncovered	yes	no
7	138916512	138916512	*UBN2*	frameshift insertion	-	C	E	94	uncovered	yes	no
7	140482927	140482927	*BRAF*	frameshift deletion	G	-	P	403	yes	no	yes
7	141170468	141170468	*LOC100507421*	frameshift deletion	G	-	W	256	uncovered	yes	no
8	11566247	11566249	*GATA4*	nonframeshift deletion	CCT	-		142	uncovered	yes	no
8	38645217	38645217	*TACC1*	frameshift insertion	-	G	P	39	uncovered	yes	no
8	54793598	54793598	*RGS20*	frameshift insertion	-	G	E	8	uncovered	yes	no
8	68950480	68950480	*PREX2*	frameshift insertion	-	T	V	264	yes	no	yes
8	145535689	145535689	*HSF1*	frameshift deletion	C	-	P	301	uncovered	yes	no
9	13217182	13217182	*MPDZ*	frameshift insertion	-	T	L	400	uncovered	yes	no
9	125330322	125330324	*OR1L8*	nonframeshift deletion	CAG	-		145	yes	yes	no
9	136342177	136342177	*SLC2A6*	frameshift insertion	-	C	L	148	uncovered	yes	no
9	139259595	139259595	*CARD9*	frameshift deletion	G	-	H	478	uncovered	yes	no
9	139564768	139564768	*EGFL7*	frameshift deletion	C	-	A	186	uncovered	yes	no
10	21101841	21101841	*NEBL*	frameshift insertion	-	T	T	129	yes	no	no
10	23728536	23728536	*OTUD1*	frameshift insertion	-	G	T	50	uncovered	yes	no
10	88423518	88423518	*OPN4*	frameshift deletion	C	-	P	453	uncovered	yes	no
10	99400629	99400629	*PI4K2A*	frameshift insertion	-	C	S	44	uncovered	yes	no
10	101295273	101295273	*NKX2-3*	frameshift insertion	-	G	A	297	uncovered	yes	no
10	102746683	102746685	*MRPL43*	nonframeshift deletion	CTC	-		96	uncovered	yes	no
10	102987157	102987157	*LBX1*	frameshift deletion	G	-	P	239	uncovered	yes	no
10	103990420	103990420	*PITX3*	frameshift insertion	-	G	Y	254	uncovered	yes	no
10	104182716	104182716	*FBXL15*	frameshift insertion	-	G	A	290	uncovered	yes	no
10	121411254	121411254	*BAG3*	frameshift deletion	C	-	P	23	uncovered	yes	no
10	127462683	127462683	*MMP21*	frameshift deletion	G	-	P	138	uncovered	yes	no
11	2181203	2181203	*INS*	frameshift deletion	C	-	G	71	uncovered	yes	no
11	33721959	33721961	*C11orf91*	nonframeshift deletion	AGA	-		109	uncovered	yes	no
11	47202199	47202199	*PACSIN3*	frameshift deletion	A	-	F	85	uncovered	yes	no
11	58346929	58346929	*ZFP91*	frameshift insertion	-	C	A	59	uncovered	yes	no

11	65403681	65403681	*PCNXL3*	frameshift insertion	-	G	G	1832	uncovered	yes	no
11	66307267	66307267	*ZDHHC24*	frameshift insertion	-	AAGG	L	196	uncovered	yes	no
11	67262953	67262953	*PITPNM1*	frameshift deletion	G	-	P	812	uncovered	yes	no
11	71951160	71951160	*PHOX2A*	frameshift insertion	-	C	A	163	uncovered	yes	no
11	75379136	75379136	*MAP6*	frameshift insertion	-	C	R	93	uncovered	yes	no
11	87013374	87013374	*TMEM135*	frameshift deletion	T	-	S	174	yes	yes	no
11	92087750	92087750	*FAT3*	frameshift insertion	-	A	A	824	yes	yes	no
11	93428779	93428779	*KIAA1731*	frameshift deletion	A	-	R	550	uncovered	yes	no
11	108256684	108256684	*C11orf65*	frameshift insertion	-	T	K	250	no	yes	no
11	122848460	122848460	*BSX*	frameshift insertion	-	C	A	200	uncovered	yes	no
12	19592890	19592890	*AEBP2*	frameshift insertion	-	C	S	86	uncovered	yes	no
12	48577960	48577960	*C12orf68*	frameshift deletion	C	-	P	19	uncovered	yes	no
12	50190327	50190327	*NCKAP5L*	frameshift deletion	G	-	P	439	uncovered	yes	no
12	54332886	54332886	*HOXC13*	frameshift insertion	-	C	A	66	uncovered	yes	yes
12	56827355	56827355	*TIMELESS*	frameshift insertion	-	A	L	111	yes	no	no
12	58149402	58149402	*03.06.2017*	nonframeshift insertion	-	CGG	R	31	uncovered	yes	no
12	62954511	62954511	*MON2*	frameshift deletion	A	-	E	1217	yes	yes	no
12	65563984	65563984	*LEMD3*	frameshift insertion	-	G	A	203	uncovered	yes	no
12	80175596	80175596	*PPP1R12A*	frameshift insertion	-	T	R	898	uncovered	yes	no
12	106532389	106532389	*NUAK1*	frameshift insertion	-	G	L	15	uncovered	yes	no
12	110941673	110941673	*RAD9B*	frameshift deletion	A	-	K	37	no	yes	no
13	32885712	32885712	*ZAR1L*	frameshift deletion	G	-	P	117	uncovered	yes	no
13	110435612	110435612	*IRS2*	frameshift insertion	-	G	R	930	uncovered	yes	no
13	112722529	112722529	*SOX1*	frameshift insertion	-	C	G	186	uncovered	yes	no
14	61190643	61190643	*SIX4*	frameshift insertion	-	G	P	50	uncovered	yes	no
14	75230626	75230626	*YLPM1*	frameshift deletion	C	-	S	145	uncovered	yes	no
14	77744769	77744769	*POMT2*	frameshift insertion	-	C	G	705	uncovered	yes	no
14	101005508	101005508	*BEGAIN*	frameshift insertion	-	G	S	194	uncovered	yes	no
14	104638177	104638179	*KIF26A*	nonframeshift deletion	GGT	-		411	uncovered	yes	no

15	34444996	34444996	*C15orf29*	frameshift deletion	A	-	S	145	yes	yes	no
15	41687234	41687234	*NDUFAF1*	frameshift deletion	A	-	F	194	yes	yes	no
15	42174159	42174159	*SPTBN5*	frameshift insertion	-	C	A	776	uncovered	yes	no
15	63414257	63414257	*LACTB*	frameshift insertion	-	C	A	63	uncovered	yes	no
15	68119568	68119568	*SKOR1*	frameshift insertion	-	C	A	424	uncovered	yes	no
15	72612182	72612182	*CELF6*	frameshift insertion	-	G	A	12	uncovered	yes	no
15	73615063	73615063	*HCN4*	frameshift deletion	C	-	G	1124	uncovered	yes	no
15	73615394	73615394	*HCN4*	frameshift deletion	C	-	A	1014	uncovered	yes	no
15	74726122	74726122	*SEMA7A*	frameshift insertion	-	C	G	46	uncovered	yes	no
15	75942736	75942736	*SNX33*	frameshift deletion	C	-	D	431	no	yes	no
16	1245562	1245562	*CACNA1H*	frameshift insertion	-	G	A	181	uncovered	yes	no
16	1401980	1401980	*GNPTG*	frameshift insertion	-	G	L	5	uncovered	yes	no
16	20810178	20810178	*ERI2*	frameshift deletion	T	-	N	315	uncovered	yes	no
16	20844363	20844363	*LOC81691*	frameshift deletion	T	-	L	434	yes	yes	no
16	27506189	27506189	*GTF3C1*	frameshift insertion	-	G	P	891	yes	no	no
16	30021389	30021389	*DOC2A*	frameshift insertion	-	CC	A	52	uncovered	yes	no
16	50187728	50187728	*PAPD5*	frameshift insertion	-	C	T	51	uncovered	yes	no
16	67695546	67695546	*PARD6A*	frameshift insertion	-	C	G	84	uncovered	yes	no
16	72993830	72993830	*ZFHX3*	frameshift insertion	-	C	S	72	uncovered	yes	yes
16	75682105	75682105	*TERF2IP*	frameshift insertion	-	C	T	109	uncovered	yes	no
16	87448918	87448918	*ZCCHC14*	frameshift insertion	-	C	A	343	yes	yes	no
16	88495435	88495435	*ZNF469*	frameshift insertion	-	G	G	519	uncovered	yes	no
16	89349641	89349641	*ANKRD11*	frameshift deletion	T	-	K	1103	yes	yes	no
16	89753118	89753120	*CDK10*	frameshift deletion	CAT	-		10	uncovered	yes	no
17	2298441	2298441	*MNT*	frameshift deletion	G	-	P	127	uncovered	yes	no
17	4693224	4693224	*GLTPD2*	frameshift deletion	C	-	A	170	uncovered	yes	no
17	10600660	10600660	*SCO1*	frameshift insertion	-	G	S	55	uncovered	yes	no
17	18087645	18087645	*ALKBH5*	frameshift insertion	-	C	A	30	uncovered	yes	no
17	20108263	20108263	*SPECC1*	frameshift deletion	A	-	K	220	no	yes	yes

17	46985900	46985900	*UBE2Z*	frameshift insertion	-	G	A	12	uncovered	yes	no
17	46986017	46986018	*UBE2Z*	frameshift deletion	CA	-		51	uncovered	yes	no
17	47297238	47297238	*ABI3*	frameshift insertion	-	G	P	183	uncovered	yes	no
17	48624635	48624635	*SPATA20*	frameshift insertion	-	G	A	22	uncovered	yes	no
17	76968105	76968105	*LGALS3BP*	frameshift deletion	C	-	G	437	yes	yes	no
17	79425361	79425361	*BAHCC1*	frameshift insertion	-	C	A	1683	uncovered	yes	no
17	79425400	79425400	*BAHCC1*	frameshift insertion	-	C	A	1696	uncovered	yes	no
19	577814	577814	*BSG*	frameshift deletion	G	-	V	36	uncovered	yes	no
19	1465537	1465537	*APC2*	frameshift insertion	-	C	G	746	uncovered	yes	no
19	2251182	2251182	*AMH*	frameshift deletion	C	-	V	303	uncovered	yes	no
19	4175089	4175089	*SIRT6*	frameshift insertion	-	G	L	198	uncovered	yes	no
19	4543990	4543990	*SEMA6B*	frameshift insertion	-	G	A	764	uncovered	yes	no
19	10121036	10121036	*COL5A3*	frameshift insertion	-	G	Q	9	uncovered	yes	no
19	14201203	14201203	*SAMD1*	frameshift insertion	-	G	P	10	uncovered	yes	no
19	19006845	19006845	*CERS1*	frameshift insertion	-	C	P	13	uncovered	yes	no
19	19654762	19654762	*CILP2*	frameshift deletion	C	-	P	470	no	yes	no
19	36120066	36120066	*RBM42*	frameshift insertion	-	G	A	4	uncovered	yes	no
19	36223970	36223970	*MLL4*	frameshift insertion	-	T	V	2174	uncovered	yes	no
19	36359544	36359544	*APLP1*	frameshift insertion	-	C	G	2	uncovered	yes	no
19	39226910	39226910	*CAPN12*	frameshift insertion	-	C	L	475	uncovered	yes	no
19	41699266	41699266	*CYP2S1*	frameshift deletion	C	-	P	33	uncovered	yes	no
19	44118005	44118006	*SRRM5*	frameshift deletion	GA	-		578	uncovered	yes	no
19	45899436	45899436	*PPP1R13L*	frameshift deletion	C	-	G	298	uncovered	yes	no
19	48949367	48949367	*GRWD1*	frameshift insertion	-	G	P	35	uncovered	yes	no
19	50161631	50161631	*SCAF1*	frameshift insertion	-	G	P	1305	uncovered	yes	no
19	54659494	54659494	*LENG1*	frameshift insertion	-	C	R	254	uncovered	yes	no
19	54974667	54974667	*LENG9*	frameshift insertion	-	G	A	37	uncovered	yes	no
19	55998075	55998075	*NAT14*	frameshift insertion	-	C	V	125	uncovered	yes	no
19	56011445	56011445	*SSC5D*	frameshift deletion	C	-	S	656	uncovered	yes	no

20	278509	278509	*ZCCHC3*	frameshift insertion	-	G	R	94	uncovered	yes	no
20	5987071	5987071	*CRLS1*	frameshift insertion	-	G	L	60	uncovered	yes	no
20	21695239	21695239	*PAX1*	frameshift insertion	-	G	Q	468	uncovered	yes	no
20	35064745	35064745	*DLGAP4*	frameshift deletion	C	-	N	411	uncovered	yes	no
20	35414897	35414897	*KIAA0889*	frameshift deletion	G	-	P	1659	uncovered	yes	no
20	42143414	42143414	*L3MBTL1*	frameshift insertion	-	G	A	77	uncovered	yes	no
20	57429638	57429640	*GNAS*	nonframeshift deletion	GAT	-		377	uncovered	yes	yes
20	58514841	58514841	*PPP1R3D*	frameshift insertion	-	G	R	49	uncovered	yes	no
20	62200526	62200528	*PRIC285*	nonframeshift deletion	CCT	-		354	uncovered	yes	no
21	34443272	34443272	*OLIG1*	frameshift insertion	-	G	G	240	uncovered	yes	no
21	46897723	46897723	*COL18A1*	frameshift deletion	C	-	G	535	uncovered	yes	no
22	17601541	17601541	*CECR6*	frameshift insertion	-	C	G	159	uncovered	yes	no
22	17601574	17601574	*CECR6*	frameshift insertion	-	C	G	148	uncovered	yes	no
22	19511720	19511720	*CLDN5*	frameshift deletion	C	-	G	105	uncovered	yes	no
22	22221701	22221701	*MAPK1*	frameshift insertion	-	C	G	10	uncovered	yes	yes
22	26902289	26902289	*TFIP11*	frameshift deletion	C	-	G	167	yes	yes	no
22	40058306	40058306	*CACNA1I*	frameshift insertion	-	C	A	1045	uncovered	yes	no
22	51133261	51133261	*SHANK3*	frameshift insertion	-	T	G	363	uncovered	yes	no
X	16965034	16965034	*REPS2*	frameshift insertion	-	GG	A	17	uncovered	yes	no
X	48814518	48814518	*OTUD5*	frameshift insertion	-	C	G	105	uncovered	yes	no
X	119048674	119048674	*AKAP14*	frameshift deletion	A	-	K	92	yes	no	no
X	128782641	128782641	*APLN*	frameshift insertion	-	C	R	66	uncovered	yes	no

Table S3 B: Small InDels patient 2

chr	start	end	gene	type	ref nt	observed nt	ref aa	aa pos	called in exome?	called in genome?	cosmic
1	38274248	38274248	*C1orf122*	frameshift insertion	-	G	G	27	uncovered	yes	no
2	9533687	9533687	*ASAP2*	frameshift insertion	-	C	K	820	uncovered	yes	no
2	42275911	42275911	*PKDCC*	frameshift insertion	-	G	L	191	uncovered	yes	no
2	101869561	101869561	*C2orf29*	frameshift insertion	-	G	S	45	uncovered	yes	no
2	102003876	102003876	*CREG2*	frameshift insertion	-	G	R	15	uncovered	yes	no
2	175201193	175201193	*SP9*	frameshift insertion	-	G	A	127	uncovered	yes	no
2	176957800	176957801	*HOXD13*	frameshift deletion	CA	-		61	uncovered	yes	yes
2	230578923	230578923	*DNER*	frameshift insertion	-	G	A	73	uncovered	yes	no
3	57199225	57199227	*IL17RD*	nonframeshift deletion	GGA	-		30	uncovered	yes	no
3	118621756	118621756	*IGSF11*	frameshift deletion	T	-	I	303	no	yes	no
3	147128069	147128069	*ZIC1*	frameshift insertion	-	T	A	57	uncovered	yes	no
6	42893110	42893111	*PTCRA*	frameshift deletion	TG	-		179	uncovered	yes	no
6	110679218	110679218	*C6orf186*	frameshift deletion	G	-	G	86	uncovered	yes	no
6	166721600	166721600	*PRR18*	frameshift deletion	C	-	A	11	uncovered	yes	no
7	65447139	65447139	*GUSB*	frameshift deletion	G	-	A	11	uncovered	yes	no
7	155532530	155532531	*RBM33*	frameshift deletion	AG	-		620	uncovered	yes	no
8	25224393	25224398	*DOCK5*	nonframeshift deletion	ACAATT	-	na	1044	yes	yes	no
8	37756930	37756930	*RAB11FIP1*	frameshift insertion	-	C	G	10	uncovered	yes	no
8	145735004	145735004	*MFSD3*	frameshift insertion	-	C	G	96	uncovered	yes	no
10	48438611	48438611	*GDF10*	frameshift insertion	-	G	S	34	uncovered	yes	no
11	1092475	1092475	*MUC2*	frameshift insertion	-	AT	P	1432	uncovered	yes	no
11	64565113	64565113	*MAP4K2*	frameshift deletion	T	-	E	374	uncovered	yes	no
15	23891258	23891258	*MAGEL2*	frameshift insertion	-	G	T	544	uncovered	yes	no
15	68119471	68119471	*SKOR1*	frameshift insertion	-	G	G	391	uncovered	yes	no
16	29818333	29818333	*MAZ*	frameshift insertion	-	G	P	76	uncovered	yes	no
17	636375	636375	*FAM57A*	frameshift insertion	-	C	S	54	uncovered	yes	no
17	4462251	4462251	*GGT6*	frameshift insertion	-	G	A	149	uncovered	yes	no

19	40317538	40317538	*DYRK1B*	frameshift insertion	-	C	G	395	uncovered	yes	no
20	25062560	25062560	*VSX1*	frameshift insertion	-	C	P	58	uncovered	yes	no
20	57429638	57429640	*GNAS*	nonframeshift deletion	GAT	-		377	uncovered	yes	yes
20	62373884	62373884	*SLC2A4RG*	frameshift insertion	-	C	R	292	uncovered	yes	no
X	11682758	11682758	*ARHGAP6*	frameshift insertion	-	G	R	64	uncovered	yes	no

Table S4 A: Inversions patient No. 1

chromosome	start	end	gene
chr4	71878175	71955584	*DCK*
chr5	147553039	147554779	*SPINK14*
chr7	129066570	129091085	*AHCYL2, FAM40B*
chr9	201453	68434529	*LOC642236*
chr14	91825622	91856306	*CCDC88C*
chr17	36350387	36406170	*LOC440434*
chr18	66398498	66399176	*CCDC102B*

Table S4 B: Inversions patient No. 2

chromosome	start	end	gene
chr1	33107241	33108379	*ZBTB8OS*
chr1	46026619	46056013	*AKR1A1, NASP*
chr1	58581661	58592142	*DAB1*
chr1	119569249	119600947	*WARS2*
chr3	37553372	37553456	*ITGA9*
chr3	106918402	106919564	*LOC100302640*
chr4	66275067	66348582	*EPHA5*
chr4	79031136	79035783	*FRAS1*
chr5	43213865	43213953	*NIM1*
chr5	147553039	147554616	*SPINK14*
chr6	57403534	57410135	*PRIM2*
chr6	73042386	73047307	*RIMS1*
chr6	74193716	74193781	*MTO1*
chr7	73531412	73534012	*LIMK1*
chr7	83184322	83191781	*SEMA3E*

chr10	78801102	78882773	*KCNMA1*
chr10	87963306	87967672	*GRID1*
chr11	47505799	47505871	*CELF1*
chr11	72702282	72702339	*FCHSD2*
chr12	25327274	25327342	*CASC1*
chr12	25718424	25749850	*IFLTD1*
chr12	66321678	66321734	*HMGA2*
chr12	71251513	71251579	*PTPRR*
chr12	78394308	78394758	*NAV3*
chr13	28815776	28815852	*PAN3*
chr14	37771227	37771306	*MIPOL1*
chr17	78727180	78727267	*RPTOR*
chr18	32588783	32588873	*MAPRE2*
chr18	66572493	66578708	*CCDC102B*

Table S4 C: Interchromosomal translocations patient No. 1

**breakpoint 1**		**breakpoint 2**		
**chromosome**	**position**	**chromosome**	**position**	**genes**
chr1	38432782	chr6	28863596	*SF3A3*
chr1	168025482	chr19	24033171	*DCAF6*
chr2	133012697	chr12	20704282	*ANKRD30BL*
chr3	196625679	chr10	42596810	*SENP5*
chr5	58988375	chr7	80801716	*PDE4D*
chr5	68499842	chrX	53054452	*CENPH*
chr6	24684231	chr22	32928263	*ACOT13*
chr6	144120812	chr7	153001941	*PHACTR2*
chr7	81789152	chr10	60902558	*CACNA2D1*
chr7	87255610	chrX	97409704	*ABCB1*

chr10	68672793	chr16	59976458	*CTNNA3*
chr10	73519977	chr11	115199242	*C10orf54, CDH23*
chr12	56989998	chr15	39994382	*BAZ2A*

Table S4 D: Interchromosomal translocations patient No. 2

**breakpoint 1**		**breakpoint 2**		
**chromosome**	**Position**	**chromosome**	**position**	**genes**
chr1	24999672	chr8	128100486	*SRRM1*
chr5	21474750	chr6	58272103	*GUSBP1*
chr5	112669294	chr6	40177372	*MCC*
chr5	79074210	chr12	25780056	*CMYA5*
chr6	20112057	chr9	117857675	*MBOAT1*
chr7	111053279	chr12	108203016	*IMMP2L*
chr7	147472362	chr17	35072033	*CNTNAP2*
chr7	151970937	chr21	11058238	*MLL3*
chr8	52730378	chr11	38812394	*PCMTD1*
chr9	68429385	chr20	29632249	*LOC642236*
chr9	117857835	chr20	37093067	*TNC*
chr17	45264766	chr22	20062765	*CDC27*
chr19	19632268	chr22	16347238	*NDUFA13*
chr19	49810907	chrX	144519280	*SLC6A16*

Table S4 E: Deletions patient No. 1

chromosome	deletion start	deletion end	gene	# deletions	avg. deletion length
chr5	19847034	19867791	*CDH18*	2	23.00
chr5	21527908	21569501	*GUSBP1*	9	11.44
chr5	21855682	22715926	*CDH12*	4	10.50
chr5	40794615	40794979	*PRKAA1*	1	363.00
chr5	52172144	52232105	*ITGA1*	4	103.00
chr5	58277277	59541862	*PDE4D*	9	16.56
chr5	70853481	70853549	*BDP1*	1	67.00
chr5	75435663	75561971	*SV2C*	3	47.33
chr5	77004046	77062619	*TBCA*	2	160.50
chr5	78277726	78278046	*ARSB*	1	319.00
chr5	80039371	80039681	*MSH3*	1	309.00
chr5	80838449	81008438	*SSBP2*	2	140.50
chr5	88032012	88032343	*MEF2C*	1	330.00
chr5	89918620	90351220	*GPR98*	4	10.25
chr5	93757792	93916621	*KIAA0825*	2	174.00
chr5	101796603	101796650	*SLCO6A1*	1	46.00
chr5	110584044	110709132	*CAMK4*	4	85.50
chr5	115781960	115894458	*SEMA6A*	2	163.50
chr5	122701699	122727303	*CEP120*	3	25.67
chr5	136604692	136783317	*SPOCK1*	2	27.50
chr5	137022562	137023887	*KLHL3*	1	1324.00
chr5	147462155	147462472	*SPINK5*	1	316.00
chr5	149746191	149746518	*TCOF1*	1	326.00
chr5	152986428	152986521	*GRIA1*	1	92.00
chr5	154315917	154316003	*GEMIN5*	1	85.00
chr5	158592397	158592743	*RNF145*	1	345.00
chr5	170092722	170158907	*KCNIP1*	2	50.00

chr6	38498278	38498416	*BTBD9*	1	137.00
chr6	38720386	38932857	*DNAH8*	4	93.25
chr6	41953350	41995188	*CCND3*	2	162.50
chr6	45063196	45180261	*SUPT3H*	2	25.00
chr6	45927255	45967786	*CLIC5*	4	88.75
chr6	51736109	51904144	*PKHD1*	2	351.00
chr6	55337265	55408298	*HMGCLL1*	4	81.75
chr6	57207690	57489304	*PRIM2*	48	252.25
chr6	62410046	62835931	*KHDRBS2*	5	11.40
chr6	64794896	66290505	*EYS*	14	164.50
chr6	69648332	70043874	*BAI3*	5	19.40
chr6	74421253	74501003	*CD109*	3	107.00
chr6	76716172	76746584	*IMPG1*	2	50.50
chr6	89449444	89449487	*RNGTT*	1	42.00
chr6	91237575	91237897	*MAP3K7*	1	321.00
chr6	102030791	102357886	*GRIK2*	4	13.25
chr6	105567837	105568173	*BVES*	1	335.00
chr6	106966857	106986043	*AIM1*	4	86.50
chr6	108031342	108032403	*SCML4*	1	1060.00
chr6	116630476	116751133	*DSE*	2	163.50
chr6	123624457	123897619	*TRDN*	5	10.80
chr6	124234027	125119226	*NKAIN2*	6	34.17
chr6	134811194	134817663	*LOC154092*	2	168.50
chr6	137313693	137314187	*NHEG1*	1	493.00
chr6	143138010	143161521	*HIVEP2*	3	37.67
chr6	153029971	153033790	*MYCT1*	1	3818.00
chr6	154446915	154447238	*OPRM1*	1	322.00
chr6	157957840	157958148	*ZDHHC14*	1	307.00
chr6	158548266	158549067	*SERAC1*	1	800.00

chr6	161795638	163140940	*PARK2*	6	18.33
chr6	169905557	170038446	*WDR27*	2	210.00
chr7	69094157	70093358	*AUTS2*	4	25.75
chr7	70852212	71166844	*WBSCR17*	4	19.25
chr7	71398080	71828232	*CALN1*	7	13.43
chr7	77692552	78955527	*MAGI2*	15	44.00
chr7	81358337	81380822	*HGF*	2	141.50
chr7	81590035	81904509	*CACNA2D1*	4	13.50
chr7	83019539	83251598	*SEMA3E*	5	15.80
chr7	83653800	83724687	*SEMA3A*	4	110.75
chr7	86836614	86836924	*C7orf23*	1	309.00
chr7	93169987	93170105	*CALCR*	1	117.00
chr7	101521036	101911193	*CUX1*	3	16.33
chr7	102488704	102607726	*FBXL13*	3	117.67
chr7	103231015	103614941	*RELN*	6	62.83
chr7	104187384	104347403	*LHFPL3*	3	115.33
chr7	105733990	105734329	*SYPL1*	1	338.00
chr7	110536533	110543010	*IMMP2L*	2	256.50
chr7	113867592	114154152	*FOXP2*	5	14.80
chr7	120881096	120881435	*C7orf58*	1	338.00
chr7	121963925	122346024	*CADPS2*	7	52.86
chr7	126301958	126775701	*GRM8*	3	36.00
chr7	131813284	132279786	*PLXNA4*	4	13.25
chr7	134349999	134350423	*BPGM*	1	423.00
chr7	134475804	134490463	*CALD1*	2	22.00
chr7	136739075	136845672	*LOC349160*	3	105.33
chr7	144382749	144382898	*TPK1*	1	148.00
chr7	146174662	148076324	*CNTNAP2*	8	468.00
chr7	151397836	151516844	*PRKAG2*	4	11.00

chr7	152099668	152109219	*MLL3*	16	15.69
chr7	157870222	158266488	*PTPRN2*	7	17.29
chr7	158563013	158574681	*ESYT2*	2	87.00

Table S4 F: Deletions patient No. 2

chromosome	deletion start	deletion end	gene	# deletions	avg. deletion length
chr8	9636709	9638499	*TNKS*	1	1789.00
chr11	61107785	61108125	*DAK*	1	339.00
chr12	23741655	24460697	*SOX5*	3	100.67
chr12	129088970	129089092	*TMEM132C*	1	121.00

Table S5: Clinico-pathological characteristics of patients with a GNAS-mutation

Patient No.	Gender	Age	Tumor type	Tumor stage	MSI*	GNAS Exon 8	GNAS Exon 9
3	male	88	intestinal	pT2 N0 (0/15) M0 L0 V0 G2 R0	MSI-H	p.R201H c.602G>A	wt
4	male	65	intestinal	pT3 N3b (16/31) M0 L0 V1 G2 R0	MSS	p.R201C c.601C>T	wt
5	male	67	intestinal	pT1a N0 (0/10) M0 L0 V0 G1 R0	MSS	p.R201H c.602G>A	wt
6	male	82	intestinal	pT3 N2 (3/21) M0 L1 V1 G2 R0	MSI-H	p.R201H c.602G>A	wt
7	male	56	diffuse	pT4b N3b (23/23) M1 L1 V0 G3 R1	MSS	p.R201L c.602G>T	wt
8	male	73	diffuse	pT3 N2 (6/18) M0 L0 V0 G3 R0	n.a.	p.R201H c.602G>A	wt
9	male	55	unclassified	pT4b N0 (0/27) M0 L0 V0 G3 R0	MSI-H	p.R201H c.602G>A	wt

*MSI-H: highly microsatellite unstable; MSS: microsatellite stable; n.a.: not applicable

Table S6 Allele calls of failed validations (MSI tumor)

			allele counts WES patient 1 tumor (MSI)						allele counts WES patient 1 non-tumor						allele counts WGS patient 1 tumor (MSI)						allele counts WGS patient 1 non-tumor					
**chr.**	**position**	**gene**	**cov.**	**#A**	**#C**	**#G**	**#T**	**%allele**	**cov.**	**#A**	**#C**	**#G**	**#T**	**%allele**	**cov.**	**#A**	**#C**	**#G**	**#T**	**%allele**	**cov.**	**#A**	**#C**	**#G**	**#T**	**%allele**
11	47296590	*MADD*	43	0	1	9	33	20.93	42	0	0	1	41	2.38	42	0	0	0	42	0	53	0	2	0	51	3.77
12	54369186	*HOXC11*	12	0	8	4	0	33.33	21	0	19	2	0	9.52	36	0	36	0	0	0	84	0	84	0	0	0
9	36966598	*PAX5*	15	4	11	0	0	26.67	22	1	21	0	0	4.55	39	0	39	0	0	0	57	0	57	0	0	0
17	56439928	*RNF43*	13	9	0	4	0	30.77	16	15	0	1	0	6.25	46	43	2	1	0	4.35	54	45	4	5	0	9.26
14	70990319	*ADAM20*	49	6	43	0	0	12.24	47	3	43	0	1	6.38	47	0	47	0	0	0	85	0	85	0	0	0
4	38800365	*TLR1*	41	8	33	0	0	19.51	33	5	28	0	0	15.15	56	0	56	0	0	0	107	1	106	0	0	0.93
2	222428603	*EPHA4*	66	0	0	56	10	15.15	94	0	0	82	12	12.77	63	0	0	63	0	0	82	1	0	81	0	1.22
7	120614861	*ING3*	28	0	22	6	0	21.43	30	2	26	2	0	6.67	67	0	67	0	0	0	115	0	115	0	0	0

Table S7 Allele calls of failed validations (MSS tumor)

			allele counts WES patient 2 tumor (MSS)						allele counts WES patient 2 non-tumor						allele counts WGS patient 2 tumor (MSS)						allele counts WGS patient 2 non-tumor					
**chr.**	**position**	**gene**	**cov.**	**#A**	**#C**	**#G**	**#T**	**%allele**	**cov.**	**#A**	**#C**	**#G**	**#T**	**%allele**	**cov.**	**#A**	**#C**	**#G**	**#T**	**%allele**	**cov.**	**#A**	**#C**	**#G**	**#T**	**%allele**
12	54369186	*HOXC11*	19	0	16	3	0	15.79	23	0	22	1	0	4.35	28	1	27	0	0	3.57	24	0	24	0	0	0
9	36966598	*PAX5*	19	5	14	0	0	26.32	11	0	11	0	0	0	12	0	12	0	0	0	37	0	37	0	0	0
17	56439928	*RNF43*	26	19	0	7	0	26.92	14	14	0	0	0	0	16	14	0	2	0	12.5	37	31	4	2	0	10.81
14	70990319	*ADAM20*	52	11	39	0	2	21.15	54	5	46	1	2	9.26	41	0	41	0	0	0	55	0	55	0	0	0
4	38800365	*TLR1*	29	4	25	0	0	13.79	32	2	30	0	0	6.25	28	0	28	0	0	0	57	0	56	0	1	1.75
2	222428603	*EPHA4*	98	0	0	82	16	16.33	70	0	1	62	7	10	29	0	0	29	0	0	43	1	0	42	0	2.33
7	120614861	*ING3*	25	0	16	9	0	36	31	0	28	3	0	9.68	29	0	29	0	0	0	44	0	42	1	1	2.27

Table S8 Putative insert position with adjacent gene < 100kb

chromosome	range start	range end	closest gene	distance to closest gene
chr2	92103056	92103061	*ACTR3BP2*	26098
chr7	141743389	141743389	*MGAM*	intronic
chr7	57688147	57688147	*L37717*	10499
chr7	57760881	57760881	*L37717*	62077
chr7	57786314	57786314	*L37717*	87510
chr9	40407198	40407198	*CNTNAP3B*	91283
chr9	41897881	41897886	*BC019880/GLIDR*	50628
chr9	42084498	42084498	*LOC554249*	64085
chr9	44460316	44460321	*LOC103908605*	57056
chr9	45607139	45607139	*LOC100132167*	43576
chr9	46900844	46900849	*LOC103908605*	55685
chr16	33109610	33109613	abParts	intronic
chr20	26255314	26255315	*LOC284801*	65445

Table S9 Number of called somatic SNVs

sample	# called SNVs
MSI WGS	19784
MSS WGS	5116
MSI WES	965
MSS WES	2722
