# Supplementary material for: Interpreting whole genome and exome sequencing data of individual gastric cancer samples
Source: BMC Genomics. 2017 Jul 6;18:517. doi: 10.1186/s12864-017-3895-z (PMC5501078; doi:10.1186/s12864-017-3895-z)
Supplement: Supplementary file 3 — Supplemental results [52] (Additional file 1: Figure S1, Fig. 2, Additional file 1: Figure S15, Fig. 2, Additional file 1: Figure S15, Additional file 1: Figure S16, Additional file 1: Figure S17, Additional file 1: Figure S18, Additional file 1: Figure S19, Additional file 4: Methods, Additional file 1: Figure S20, Additional file 1: Figure S20 A + B, Additional file 1: Figure S20 C + D, Additional file 1: Figure S12 and Additional file 1: Figure S13). (DOCX 30 kb) [file 12864_2017_3895_MOESM3_ESM.docx]

**Supplemental results**

### SNV caller comparison

The SNV caller comparison was carried out for two sequencing approaches and technologies (Suppl. Figure 1). The whole exome data were produced with a Solid paired 50/35 v4 run. The whole genome data were based on the Illumina HiSeq 2000 technology. In the whole exome sequencing data the SNV caller GATK, DiBayes and Samtools were compared, while in the whole genome results GATK and Samtools were used. In the WES data the highest number of SNVs were called with DiBayes followed by GATK and Samtools (Figure 2). Between 42% and 49% of the variants were detected by all programs and additional ~20% by two caller. 28-36% were exclusively found with DiBayes (Suppl. Figure 15). In the WGS data Samtools found more variants than GATK (Figure 2), while the overlap was between 65% and 88% (Suppl. Figure 15).

In the next step the number of SNVs, which could be validated, were investigated each program. A SNV was defined as confirmed, if at least 20% of the reads sequenced with a second technology supported the variant. For this analysis exclusively positions were considered, which were covered with both sequencing approaches. The highest number of verified variants was observed for SNVs called with GATK. The lowest number could be validated for SNVs called with DiBayes (Suppl. Figure 16). The results were similar to a 10% read support threshold (data not shown).

Furthermore, it was investigated how many SNVs of all cross platform variants were called by the different programs. Three stringency levels were applied: The SNVs were called confirmed, if in the data of the second technology (i) 10%, (ii) 20% or (iii) 50% of the reads supported the variant. In the WES approach the program DiBayes detected the highest number of verified SNVs, while this was the case for Samtools in the WGS data (Suppl. Figure 17).

A comparison between strict filtered (detected with all callers) SNVs in the WES data (called with Samtools and GATK and DiBayes) and those detected in the WGS data (called with Samtools and GATK) is shown in supplementary figure 18.

**Comparison between NGS approaches**

The samples were sequenced with two NGS approaches: (i) whole exome sequencing on the Solid 4 and (ii) whole genome sequencing on the Illumina HiSeq 2000. To test the false positive and false negative rate, all exonic SNVs called with Samtools were investigated in all sample pairs (Suppl. Figure 19). Around 12% of the intragenic positions, at which a SNV was called in the WGS, were uncovered in the WES data. In the reverse direction this was for only two SNV positions in one sample the case. The number of SNVs, which were uncovered in the WES, was especially high for C>A, C>G as well as T>G base substitutions and low for T>C variants. Around 5% of WGS-SNVs at positions, which were also covered in the WES, could not be confirmed with a 5% allele support threshold. Like for the uncovered SNV positions, the false positive rate was in C>A, C>G and T>G SNVs higher and in T>C lower than expected. Vice versa, less than 0.7% of SNVs called in the WES data could not be confirmed with WGS. In comparison to the total number of called SNVs, this was especially high for T>A and T>G base substitutions.

**Exonic gene conservation score (ECS)**

The ECS is a simplified indicator score, which compares the length-corrected non-synonymous mutation rate within the exonic regions of the gene of interest to the average non-synonymous mutation rate over all exonic regions of the genome (see Suppl. Methods). Across all genes the ECS value was 1.52, while the average ECS of genes out of the cancer gene census list from the COSMIC database [52] (http://cancer.sanger.ac.uk) was 0.66. The score was applied as a simplified filter to interpret the variants from our two clinical samples. An extremely low ECS value was found for example in the following genes: *PCGF2* (0.023), *ZIC1* (0.0), *VHLL* (0.044), *INHBA* (0.016), *MTA2* (0.011), *BRAF* (0.076), *GNB2L1* (0.015), *JAK2* (0.081), *CDC25B* (0.0459) and *EXT2* (0.099).

To demonstrate the link between ECS and its relevance for cancer, the obtained score values were compared to (i) the associated cancer proliferation index (cPI) and (ii) the number of samples harboring a mutation with predicted functional relevance in cancer (Suppl. Figure 20) for each gene in the COSMIC data set. The first analysis demonstrated that a low ECS was associated with a negative cPI (potential tumor suppressor genes) or a positive cPI value (potential oncogenes), while genes with a neutral associated cPI value harbored a higher ECS score (Suppl. Figure 20 A+B). This was also confirmed by a one-tailed Wilcoxon rank sum test comparing genes having an absolute cPI value values larger or equal to 0.4 with genes having an absolute cPI smaller 0.4 (p=0.0008). The cPI values separated by tumor suppressor genes and oncogenes were 0.2545 and 8,698e-05, respectively. However, the non-significant p-value is probably a consequence of too low power. In a second test, we could show that genes with low ECS harbored more often mutations predicted as cancer-driver mutations by FATHMM (Hidden markov model for functional variants) (p=1.75e-07). This is depicted in Supplementary Figure 20 C+D.

**Comparison with known cancer-associated SNVs**

Predisposing germline alleles in the cancer-associated variants annotated in the databases GWAS, OMIM or HGMD (mostly comprising polygenic SNVs from GWAS, but also some monogenic rare variants for high penetrance familial cancer syndromes e.g. BRCA1 spectrum of variants) were compared with all somatic and germline variants called in the tumor samples. In total 301 | 296 known cancer-associated SNVs (germline or somatic) were detected in the tumor samples of patient 1 (MSI) | patient 2 (MSS) including *BRCA1*, *DCC*, *FLCN*, *LOX*, *RASSF1*, and *TERT* (all germline). Out of these genes, 9 | 12 were associated with GC affecting exonic regions of the following genes: *MUC1* (germline, MSI), *NOD2* (germline, MSI+MSS), *CCL22* (germline, MSS+MSI), *VCAN* (germline, MSI) and *PLCE1* (germline, MSS), and *TP53* (somatic, MSS). Out of the mentioned variants 142 | 141 (overlap = 101) were found in the GWAS catalog, 82 | 72 (overlap = 61) in the database OMIM and 135 | 137 (overlap = 89) in HGMD. Indeed, nearly all SNVs represented polymorphisms with a population frequency >5%, existed in the tumor as well as in the matching control sample (declared as germline) and were thus considered as potentially predisposing, but not necessarily causative.

**Potential germline load of variance**

To investigate additionally the potential germline load of variance, all variants (germline and somatic) of the tumour samples were compared with the variants of the samples from the 1000 Genomes Project. The MSI-tumor sample of patient 1 harbored in 33 GO terms more SNVs with predicted influence on the function of the gene than all individuals sequenced in the 1000 Genomes Project. 19 terms existed with more genes affected by a SNV with damaging prediction in the tumor sample than the maximum number in the 1000 Genomes Project (Suppl. Figure 12). In the MSS-sample of the second patient 30 GO terms on SNV level and 13 on gene level were found, which were more often affected in the tumor sample than in all individuals out of the 1000 Genomes Project (Suppl. Figure 13). GO terms consisting of exactly the same genes were merged and GO terms harboring only one gene excluded. Nine GO terms were shared between the MSI- and MSS-tumor samples including the functional interesting terms ‘nuclear migration along microfilament’, 'negative regulation of transposition', ‘negative regulation of viral reproduction’, and 'DNA cytosine deamination'.
