# Supplementary material for: Interpreting whole genome and exome sequencing data of individual gastric cancer samples
Source: BMC Genomics. 2017 Jul 6;18:517. doi: 10.1186/s12864-017-3895-z (PMC5501078; doi:10.1186/s12864-017-3895-z)
Supplement: Supplementary file 4 — Supplemental Materials and methods [18, 53–59] (Table 1, Additional file 2: Table S2C-F and S3, Additional file 2: Tables S3A and S3B). (DOCX 35 kb) [file 12864_2017_3895_MOESM4_ESM.docx]

**Supplemental Materials and methods**

**Gastric cancer cohort**

From the archive of the Institute of Pathology, University Hospital Kiel, we identified all Caucasian patients who had undergone either total or partial gastrectomy for adenocarcinomas of the stomach or oesophago-gastric junction between 1997 and 2009. The following patient characteristics were retrieved: type of surgery, age at diagnosis, gender, tumor localization and tumor size, tumor type, tumor grade, depth of invasion, number of lymph nodes resected, and number of lymph nodes with metastases (Table 1).

**Study Inclusion and Exclusion criteria**

Inclusion and exclusion criteria were defined as follows: patients were included when 1) histology confirmed an adenocarcinoma of the stomach or oesophago-gastric junction, and 2) the date of death or survival data were available. Patients were excluded when 1) histology identified a tumor type other than adenocarcinoma, 2) histopathological data were incomplete, 3) patients had previously undergone a resection of a Billroth-II stomach with cancer in the gastric remnant, and 4) date of patient death or survival data had not been recorded. Patients who received perioperative chemotherapy were also excluded.

### Histology and TNM classification

Tissue specimens were fixed in formalin and embedded in paraffin. Deparaffinized sections were stained with hematoxylin and eosin. Tumors were classified according to the Laurén classification [53] and the mucin phenotype [54]. All cases included in this study were re-examined by two surgical pathologists (VW, CR). pTNM-stage of all study patients was determined according to the 7^th^ edition of the UICC guidelines [55] and was based solely on surgical pathological examination including classification of distant metastases (pM-category). In the 7^th^ edition all tumors of the oesophago-gastric junction as well as tumors of the proximal 5 cm of the stomach with extension into the oesophagus are classified as oesophageal tumors [55]. Patients were re-categorized accordingly.

**Tissue micro array construction**

Formalin-fixed and paraffin-embedded (FFPE) tissue samples were used to generate tissue micro arrays (TMA) as described previously [56]. Three morphologically representative regions of the paraffin “donor” blocks were chosen. Tissue cylinders of 1.5 mm diameter were punched from these areas and precisely arrayed into a new “recipient” paraffin block. 2 µm sections of the TMA-blocks were cut for further analysis.

**DNA isolation from Formalin-fixed and Paraffin-embedded (FFPE) tissue**

Genomic DNA was extracted from FFPE tissue using the QIAamp DNA mini kit (Qiagen) following the manufacturer’s instructions. The integrity and amplifiability of the isolated DNA was evaluated by a qualitative size range PCR assay [57]. Tissue sections were manually microdissected prior to DNA isolation to enrich for tumor cells (>80%).

**Analysis of *GNAS1* mutational hotspots**

Sequence analyses of mutational hotspots in exons 8 (codon 201) and 9 (codon 227) of the *GNAS1* gene were performed by pyrosequencing on a PyroMark Q24 instrument (QIAGEN). Target fragments were amplified by PCR with the primers: *GNAS1* (exon 8), forward 5´- AATTTTGTTTCAGGACCTGCTTC-3´ and reverse 5´-TGACTTTGTCCACCTGGAACT-3´ and *GNAS1* (exon 9), forward 5´- GTCCCTCTGGAATAACCAGCTGTC-3´ and reverse 5´- CACTTGCGGCGTTCATCG-3´. The resulting PCR products were analyzed by agarose electrophoresis and sequenced using following sequencing primers: *GNAS1* (exon 8), 5´- GACCTGCTTCGCTGC-3´ and *GNAS1* (exon 9), 5´- TGTTTGACGTGGGTGGC-3.

**Microsatellite Instability Assay**

Microsatellite instability (MSI) was determined by comparison of the allelic profiles of the mononucleotide repeat markers BAT-25, BAT-26, NR-21, NR-24, and NR-27 in tumor and corresponding normal tissue as described elsewhere [18]. In brief: all markers were coamplified in a pentaplex PCR assay with the QIAGEN Multiplex PCR Master Mix (QIAGEN, Hilden, Germany) following the manufacturer´s recommendations for amplification of microsatellite loci. The amplified loci were analyzed on an ABI Prism 310 Genetic Analyzer (Applied Biosystems, Darmstadt, Germany). Samples were judged as microsatellite unstable (MSI) when the tumor showed instability in at least two of the five (40%) microsatellites analyzed.

**Immunohistochemistry**

Immunohistochemistry was carried out with a polyclonal antibody directed against GNAS1 (LifeSpan BioSciences; Seattle, North America; dilution 1:1000). Antigen retrieval was performed with 10 mM citrate buffer (pH 6.0) in a pressure cooker at 120°C for 10 minutes. After a blocking step with Hydrogen Peroxide Block (Thermo Scientific, Schwerte, Germany) the samples were incubated with the antibody at 4°C over night and the immunoreaction was visualized with the Histofine^®^ simple stain MAX PO Multi detection reagent (Nichirei Biosciences Inc.; Tokyo, Japan) in combination with the DAB Peroxidase Substrate Kit (Vector Laboratories Inc.; Burlingame, USA) according to the manufacturer’s instructions. Counterstaining was done with haematoxylin (Dr. K. Hollborn & Söhne GmbH & Co KG; Leipzig, Germany). Immunohistochemistry with the other antibodies, i.e. mucin 1, mucin 2, mucin 5, mucin 6, CD10, MLH1, PMS2, MSH2 and MSH6 was done as described elsewhere [18]. The specificity of the immunostaining was verified by using positive controls recommended by the manufacturers and by omission of the primary antibody.

The expression of GNAS was evaluated as follows: Any staining (e.g. weak, moderate or strong) of tumor cells was categorized as positive immunostaining. Absence of any immunoreaction was categorized as negative.

**Statistics**

Statistical analyses were performed using SPSS 20.0 (IBM Corporation). The significance of correlation between clinico-pathological parameters and biomarker expressions was tested using Fisher's exact test. For parameters of ordinal scale (T-category, N-category, tumor stage) we applied Kendall's tau test instead. A p≤0.05 was considered statistically significant.

**Bioinformatics**

**Mapping**

The Illumina WGS (whole genome sequencing) data from the patients with gastric cancer were mapped against the human genome reference hg19 (<http://genome.ucsc.edu>, [58] with BWA (v0.5.9). The seed length was set to 35 with maximum two differences in this subsequence, while for all other parameters the default options were used. This included a mismatch penalty of three, a gap opening penalty of four and a maximum edit distance of 0.04. The Solid WES (whole exome sequencing) reads were aligned with Bioscope (v1.2.1) using the default parameters. Reads with a mapping/pairing quality less than eight or an alignment-length/read-length ratio less than 0.85 were excluded from further analyses.

**SNVs and small indels**

The following parameters were used for the variant calling with GATK v1.3, Samtools v0.1.16 and DiBayes: DiBayes was executed with medium stringency, minimum mapping/pairing quality of eight, minimum color quality value of seven and minimum coverage of three reads with different start points. A support of the variant from reads on both strands was not required. Reads with an alignment-length/read-length ratio less than 0.85 were filtered. GATK was performed with the following steps and parameters: CountCovariates (training set: dbSNP build 132; covariates: ReadGroupCovariate, QualityScoreCovariate, DinucCovariate, CycleCovariate) -> TableRecalibration -> AnalyzeCovariates (ignoreQ=5) -> UnifiedGenotyper (stand_call_conf=50.0; stand_emit_conf=10.0; dcov=800; glm=SNV) -> VariantRecalibrator (mode=SNV; maxGaussians=4; percentBadVariants=0.05; an: QD, HaplotypeScore, MQRankSum, ReadPosRankSum, FS, MQ; training set: hapmap, 1000 Genomes, dbSNP build 132) -> ApplyRecalibration (ts_filter_level=99.0). Small indels were called with DiBayes with medium stringency in the human WES data and Samtools v0.1.16 with default parameters for the WGS data. All variants called on chromosome Y were excluded.

Annovar (version Jun 2011) was applied for the annotation of SNVs and small indels. A base substitution was classified as damaging, if either PolyPhen-2 or SIFT predicted an effect on the function of the protein. The PhyloP score rescaled by dbNSFP to [0.1] was applied for conservation information of each genomic position. Positions with a value above 0.9995 were defined as highly conserved. SNVs, which were not annotated in dbSNP build 132, were classified as novel. In addition, all variants detected in the NHLBI Exome Sequencing Project (v2) (ESP) or by the Exome Aggregation Consortium (v.0.3) (ExAc) were excluded from the final reported variant tables (Supplementary Table 2C-F and 3). The ExAc_Aggregated_Populations frequencies applied in our study were based on sequencing data sets of 60,706 unrelated individuals, which are part of disease-specific or population genetic studies of ExAc.

To calculate the allele counts for each position a file in pileup format was created with the Samtools v0.1.16 pileup command. Exclusively reads with a minimum mapping quality of 60 and bases with a quality score of at least 13 were considered. A threshold of 5% allele support was set for the existence of a SNV.

The investigation of base substitutions that were candidates for an association with gastric cancer were performed with the union of SNVs called with Samtools (WES, WGS) and DiBayes (WES), while SNVs called with GATK were only used for pathway analysis. For the filter steps on variant level SNVs and small indels supported by ≥5% in the WES as well as in the WGS data were considered. A SNV was defined as tumor-specific (somatic), if neither in the WES nor in the WGS data of the matching control sample ≥5% of the reads supported the variant. A small indel was classified as somatic, if in the WES and WGS data of the matching control sample less than five percent of the reads supported the identical indel or another indel at the same or an adjacent position. Indels were also excluded from the final filtered indel lists described in the Supplementary Tables 3A and 3B, if the exact same indel type was called in one of the 6 adjacent basepair positions in the matching control (WES or WGS).

The investigation of SNV patterns like rainfall plot or SNV type distribution was performed with all somatic SNVs called with Samtools in the WGS data.

**Description of split reads and read pairs supporting structural variations**

To perform a quality filter for the structural variants, split reads were extracted from unmapped sequences. Therefore, all reads with a quality score greater or equal than 35 at 95 or more positions were aligned against the human reference (hg19) using the program BLAT. If for a read a full-length alignment, i.e. mapping with at least 92 bases without gap, existed, all hits for this read were removed from the candidate list. Only reads with maximum two alignment parts were considered for further analysis. These two parts had to cover together at least 92 bases of the read with a maximum of five bases existing in both hits.

A large insertion was supported by a split read, if one part was included in the assembly of the contig and one part was located on the contig sequence, which overlaps with the genomic reference sequence. A read pair endorsed the insertion, if one read mapped to the genomic reference sequence in 800 bp or less distance of the insertion position and the other read was part of the contig assembly.

An interchromosomal translocation was supported by a split read, if both subsequences aligned in less than 200 bp distance from the breakpoints, respectively. A split read indicated an inversion, if one subsequence aligned outside of the inverted region with a maximum distance of 50 bp to the breakpoint, the other read part mapped within the structural variant and the two hits were on opposite strands. To support a deletion, one read part had to align before the deletion start and the second after the deletion end. The mapping start and end of the subsequences had to be in less than two basepairs distance of the breakpoints, respectively. Only reads with a gap of five or more basepairs between the two alignments were considered. A tandem duplication was supported, if the starting point of the second hit was before the starting point of the first hit, but both subsequences pointed in the same direction. Furthermore, both alignments had to be within the called duplicated region or the maximum distance had to be less than 20 bp.

An interchromosomal translocation was supported by a read pair, if at each breakpoint one read was located with less than 1000 bp distance. A read pair supported an inversion, if both reads were on the same strand and the alignment positions were less than 700 bp away from the respective breakpoints. To support a tandem duplication, both reads pointed toward the chromosome ends and the distance to the duplicated region was maximum 200 bp. Exclusively tandem duplication of a size smaller than or equal to 10,000 bp was investigated.

**Filter parameter for structural variations and large indels**

An interchromosomal translocation passed the quality filter, if at least 15 split reads (BLAT) or five percent of the pairs supporting the translocation at identified breakpoints existed. A putative large deletion was classified as reliable, if either (i) 40 or more reads supported the variation according to the Pindel output (mapped reads) or (ii) at least 15 unmapped split reads (BLAT result) existed at the called position. An inversion passed the quality filter, if the variant was supported by (i) 50 mapped reads based on the Pindel output, (ii) five or more split reads based on the BLAT result or (iii) more than 10% of the read pairs at each breakpoint. All tandem duplication with more than 10% supporting read pairs and greater than 30 split reads (Pindel or BLAT output) passed the quality filter. Two translocations were located in the same region, if the distance between the respective breakpoints covered 25,000 bp or less.

An interchromosomal translocation or inversion was defined as present in the control sample, if the positions of both breakpoints in the control samples were closer than 100 bp to the called position in the tumor samples. All deletions and tandem duplications having overlapping positions in the corresponding control samples were excluded as candidates for an association with gastric cancer. For this step all unfiltered variants called in the control samples were used.

A novel pipeline for the detection of large insertions was developed, which was based on mapped as well as unmapped reads. In the first step, all unmapped reads with a quality score above 20 at 80 or more positions were assembled with velvet v1.2.06 [50] in the tumor samples. The resulting contigs were aligned with BLAT v.34 [59] to the human reference to find putative insert sites. If at least 50 nt of the contig start and 50 nt of the contig end matched to genomic regions with less than five bases distance, the position was considered as putative insertion site. The aligned sequences had to be less than 10 nt away from the contig start/end. Insertions smaller than five bases were excluded. All unmapped reads of the corresponding control samples were mapped against all velvet contigs of the tumor sample. Contigs, which were covered at 90% or more positions, were excluded from the analysis. To pass the quality filter step, the insertion had to be supported in the tumor samples by at least five read pairs and each insertion junction by 25 or more reads.
